# Current research on the design, properties and applications of tribological materials: a review

**DOI:** 10.1039/d5ra02780b

**Published:** 2025-09-22

**Authors:** Na Xiao, Jun Tang, Shengfei Zhou, Yulong Shi, Fang Qian, Shuaichao Qiu, Yahui Chen, Dian Zhao, Kang Yang

**Affiliations:** a Faculty of Engineering, Huanghe Science and Technology University Zhengzhou 450000 China; b Hunan Province Key Laboratory of Materials Surface/Interface Science & Technology, Central South University of Forestry & Technology Changsha 410004 China; c School of Mechanical Engineering, Liuzhou Institute of Technology Liuzhou 545616 China wwwzhoushengfei@163.com +86-372-2986271 +86-372-2986271; d School of Mechanical and Aviation Manufacturing Engineering, Anyang Institute of Technology Anyang 455000 China

## Abstract

The development of novel materials with excellent wear resistance and anti-friction properties has become a prominent focus of research. The design and fabrication of such materials is expected to make a significant contribution to energy conservation; they may reduce energy losses caused by friction and wear by approximately 40%. This paper provides a comprehensive review of the latest advancements in tribological materials in terms of design, properties, and applications. It first summarizes the design strategies of tribological materials from two aspects: surface engineering and matrix strengthening. Subsequently, it explores the relationship between the wear resistance of materials and intrinsic properties such as hardness, stiffness, strength, and cyclic plasticity. Furthermore, it introduces the application of tribological materials in aerospace components, automotive parts, wind turbines, micro-/nano-electromechanical systems, atomic force microscopes, and biomedical devices. Finally, the paper discusses future challenges and potential development directions in this critical research area.

## Abstract 2

The development of novel materials with excellent wear resistance and anti-friction properties has become a prominent focus of research.

## Graphical abstract

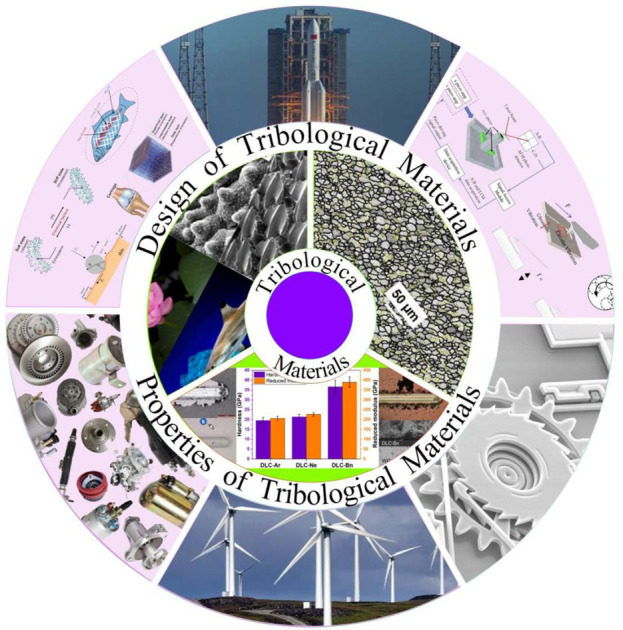

## 1 Introduction

Tribology is dedicated to investigating friction, wear, and lubrication that occur between interacting surfaces. Among these, the friction and wear are particularly significant, as they involve continuous deformation, surface damage, and, in some cases, material removal from the contacting surfaces. These processes have a significant impact on the service lifetime of mechanical components across scales, from the nano-to the macro-scale.^1^ For example, rapid wear of the scanning probe tip in a multi-function tribological probe microscope (TPM) operating in high contact force testing mode may reduce the reliability of mechanical property evaluations of the tested materials. Total ankle replacement may fail due to prosthesis-related friction, wear, fracture, or loosening, which can cause joint deformation.^2^

Material wear mechanisms can generally be classified into the following categories: adhesive wear,^3^ abrasive wear,^4^ fatigue wear,^5^ corrosion wear,^6^ and fretting wear.^7^ The development of wear-resistant and anti-friction materials has long been a key objective, aimed at minimizing material damage or the loss that mainly results from wear and friction. Wear processes can be classified into three states: low wear, light wear, and severe wear.^8–10^ Ultra-low wear can be achieved through the formation of a transfer film on the abraded polymer surface.^11^ In light wear, shallow surface scars and microcracks are typically observed.^12^ In severe wear, changes in operating parameters such as pressure or sliding velocity are commonly observed.^13^

A variety of approaches have been employed to prevent the transition from low to light wear, or from light to severe wear. Surface engineering is one of the most effective strategies for mitigating wear, as wear is fundamentally a surface phenomenon, and material deterioration closely depends on its surface properties and structures. Surface engineering encompasses a range of physical and/or chemical processes aimed at modifying the surface and subsurface layers, which do not alter the overall properties or structures of the materials. For example, physical surface modification has been proven to effectively enhance the surface hardness and tribological properties of Si alloy coatings.^14^

The techniques are employed to achieve surfaces with high tribological properties typically include laser processing, electrochemical processing, mechanical processing, chemical processing, plasma processing, gas nitriding, and ion implantation.^15–21^ These techniques can be categorized into four types, each using a distinct approach to enhance the surface tribological properties: coating preparation, surface texturing, surface hardening, and architecture design.

In addition to surface engineering, the matrix strengthening has also been proven to be effective for enhancing the tribological properties of materials. In this study, “matrix strengthening” refers to the strategies that improve the tribological properties of bulk metals, ceramics, or polymers. This can be achieved by controlling composition, designing microstructures, and adding reinforcements. The optimization of the approach relies on tailoring deformation mechanisms^12^ or the introduction of hard *in situ*/*ex situ* nanoparticles.^13^ These particles play a key role in enhancing the mechanical strength and hardness of the matrix. The uniformity of their distribution in the matrix and their wettability with respect to the matrix significantly influence the enhancement efficiency.

**Fig. 1:**
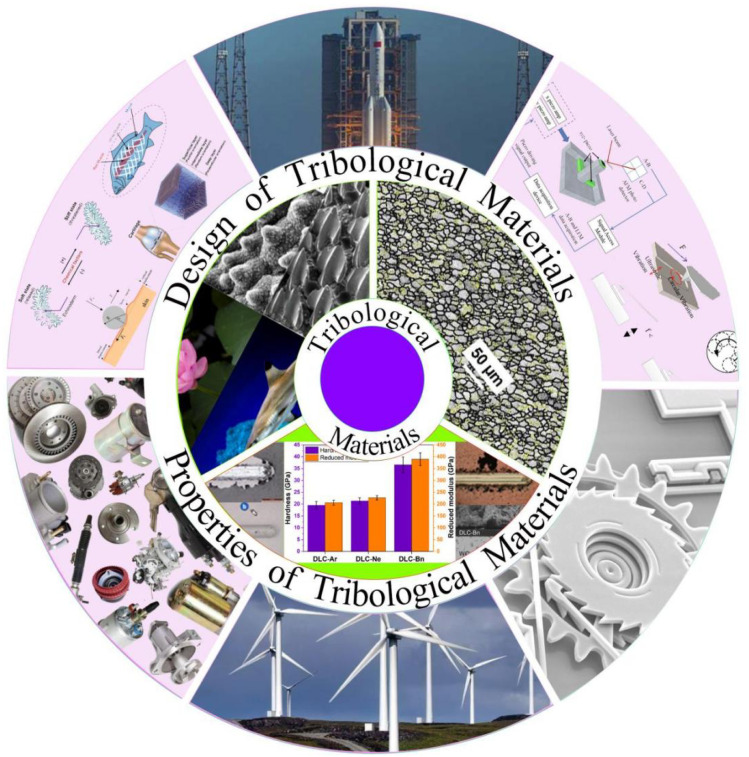
Design, properties, and applications of wear-resistant and anti-friction materials. The figure was reproduced from ref. 22–28 with permission from the rightsholder.

It is noteworthy that several reviews have focused on the preparation and tribological properties of hard coatings,^14^ anti-wear polymers and their composites, high-temperature self-lubricating materials, and other related topics. Given the rapid advancement of wear-resistant and anti-friction materials and their significant impact on energy consumption, it is crucial to provide a timely review of recent developments in this field. This paper presents a comprehensive review of critical issues related to the novel designs, key properties, and wide applications of the wear-resistant and anti-friction materials, as shown in Fig. 1. After a brief introduction in this section, Section 2 summarizes typical strategies in surface engineering and matrix strengthening for the development of wear-resistant and anti-friction material. Section 3 explores the correlation between a material's tribological property and its intrinsic behavior, such as hardness, stiffness, strength, and cyclic plasticity, and discusses the underlying mechanisms, such as strain hardening effect, size effect, dislocation nucleation, and grain boundary sliding. Section 4 illustrates the extensive applications of tribological materials in aerospace components, automobile parts, wind turbines, micro-/nano-electromechanical systems (MEMS/NEMS), atomic force microscopy (AFM), and biomedical devices. Finally, Section 5 presents key conclusions and addresses the current challenges and future research directions in this critical field.

## 2 Design strategies of tribological materials

Enhancing the tribological performance of materials depends on precise modulation of their composition and microstructural design. Current studies in this field primarily focus on advances in surface engineering and matrix reinforcement strategies. This approach facilitates the achievement of enhanced tribological properties in the surface layer relative to the substrate. Further, favorable control and design of composition and structure help achieve the high wear-resisting and anti-friction properties without compromising the mechanical properties of the materials. To achieve the desired performances, a significant disparity is essential, and this will be elucidated in the following sections.

### 2.1 Surface engineering

#### 2.1.1 Coatings

**Fig. 2:**
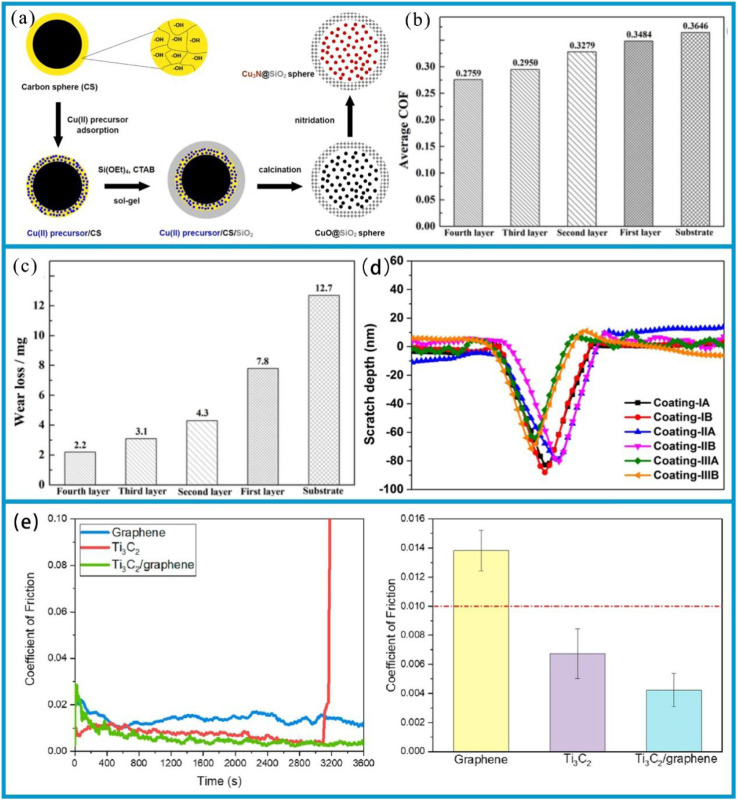
(a) Schematic of the synthesis process of hollow mesoporous silica spheres. Reproduced from ref. 30 with permission from Elsevier, copyright 2021. (b) Friction coefficient of the fourth layer and the substrate. (c) The wear volume of the fourth layer and the substrate. Reproduced from ref. 35 with permission from Elsevier, copyright 2022. (d) Two-dimensional cross-sectional scratch profiles of scratch trajectories of various plasma-sprayed coatings. Reproduced from ref. 36 with permission from Elsevier, copyright 2019. (e) Instantaneous friction coefficient plots and average friction coefficient histograms generated by rubbing graphene, Ti_3_C_2_, and Ti_3_C_2_/graphene coatings against DLC-coated steel balls. Reproduced from ref. 37 with permission from Elsevier, copyright 2021.

The deposition of resistant coatings often effectively enhances surface durability. These coatings have been successfully deposited on their substrates using a variety of techniques, including physical vapor deposition (PVD) (Fig. 2a),^29,30^ chemical vapor deposition,^31^ hybrid physical-chemical vapor deposition,^32^ and thermal spray methods.^33^ Previous review works have documented the preparation processes, microstructures, mechanical properties, and strengthening-toughening mechanisms^34^ associated with these coatings.

**Fig. 3:**
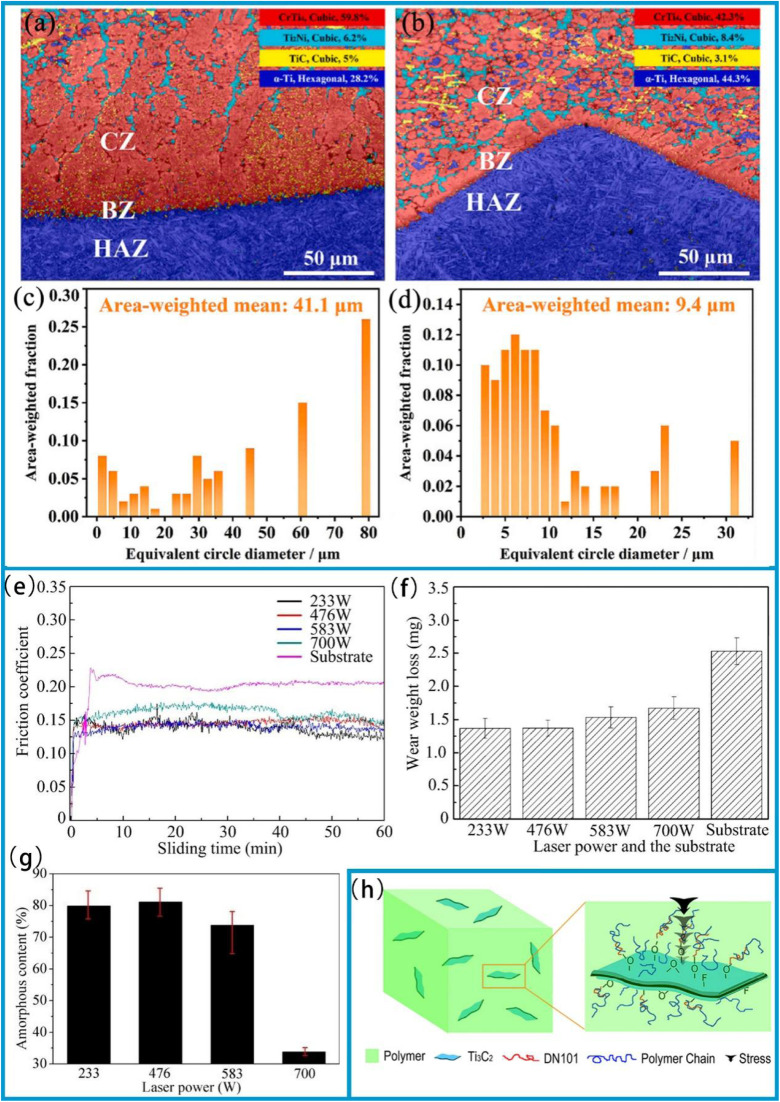
(a) BC + phase map of the single-track region. (b) BC + phase map of the multi-track overlapping region. (c) Grain size statistics in the single-track region. (d) Grain size statistics in the multi-track overlapping region. Reproduced from ref. 41 with permission from Elsevier, copyright 2022. (e) Variation process of friction coefficient along time, (f) wear weight loss of different tested surfaces. (g) The amorphous content obtained from XRD patterns. Reproduced from ref. 43 with permission from Elsevier, copyright 2019. (h) Schematic diagram of the principle of Ti_3_C_2_ in enhancing mechanical properties. Reproduced from ref. 42 with permission from Elsevier, copyright 2016.

To achieve high wear resistance and anti-friction properties of the coatings, several strategies have been employed, including ion implantation of selected additive species, ion beam mixing of thin deposited coatings, and ion-beam-assisted deposition of thicker overlay coatings.^38^ Additionally, the carbide phase in carbide-based coatings can enhance their tribological properties.^39^ Tungsten carbide (WC) offers advantages such as a high melting point, high hardness, low thermal expansion coefficient, and good wettability with nickel-based alloys, making it an ideal ceramic reinforcing material.^40^ In this study, a WC-reinforced Ni-based gradient composite coating was fabricated on a Q345R steel substrate *via* laser cladding. The Ni-WC composite coating was designed as a multi-layer structure with a gradient composition.^35^ In the designed four-layer gradient structure, the WC content gradually increases from 10% to 50% layer by layer. This layer-by-layer transition approach can effectively reduce the difference in thermal expansion coefficients between layers, thereby alleviating the interfacial stress concentration. Hardness test results show that the microhardness of the gradient composite coating gradually decreases from the surface to the substrate. Friction and wear test results (Fig. 2b and c) show that the coating's wear resistance improves with increasing WC content. Among these layers, the average friction coefficient of the fourth layer coating is 0.2759, which is 24.3% lower than that of the substrate (0.3646), and its wear loss is 2.2 mg, 82.7% lower than that of the substrate (12.7 mg). This result is attributed to the supporting effect of the WC hard phase, which reduces plastic deformation of the substrate, and the strengthening effect of the carbide phase, which inhibits the ploughing effect during abrasive wear. The role of such strengthening phases is essentially related to the composition design and microstructure control of the coating. In fact, the hardness of the coating largely depends on its composition and microstructure.^41^ For example, an enhanced surface composite coating with improved nano-hardness is successfully prepared on the Ti811 alloy *via* laser cladding. A comprehensive investigation was conducted to examine the composite coating's microstructure, phase composition, interface structure, and nano-hardness. It was observed that the single-track and multi-track overlapping regions of the coating exhibited significant microstructural differences due to different cooling rates; however, the coating overall formed a good metallurgical bond with the substrate. As shown in the BC + phase diagram of the BZ (bonding zone) in Fig. 3a and b, both the single-track and multi-track overlapping coatings achieved metallurgical bonding with the substrate; additionally, the microstructure of the multi-track overlapping coating was more refined than that of the single-track coating. Specifically, the grain size (equivalent circle diameter) of the BZ in the single-track region was 41.1 μm, while that in the multi-track overlapping region was only 9.4 μm as shown in the grain size statistics of the BZ in Fig. 3c and d. The phenomenon is attributed to the faster cooling rate of the already solidified coating during secondary melting in multi-track overlapping regions, and to the lower ratio of temperature gradient to solidification growth rate (*G*/*R*) as well as the higher product of temperature gradient and solidification growth rate (*G* × *R*) in this region, which jointly promote the formation of fine grains.^41^ Furthermore, effective interfacial bonding was identified as a crucial factor in ensuring the coating's enhanced nano-hardness.

The hardness of coatings can be increased through processes such as spinodal decomposition or phase precipitation, which are common phenomena in ternary, quaternary, and multinary transition metal nitrides due to their miscibility gaps. The application of metal-based solid self-lubricating coatings is a pivotal approach for mitigating friction and wear at tribological interfaces, and the coatings' wear resistance and anti-friction properties are intrinsically linked to their mechanical properties.^44^ The enhanced performance of these coatings can be attained by incorporating Mo-containing compounds and other additional dopants to match the lubricants used on rubbing surfaces, either by modifying surface energy or by matching the stiffness of mechanical components.^45^ Research shows that doping with Ti,^46^ Cr,^47^ Zr,^48^ Ni,^49^ Ti-Si,^50^ or Sb_2_O_3_ (ref. 51) can enhance the hardness of the MoS_2_ coating. Meanwhile, it induces deformation of the coating's crystal structure, thereby improving its wear resistance and anti-friction properties. Specifically, these dopants can inhibit the growth of MoS_2_ columnar crystals, form amorphous or nanocomposite structures, reduce porosity, increase coating density, and thus reduce oxidation degradation and abrasive wear. They also further enhance hardness and elastic modulus through solid solution strengthening and dispersion strengthening, improve the coating's resistance to plastic deformation, and extend wear life. During friction, they promote the formation of basal plane-oriented lubricating transfer films, reduce the friction coefficient, and improve its stability. Additionally, the formed passive layers (such as TiO_2_, Cr_2_O_3_, ZrO_2_) can block moisture and oxygen, thus enhancing the coating's oxidation resistance in a humid environment.

The effects of different dopants vary: Ti excels in lubrication and fatigue resistance due to nanocrystalline dispersion and enhanced interfacial bonding, making it particularly suitable for rolling contact conditions; Cr offers advantages in heavy-load and corrosive environments owing to its high hardness and stable oxide film; Zr achieves low friction and long service life across a wide load range through the synergy of an amorphous matrix and nanocrystals; Ni exhibits significant solid solution strengthening effects but requires controlled content to balance brittleness; TiSi's multi-layer nanostructure optimizes dislocation hindrance and stress release, providing more stable performance under complex loads; Sb_2_O_3_'s amorphous matrix and dispersed nanoparticles maintain low friction in both dry nitrogen and humid environments.

Overall, the introduction of dopants not only reduces the friction coefficient of the MoS_2_ coating but also enhances its hardness, density, and oxidation resistance. This has also been confirmed by relevant studies, which show that doping MoS_2_ with metals and oxides enhances friction behavior in humid environments.^52^ Additionally, Ti, Au, and Sb_2_O_3_ have been identified as effective dopants for improving the tribological performance of MoS_2_.^52^ It is worth noting that systems with a greater number of element types, such as ternary or quaternary systems, can exhibit pronounced segregation in the two binary compounds. Thermodynamically driven “compositional modulation” results in an isotropic coating, which enhances both the mechanical properties and tribological properties of the coating.

A self-organization method in sliding processes plays a pivotal role in the design of coatings and other wear-resistant and anti-friction materials.^53^ When the coating adapts to friction and wear, its surface microstructure undergoes a significant transformation. In accordance with the principle proposed by I. Prigogine, this transformation is a response to external stimuli. This implies that the second law of thermodynamics does not hinder the formation of highly organized dissipative structures in open friction systems. A self-organizing structure forms during the friction run-in stage. The early formation of these structures can effectively reduce the coating wear. Fox-Rabinovich^54^ highlighted that non-equilibrium coatings are susceptible to self-organization, which can be achieved through high-energy ion impact in modern surface engineering techniques such as physical vapor deposition. A non-equilibrium state, which is associated with the complexity of the coating, can be adjusted *via* elemental doping, the formation of solid solutions and binary or ternary compounds, and the use of multilayer structure. It is recommended that future coating designs further reduce the complexity and non-equilibrium states to enhance their resilience for the external shocks, and facilitate an adaptation to varying operational conditions.

In this context, recent advancements in the synthesis of ZnO nanoparticles with three distinct morphologies have targeted ZnO as the core component for developing a ZnO@graphene core–shell nanoadditive. Sheet-like morphology of ZnO is considered particularly advantageous, and the ZnO@graphene hard core-soft shell nanostructure is deemed to be well-suited for dynamic friction environments. The synergistic effect of this nanostructure effectively maintains the load-bearing capacity and stability of the lubricating film, thus exhibiting superior tribological properties. Compared to single additives, these composite additives demonstrate the enhanced thermal stability, improved dispersibility and increased load-bearing capacity. Notably, in the friction process, the ZnO nanoparticles modified with oleic acid can form a Zn-containing boundary lubricating film to isolate the metal surface. The tiny ZnO nanoparticles can exert a micro-bearing effect; they quickly enter the space between two friction pairs and transform sliding friction into rolling friction. Under the shear stress, ZnO nanoparticle clusters fragment and refine, filling into the concave and convex areas on the friction surface to reduce surface roughness. Under high-load conditions, ZnO nanoparticles melt or semi-melt, enable them to fill the worn surface. Therefore, they show excellent anti-friction and wear-resistant properties under various lubrication conditions. In a comparative study, a 0.5% ZnO nanosuspension outperformed CuO and ZrO_2_ nanosuspensions, reducing the friction coefficient and wear loss in polyalphaolefin lubricants.^55^ This research underscores the potential of self-organizing and nanostructured materials to revolutionize tribology by offering the innovative solutions to wear and friction.

The development of innovative wear-resistant coatings places special emphasis on unique structural designs, including gradient structure coatings, multi-scale structure coatings, and layered structure coatings.^34^ The usage of thermal spraying with a hybrid powder-suspension feedstock represents a novel approach to conveniently fabricate coatings with distinct chemistry and unique microstructures. Suspension plasma spray (SPS) is a relatively recent variant of the plasma spray process, enabling the use of fine sub-micron and nano-sized powders suspended in alcohol or water to deposit high-performance coatings. Berghaus *et al.*^56^ first demonstrated the potential of SPS as a processing route for depositing WC-12Co coating, which improved the hardness and crystallinity of the deposited coatings. In hybrid coatings, splats derived from powder and suspension feedstocks can be deposited onto the substrate either simultaneously or sequentially to obtain the composite, layered, or functionally graded coatings.^57^ In this context, a case study was conducted using Triboloy-400 (T-400), a cobalt (Co)-based alloy renowned for its wear and corrosion resistance.^58–60^ T-400 was selected as the candidate material in the powder form, while chromium carbide (Cr_3_C_2_) was chosen as a suspension material. T-400 consists of a hard, wear-resistant intermetallic laves phase dispersed in the softer Co-rich solid solution matrix.^61–63^ Furthermore, coatings based on high-temperature resistant compounds such as Cr_3_C_2_ have been proven to have excellent erosion and wear resistance. For instance, the erosion rate of a Cr_3_C_2_–NiCr (85/15)% coating at a 75° impact angle is only 2.8 × 10^−7^ mg g^−1^, mainly attributed to its extremely high hardness.^64–66^

Currently, Fe-based amorphous composite coatings can be produced using several surface modification techniques, including thermal spray and laser cladding.^43,67,68^ Among them, optical laser processing technology has been successfully applied to prepare the crack-free Fe-based amorphous composite coatings on stainless steel substrates. X-ray diffraction analysis shows that the composite coating is mainly composed of Fe (Cr, Mo) solid solution, and contains a small amount of Fe_5_C_2_ and Fe_23_B_6_ reinforcing particles. During laser processing, the content of amorphous phase material decreases with increasing laser power, as shown in the XRD-derived results of amorphous substance content in Fig. 3g; and there is a clear correlation between its content and the coating's friction and wear properties: coatings with lower amorphous phase content have higher friction coefficients (Fig. 3e); meanwhile, the weight loss from friction and wear is also greater (Fig. 3f). However, when the amorphous phase content in the coating increases and is combined with an *in situ* nanocrystalline strengthening, its microhardness increases significantly, which is conducive to improving the wear resistance of the coating.

In a related study, the initial porosity of Fe-based amorphous coating was as high as 2.44%,^69^ but it could be reduced from 2.44% to 1.22% through heat treatment, indicating that heat treatment can effectively eliminate the pores in the coating. The reduction of porosity can significantly enhance the corrosion resistance of Fe-based amorphous coating. Specifically, a lower porosity can reduce the penetration channels for corrosive media and extend their penetration paths, thereby preventing the media from contacting the substrate. Meanwhile, a densified coating can fully leverage the inherent chemical stability derived from the characteristics of the amorphous structure, such as continuous formation of a passive film and the absence of grain boundaries, which makes it less prone to local corrosion and avoids the vicious cycle of “corrosion-product expansion-coating cracking-more severe corrosion” caused by media penetration. This contrasts with the amorphous coatings produced by laser cladding, which possess a compact microstructure and strong metallurgical bonding to the substrate due to sectional melting of the substrate.^70,71^ It further emphasizes the superior structural integrity achievable through optical laser processing.

Compared with conventional steel, the atomic configuration of Fe-based amorphous alloy exhibits a combination of long-range disorder and short-range structural order. Consequently, the Fe-based amorphous alloys demonstrate enhanced corrosion resistance relative to crystalline materials, attributed to the absence of grain boundaries, dislocations, and interfacial defects.^72^ Notably, the high strength and exceptional wear resistance can be achieved through long-range disordered structures: Fe-based amorphous alloys exhibit significantly superior fretting wear resistance to tool steel at temperatures below 573 K.^73^ In addition, the Fe-based amorphous coating sprayed with three plasma layers at a power of 30 kW exhibits excellent wear resistance and friction reduction effects, with the two-dimensional interface scratch contour depth for coating-IIIA shown in Fig. 2d. This is mainly attributed to the fact that the absence of grain boundary and dislocation defect reduces plastic deformation and adhesive wear; the dispersed nanocrystalline phase enhances hardness; the low porosity reduces the intrusion of abrasive particles and stress concentration; and the presence of nanocrystals inhibits a crack propagation. This coating has the smallest wear volume (only 7.3 × 10^−21^ m^3^) and the highest wear resistance coefficient (reaching 6.87 × 10^12^ Pa).^36^ The use of Fe-based amorphous coating has been shown to effectively extend the lifespan of coated workpieces while simultaneously reducing maintenance costs. As a result, these coatings have been widely adopted across diverse industries, including energy, chemical, aerospace, shipbuilding, as well as the boilers and gas turbines.

The development of HVOF-sprayed Fe-based amorphous coating has been accelerated over the past two decades. In 1996, Otsubo *et al.*^74^ successfully prepared Fe–Cr-based amorphous coatings, which exhibited the superior corrosion resistance to that of stainless steel in sulfuric and hydrochloric acid environments. The high corrosion resistance can be attributed to the interlayer structure and the absence of pores and inclusions after the coating deposition, which impedes the diffusion of the corrosion media. The formation of the amorphous phase is attributed to the extremely rapid ejection speed of the molten droplets and the high cooling rate during the spraying process. With advancements in the thermal spraying technologies, the amorphous ratio in the coating has increased to over 80%.

The wear resistance and friction coefficient of coatings depend on their hardness and thickness, respectively. The internal residual stress of coatings, which is induced by fabrication and post-treatment processes, has a significant impact on their wear resistance. One of the most suitable methods for depositing a-CN coatings is pulsed vacuum-arc sputtering of graphite.^75–77^ This method enables the fabrication of coatings with the requisite thickness by determining the desired number of pulses and regulating the thermal load on the substrate through modification of a pulse repetition rate. Furthermore, there is no need to apply an accelerating potential to the substrate.

Two-dimensional (2D) materials, including transition metal dicarboxylates and graphene, are of interest as materials for new wear-resistant and anti-friction coatings. The high mechanical strength, excellent lubricity, and thermal stability of commonly used graphene-based materials make them be candidates for the coatings in vehicles, especially aircraft and ships, where they provide lightweight and highly tribological properties under shear forces.

The use of different coating techniques, including spin-coating, spray-coating, and dip-coating, in conjunction with casting processes such as drop-casting, has been proposed as a means of rapidly preparing membranes. To enhance the uniformity and stability of the coating, and thus of the resulting GO membranes, it is advisable to use substrates that have an opposite charge to that of the GO nanosheets or that have surface functional groups capable of reacting with the GO nanosheets. In this context, the solvent evaporation rate and deposition speed are crucial parameters for the preparation of high-quality membranes. A spin-coating process results in the formation of an ultrathin laminar GO membrane, achieves through the uniform distribution of the GO suspension under centrifugal force. Although this method is only applicable to the fabrication of flat-sheet membranes, it has been demonstrated to enhance permeance characteristics by sevenfold relative to pressure-assisted filtration.^78^

Newly developed MXenes, with their high surface area, easily form lubricating or transfer films, thus reducing friction and wear rates. In 2021, MXenes achieved super-strong lubrication in both solid and liquid lubrication systems, exhibiting exceptional lubricating effects.^37,79,80^ MXene composite coatings are more effective in reducing friction than single MXene coatings. This is because the two-dimensional layered structure of MXenes is prone to interlayer sliding, and their surface function groups can reduce adhesion. When combined with graphene, the graphene forms an additional lubricating layer, protecting the stable structure of MXenes and reducing their degradation. As shown in Fig. 2e, the Ti_3_C_2_/graphene composite coating has a lower average friction coefficient (0.0042 ± 0.001) compared to Ti_3_C_2_ and graphene coatings. Moreover, the friction coefficient of the Ti_3_C_2_/graphene composite coating continues to decrease during frictional wear before finally stabilizing. In addition, the transfer film formed during the friction process can effectively disperse the load, and the lamellar structure can self-adjust to repair, reducing a wear rate by approximately half (to 4.5 × 10^−9^ mm^3^ (N^−1^·m^−1^)).^37^ Much attention has also been paid to the mechanical properties of MXene films. While several factors affect these properties, MXene-polymer composite films, in particular, exhibit the enhanced mechanical performance.^42,81^ For instance, in polymer films containing Ti_3_C_2_, the Ti_3_C_2_ nanomaterials located at a center of spherulites can not only enhance the crystallinity of ultra-high molecular weight polyethylene (UHMWPE) and absorb more energy, but also dissipate stress and impede crack propagation by disrupting the physical cross-links between DN101 and UHMWPE chains and transferring stress to themselves. As a result, the mechanical properties of UHMWPE are significantly enhanced. The schematic diagram of Ti_3_C_2_ reinforcing the mechanical properties of UHMWPE is shown in Fig. 3h.^42^ Interlayer distance, functionalization, and stacking type have a significant impact on the lubricating performance of MXenes. A larger interlayer distance, oxygen functionalization (M_2_), carbon monoxide (CO)_2_, and mirror stacking are associated with better tribological properties. These studies theoretically demonstrate the excellent lubrication potential of MXenes.

The anti-wear coating is designed to enhance the durability of the surface and can be applied through technologies such as PVD, chemical vapor deposition, and thermal spraying. These deposition techniques can optimize performance through methods such as ion implantation, precipitation formation, and the introduction of lubricants like MoS_2_. Among them, adding specific elements *via* ion implantation or spray mixing can effectively enhance the coating's wear resistance; the precipitation formation process enables the formation of a hard phase within the coating, which helps improve its mechanical properties, and then the introduction of self-lubricating materials can further reduce friction, thereby synergistically enhancing the coating's anti-wear effect.

The gradient composite coating has different components within its thickness range, provides customized performance for the various applications, and features strong interfacial bonding strength, balanced comprehensive performance, and excellent thermal and shock resistance. It can be applied to high-temperature components, heavy-load machinery, and corrosive environments. The nano-hardness coating exhibits extremely high hardness at the nanoscale, thereby enhancing the material's wear resistance. It is widely applied to components such as bearing races to improve surface scratch resistance. However, these two coatings are costly and not suitable for large-scale production. In contrast, solid lubricant coatings offer strong environmental adaptability and relatively low overall cost, making them widely applicable in the mechanical manufacturing, aerospace, and special equipment. That said, they have lower load-bearing capacity and require regular replenishment or replacement. In addition, high-end processes for them involve the high production costs and small production scales. The Fe-based amorphous coating has high hardness, corrosion resistance, and low cost, and can be fabricated on a large scale. It is applied in industrial equipment, municipal facilities, *etc.*, but has limited temperature resistance and lower toughness than metal substrates such as carbon steel, so it should be avoided in high-temperature and severe impact scenarios. Therefore, future coating design can focus on integrating the advantages of various coatings, for instance, combining the low cost of Fe-based amorphous coatings with the performance customization capabilities of the gradient composite coatings, to achieve the better application effects. Table 1 summarizes the information about the above-mentioned different surface coatings and their corresponding tribological behaviors.

**Table 1:** Information on different surface coatings and their corresponding tribological behaviors

Coating material	Hardness	COF	Wear rate or friction loss	Test conditions	Ref.
WC-Ni	1053.5 HV_0.2_	0.2759	2.2 mg	20 N, 400 rpm, 60 min, Si_3_N_4_ balls	35
Ni-G	6.85 ± 0.51 GPa	0.17	1.94 × 10^−3^ mm^3^ N^−1^ m^−1^	52 MPa, 100 rpm, 3.14 mm^2^, 24 °C, 1 Cr_15_Ni_4_Mo_3_N steel	44
Ni–MoS_2_	7.41 ± 0.73 GPa	0.61	2.58 × 10^−3^ mm^3^ N^−1^ m^−1^		
Cu–Sn	2.74 ± 0.27 GPa	0.61	0.62 × 10^−3^ mm^3^ N^−1^ m^−1^		
MoS_2_/Ti–MoS_2_/Si	7.53 GPa	0.0432	3.2 × 10^−7^ mm^3^ N^−1^ m^−1^	8 N, relative humidity 45%, 20 °C, 5 Hz, amplitude: 5 mm, the loop count is 10 000 times, GCr15 steel balls	50
Cr_3_C_2_–NiCr (95/5)%	1065 HV_0.1kg_	—	4.4 × 10^−7^ mg g^−1^	3 kg, 650 °C, impact angle: 75°, particle velocity: 80 m s^−1^, Al_2_O_3_ particles, feed rate: 5 g min^−1^, 10 min	64
Cr_3_C_2_–NiCr (90/10)%	875.33 HV_0.1kg_	—	3.2 × 10^−7^ mg g^−1^		
Cr_3_C_2_–NiCr (85/15)%	621.33 HV_0.1kg_	—	2.8 × 10^−7^ mg g^−1^		
Al_2_O_3_–40ZrO_2_	900 HV_0.3_	0.03–0.04	0.07 mm^3^ m^−1^	5 kg, 100 N cm^−2^, contact area: 16 mm^2^, mineral oil	66
ZrO_2_–MgO	777 HV_0.3_	0.155–0.190	—		
AlCrN–TiAlN	33.0 ± 8.5 GPa	0.2	—	Cutting speed: 160 m min^−1^, feed rate: 15 275 mm min^−1^, cutting depth: 0.3 mm, cutting width: 4 mm	54
Ti_3_C_2_/MXene	—	0.0067 ± 0.0017	4.9 × 10^−7^ mm^3^ (N^−1^ m^−1^)	2 N, 0.1 m s^−1^, 1 h, dew point temperature: −40 °C, relative humidity: 0.1%, indoor temperature, nitrogen atmosphere	37
Ti_3_C_2_/graphene	—	0.0042 ± 0.0011	4.5 × 10^−9^ mm^3^ (N^−1^ m^−1^)		

#### 2.1.2 Surface texturing

**Fig. 4:**
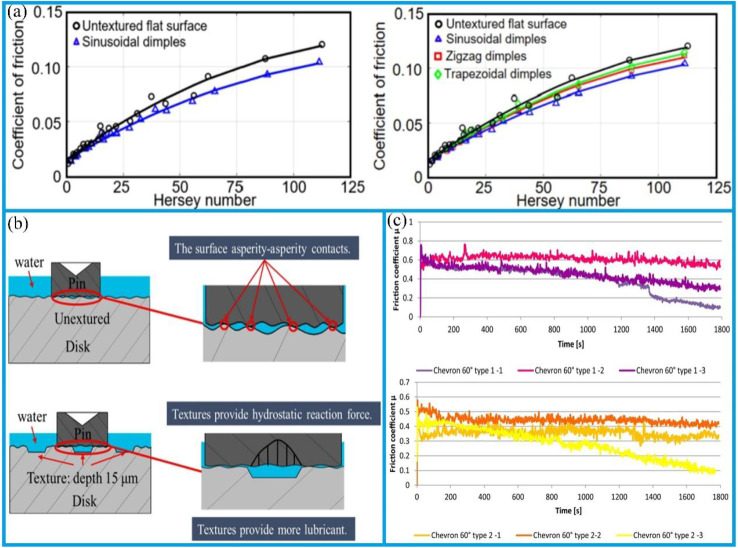
(a) A comparison of COF with the Hersey number for the untextured reference sample, sinusoidal indentations, and other surface textures. Reproduced from ref. 88 with permission from Elsevier, copyright 2019. (b) A schematic representation of the friction and wear mechanisms of the untextured and ungrained textured samples. Reproduced from ref. 91 with permission from Elsevier, copyright 2022. (c) A plot of the instantaneous coefficient of friction for Type 1 and Type 2 textured disc assemblies at an angle of 60° between the arms. Reproduced from ref. 92 with permission from Elsevier, copyright 2020.

Surface texturing is an effective technique employed to create a surface with a desired patterned configuration. It is a widely used method for modifying mechanical and tribological properties, including enhancing fatigue strength, corrosion resistance, wear resistance, hydrophobicity, load-bearing capacity, and resistance to both biological contamination and fouling.^82^ It has been demonstrated that surface texturing can enhance tribological properties, facilitate light capture in solar cells,^83^ improve biological implants,^84,85^ and facilitate a production of superhydrophobic coating.^86–88^ Laser surface texturing, in particular, has been shown to markedly enhance the wettability of materials.^8^ The application of surface texturing also supports the enhancement of lubricating coatings, thereby enabling the development of superhydrophobic coatings.^88^ It has been demonstrated that the introduction of sinusoidal dimples can reduce friction, an effect that becomes particularly pronounced under elevated Hersey numbers (Fig. 4a). These dimples are capable of generating additional hydrodynamic pressure, enhancing the load-carrying capacity of the lubricating film, reducing the flow resistance of lubricant, and promoting a more homogeneous distribution of the lubricant. Additionally, sinusoidal indentations can store lubricating oil and continuously supply it to the friction area, thereby effectively preventing lubricant deficiency, and further mitigating the wear. Furthermore, the geometry of the texture exerts a minor influence on the friction response, as illustrated in Fig. 4a. For each Hersey number, the friction reduction is more pronounced for sinusoidal dimples, followed by the sawtooth and trapezoidal dimples. Cavitation typically occurs when a fluid undergoes rapid changes in pressure. The resulting pressure build-up is asymmetric, leading to a positive net pressure build-up. When discussing this phenomenon, it is essential to consider the role of “inlet suction” due to the negative pressure in the dimple, as well as the impact of density changes and inertial effects.

Surface texture processing^89^ involves modifying the surface by creating moulding, micro-groove, micro-dent, and micro-channel. It is typically accomplished through laser-based techniques, micromachining, and other related methods applied to the substrate surface. Among these approaches, laser surface texturing is the most widely used technique in the academic and industrial research, due to its high controllability, high accuracy, environmental friendliness, high efficiency, and the capability to operate under ambient conditions of temperatures and pressures. LST involves absorbing laser energy and can remove material through rapid melting or vaporization. Laser surface treatment offers the better repeatability, good cost-effectiveness, and no pollution.^90^ What's more, stainless-steel samples processed by LST exhibit various textural patterns, including circles, triangles, and diamonds. These samples are characterized by fine surface protrusions and depressions, which result from specific processing parameters, including an average power of 50 W, a frequency of 40 kHz, a depression depth of 20 nm, and a scanning speed of 500 mm s^−1^. The stainless-steel samples are manufactured for subsequent mechanical, tribological, and physical characterisation.

The surface texturing has been extensively employed in numerous engineering disciplines,^93–96^ particularly in tribological systems to mitigate friction and wear in contacting pairs.^97^ It has been shown that texturing results in a reduction in friction forces and lower friction coefficients, that are compared to those of smooth surfaces. Numerical predictions indicate that the optimal effect of surface texturing is achieved, when the texture depth is comparable to the minimum film thickness. However, the presence of hard burrs around dimples has a negative effect, leading to a higher friction coefficient. One method to reduce this negative effect is to increase the thickness of the shielding film. However, if the film layer in laser manufacturing is excessively thick, the indentation depth becomes insufficient, which consequently diminishes the favourable effect of texture. As a result, the COF will increase again. The optimal thickness of the masking oil film in the present laser process is 0.28 mm. It is also anticipated that corresponding mechanisms for improving surface texture quality will be identified, mainly attributed to the cooling effect of the oil film layer. This effect enhances the viscosity of the ablated melt and reduces the likelihood of molten material being expelled to the rim edge from the ablation zone. Furthermore, it is anticipated that the protective effect of the masking oil film on the materials within the laser irradiation spot, that will prevent the formation of hot oxides and prevent their adherence to the intact surface between dimples. Additionally, tribological tests have demonstrated that the textures obtained through the oil layer method have a beneficial impact on reducing the friction coefficient.^98^

A recent research report indicates that textured samples exhibit superior behavior in terms of friction coefficient compared to untextured samples, with reductions of up to 30% achievable regardless of whether the particle size exceeds the texture depth. Additionally, under the combined effect of texture size and density, the textures are capable of generating hydrostatic support forces,^99^ which reduce the contact pressure between friction pairs and thereby enhance lubricant retention capacity, as shown in Fig. 4b.^100^ When the particle size is smaller than the texture depth, the surface textures are capable of the retaining particles, thereby reducing the wear probability.^91^ Conversely, when the particle size exceeds the texture depth, some particles will be embedded in the textures, leading to impact wear. However, as the particle size increases, the effect of surface textures on reducing the friction coefficient will weaken.

The utilisation of periodic and symmetric 2D textures with diverse cross-sectional profiles has been employed to enhance and optimise surface physical responses, as exemplified by super-hydrophobicity.^101^ While the impact of texture height and spacing has been extensively investigated, the influence of texture shape has thus far been addressed only from a qualitative perspective. Accordingly, a polynomial framework is proposed to mathematically describe the cross-sectional profile of a texture and to provide the quantitative measurements for comparing the physical responses of the textured surfaces with varying shapes. The hydrodynamic friction response of a textured surface designed by the framework is tested under cylindrical Couette flow within a Taylor–Couette flow system. Both experimental and numerical results demonstrate that, compared with a smooth surface and a triangular texture, a texture with lower height and a half-distance equal to that of the concave surface exhibits lower torque, which helps the reduced torque.^102^

Surface texturing represents a specific form of surface engineering, whereby the dimples (oil pockets or cavities) are created on the sliding surface.^103^ Research has shown that the application of surface texturing effectively improves friction behavior under the boundary, mixed, and fluid lubrication conditions. Additionally, it has been shown to mitigate the tendency for abrasive wear and seizure.^104–106^ The stationary ring, composed of carbon graphite, interacts with the rotary ring, which is made of quartz. Oil pockets were created on the surfaces of the stationary rings, with a pit-area ratio of approximately 20% and varying dimple dimensions. The application of laser surface texturing can generate additional hydrodynamic pressure, enhance the load-carrying capacity of the lubricating film, and induce local cavitation to form vapor zones, thereby suppressing seal instability. Moreover, surface textures can store wear debris, increase the heat dissipation area, and reduce lubrication failure caused by overheating, thereby significantly improving the tribological performance of the sealing system.^107^ Ma *et al.*,^108^ based on the cavitation suction effect, processed counter-rotating helical grooves on the graphite surface, enabling the fluid on the low-pressure side to be drawn into the sealing chamber and thereby effectively preventing leakage. Concurrently, an integration of cavitation suction effect with a hydrodynamic pressure effect substantially improves the tribological performance of mechanical face seals in underwater environments.^92^ Furthermore, the orientation of the bottom texture exerts a more pronounced influence on the variation of the friction coefficient. When the bottom steps are aligned parallel to the arms of the chevron, the disc texture demonstrates enhanced stability and a reduced friction coefficient. The lowest friction coefficient is observed when the angle between the arms is 120°. At a 60° angle between the arms, the friction coefficient initially rises sharply, reaches a peak value after a few seconds, and then decreases at varying rates, as shown in Fig. 4c. The average friction coefficients for types 1 and 2 eventually stabilize at 0.32 and 0.29, respectively.

Surface texturing, in particular laser surface texturing (LST), is a universally applicable technique for modifying material properties in order to improve their tribological performance, corrosion resistance, and hydrophobicity. By enabling the precise fabrication of patterned surfaces with shape and depth control, LST can reduce friction and wear through mechanisms such as dynamic water pressure generation, lubricant retention, and particle retention. Textured surfaces generally have a lower friction coefficient than smooth surfaces. This is due to specially textured, sinusoidal depressions that generate additional hydrodynamic pressure, increase load capacity, and reduce the coefficient of friction (COF). In addition, the texture can store the lubricant, and create a hydrostatic reaction force that reduces contact pressure and allows for greater lubricant storage. In the case of friction and wear, if the particle size is smaller than the texture depth, the texture can trap the particles and reduce wear caused by them. Application of surface textures not only contributes to the reduction of friction and wear but also enhances the sealing performance of mechanical systems.

Surface texturing technology serves as a pivotal method for augmenting the wear resistance of materials. Nonetheless, a cohesive theoretical framework elucidating its mechanisms of friction reduction and anti-wear efficacy has yet to be established. The variability in outcomes is substantial, that is attributable to the multitude of variables inherent in texturing design and the spectrum of operational conditions. Consequently, the optimization of texturing morphology and parameter selection predominantly hinges on extensive test endeavors characterized by trial-and-error approaches. In the research domain of friction reduction and wear resistance enhancement, a plethora of outcomes has been documented, with some even contradicting each other. These discrepancies are largely attributed to the intricate interplay among of texturing parameters, operating conditions, the underlying mechanisms of action. Specifically:

First, there are the discrepancies regarding the influence of texture shape on performance. In different studies, the same texture shape exhibits opposite effects under varying scenarios. For example, Shinde *et al.*^109^ found that square-shaped textures had a lower friction coefficient than circular and triangular textures under oil lubrication conditions, while Qiu *et al.*^110^ concluded from the gas lubrication simulations that curved contour textures (*e.g.*, circular) offer better friction-reducing effects than straight-edged textures (*e.g.*, square). The core of this contradiction lies in differences in the dynamic pressure generation mechanism of textures caused by the lubricating medium (liquid *vs.* gas). In liquid lubrication, square textures offer better friction-reducing effects than straight-edged textures (*e.g.*, triangular).

Second, the optimal range of texture density varies considerably with application scenarios. Li *et al.*^111^ found that a 9.5% V-shaped texture density resulted in the lowest friction coefficient on the cemented carbide surfaces under oil lubrication. In contrast, Zou *et al.*^112^ suggested that a 2% density is optimal, with excessive density increasing wear. Zhang *et al.*^113^ reported that a 36% density reduced the wear rate by 38.6% under grease lubrication. This contradiction arises from the different dominant mechanisms of textures under varying lubrication conditions: oil lubrication relies on the oil storage and dynamic pressure effects of textures; low-density textures are more conducive for reducing liquid film rupture in the water lubrication; and grease lubrication requires higher density to store viscous lubricants.

Third, the effectiveness of multi-scale composite texture is controversial. Some studies propose that multi-scale textures (*e.g.*, combinations of large-sized and small-sized pits^112^) achieve better friction reduction through the synergistic effect of oil storage and dynamic pressure. But, Marian *et al.*^114^ observed that for uncoated samples under specific point contact conditions, the multi-scale textures did not significantly improve performance and even exacerbated wear, due to geometric complexity-induced stress concentration. The key controversy lies in the difficulty of parameter matching for multi-scale textures-improper design of spacing and depth across different texture scales disrupts the continuity of the lubricating film, thereby weakening the overall effect.

Fourth, no consensus has been reached on the performance comparison between symmetrical and asymmetrical textures. Shen *et al.*^115^ noted that asymmetrical textures (*e.g.*, V-shaped with a flat front end) exhibited better load-bearing capacity in unidirectional sliding. Nevertheless, Tewelde *et al.*^116^ found that under dry friction conditions, the friction coefficient of asymmetrical textures can differ by 28.1% depending on the sliding direction, with stability inferior to that of symmetrical textures. Tang *et al.*^117^ also provided a summary of these phenomena.

A contradiction reflects the coexistence of directional advantages of asymmetrical textures and their poor adaptability to working conditions: they are more suitable for unidirectional motion scenarios (*e.g.*, bearings) but may exacerbate friction fluctuations in reciprocating motion scenarios (*e.g.*, piston rings). These controversies essentially stem from the multi-source nature of surface texture action mechanisms. The same texture parameters may simultaneously involve mechanisms such as hydrodynamic lubrication, wear debris storage, and friction film formation under different lubrication states, contact modes (sliding/rolling), and the medium types. Current research cannot quantify the contribution ratio of individual mechanisms, making unified conclusions difficult. In the future, it will be necessary to combine multi-physics simulations and cross-scenario experiments to establish a “texture parameter-working condition-dominant mechanism” correlation model, thus reducing controversies. Table 2 summarizes the shapes, geometric dimensions, texture densities, and spacings of the aforementioned different surface textures, their corresponding tribological behaviors and friction-reducing and anti-wear mechanisms.

**Table 2:** Information on different surface texturing and their corresponding tribological behaviors (*h* (depth), *d* (diameter), *w* (width))

Means	Profile shape	Geometrical parameter	Texture density (%) or spacing	Sliding type	Lubrication type	Tribology performance	Friction reduction mechanism	Ref.
Two-photon lithography and atomic layer deposition	Hemisphere	*d* = 200 nm (10 mW), 300 nm (12.5 mW), 400 nm (15 mW)	1 μm	Unidirectional linear sliding	Dry friction	Under a load of 8000 μN, the COF is approximately 0.070, which is 35% lower than that of bare glass substrates	Reduce the contact area enhance structural stiffness	82
Laser pulse ablation	Regular slot shape circle	*w* = 300 μm, 500 μm, 600 μm, *h* = 10–15 μm, *d* = 10 μm	10, 20, 40	Rotary ball-disc sliding	Turbine L-TSA46 oil	The COF is 0.03, which is approximately 75% to 80% lower than that of a textured surface	Enhance hydrodynamic pressure establish a fluid lubrication film store lubricating oil capture grinding swarf	97
Nanosecond laser	Doughnuts or volcanoes	*d* = 400 μm	5	Reciprocating sliding	Medicinal white oil (Caltex, ISO VG 32)	The textured surface prepared by the oil layer method (with an oil film thickness of 0.28 mm) has the lowest COF	Reduce contact stress avoid the ploughing effect store lubricating oil reduce the contact area and the formation of hard burrs	98
Laser surface texturing	Circular or hemispherical	*h* = 10 μm, 40 μm, *d* = 2000 μm, 1000 μm	20	Translational slide	Water	The friction torque is reduced and remains stable at 2400 rpm	Cavitation effect reduces the contact area maintain the stability of the liquid film lubricating film capture grinding swarf	107
Laser	Chevron	*d* = 32 μm	5.05, 9.5, 13.02, 15.2	Unidirectional sliding	Liquid lubricant	The surface with 9.5% texture density shows the lowest friction coefficient	Increase fluid dynamic pressure	118
Laser	Chevron	*d* = 3 μm	9.1, 11.5	Reciprocating sliding	Oil	The surface with a texture density of 11.5% has the lowest friction coefficient	Increase the thickness of the lubricating film	119
Milling	Linear groove	*d* = 400 μm, *w* = 1.5 μm	34	Unidirectional sliding	Lubricating oil contains sands	The wear resistance is 4.9 times that of non-textured surfaces	Store silica sand	120

#### 2.1.3 Surface hardening

Surface hardening is a technique in which a metallic material is generally heated or mechanically processed to increase its surface hardness, thereby enhancing the resistance to wear and fatigue. Traditional surface hardening methods, such as carburizing, nitriding, and boriding, involve the diffusion of elements into the component surface and generally require high-temperatures. These methods typically result in a significant increase in the thickness of the hardened layer and induce a phase change in the underlying microstructure, leading to improved material hardness. The core differences among the three processes are as follows: carburizing involves the dissolution and diffusion of carbon into the base phase, nitriding primarily enhances material strength through the precipitation of hard compounds, whereas the boronizing achieves a surface hardening by forming the layer of high-hardness compounds. More recent surface hardening techniques, such as plasma surface diffusion,^121^ dual-phase quenching,^122^ laser surface alloying,^123^ microwave heating,^124^ friction stir processing,^125^ and high-current pulsed electron beam processing^126^ demonstrate the promising capabilities for generating refined microstructures or additional alloying elements on the surface. Specifically, the fore-said methods achieve the compositional or microstructural modification through “diffusion/phase transformation/alloying”; the middle two focus on “efficient heating assistance” or “solid-state deformation refinement”; and the last one relies on “high-energy beam instantaneous thermal effect” to complete surface reconstruction.

**Fig. 5:**
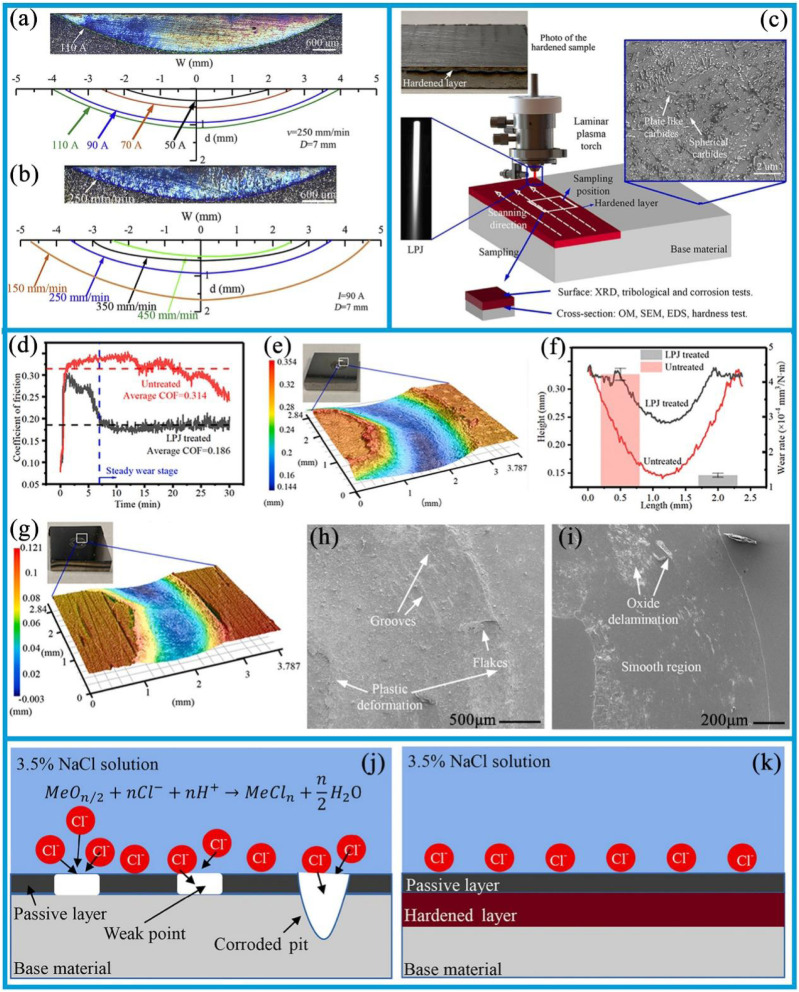
(a) Optical image of the LPJ hardened layer (*I* = 110 A) and boundary profile of the hardened layer after LPJ surface hardened under different arc currents (*v* = 250 mm min^−1^, *D* = 7 mm). (b) Optical image of the LPJ hardened layer (*v* = 250 mm min^−1^); the boundary profile of the hardened layer after surface hardening of LPJ at different scanning speeds (*I* = 90 A, *D* = 7 mm). Reproduced from ref. 127 with permission from Elsevier, copyright 2020. (c) Schematic diagram of LPJ surface hardening experiment. (d) Curve of the instantaneous friction coefficient of the sample. (e and f) Three-dimensional surface image of the specimen after friction and wear. (g) Depth profile curve and wear rate histogram of the sample wear trajectory. (h and i) The 100× magnification of the worn surfaces of untreated samples and samples treated with LPJ. (j and k) Schematic diagram of surface corrosion mechanism of untreated and LPJ-treated samples. Reproduced from ref. 128 with permission from Elsevier, copyright 2021.

An extensive research has been dedicated to the surface hardening in structural applications. The laminar plasma jet (LPJ), which is distinguished by its relatively lower cost, higher process efficiency, and high heat flux approaching that of a laser, is a relatively novel heat source.^127,129–132^ Over the past few decades, LPJ has been extensively employed for surface hardening purposes. The anti-wear effect of surface hardening treatment is influenced by arc current and scanning speed during application. As shown in Fig. 5a, increasing the arc current from 50 A to 110 A notably enhanced both the maximum depth and width of the hardened layer, with the depths increasing from 0.3 mm to 1.1 mm and the widths from 4.0 mm to 7.8 mm, respectively. Furthermore, as depicted in Fig. 5b, increasing the scanning speed from 150 mm min^−1^ to 450 mm min^−1^ led to a notable expansion of the hardened layer, with the width increasing from 4.9 mm to 9.4 mm and maximum depth growing from 0.4 mm to 1.7 mm. These observations suggest that the geometry of the hardened layer can be tailored by adjusting the arc current and scanning speed. Furthermore, the degree of surface hardening achieved is dependent on the specific surface hardening treatment employed.^127^ Pan *et al.*^133^ applied the non-transferred direct current (DC) laminar plasma jet (LPJ) to treat the surface of W–Mo–Cu cast iron, and found that the material hardness increased by a factor of two to three following LPJ surface hardening. Petrov *et al.*^134^ and Kanaev *et al.*^135^ have proposed that plasma surface hardening is an effective method for enhancing the wear resistance of wheel-set ridges and wheel-pair rims. Xu *et al.*^132^ investigated the effects of plasma discrete quenching on the surface properties of rail steel. The findings indicated that the wear loss of quenched workpiece was reduced by 45% compared to the untreated control. The surface hardening test is shown in Fig. 5c; the underlying mechanism involves the rapid cooling rate of the LPJ treatment inducing the microstructural transformation in P20 mold steel from tempered martensite to dense lath martensite. The formation of a hardened layer composed of lath martensite leads to an increase in hardness from 300 HV to approximately 600 HV. The enhancement in surface hardness contributes to enhanced wear resistance of P20 mold steel. As shown in Fig. 5d–g, following the LPJ treatment, the metal surface transitioned into a stable wear stage after the initial running-in stage. The wear trace width reduced from 2.3 mm to 1.3 mm, the wear depth from 0.182 mm to 0.013 mm, and the wear rate decreased from 0.323 × 10^−4^ mm^3^ (N^−1^ m^−1^) to 0.141 × 10^−4^ mm^3^ (N^−1^ m^−1^). As shown in Fig. 5h and i, the wear mechanism shifted from a combination of abrasive and adhesive wear to mild oxidative wear after a LPJ treatment. Furthermore, the corrosion resistance of surfaces subjected to LPJ treatment was significantly improved.^128^ In contrast, without LPJ treatment, the passive layer lacks sufficient density; as the chemical reaction progress, the passivation layer is vulnerable to damage, thereby exposing the substrate. This results in the formation of numerous corrosion pits and gaps on the surface (Fig. 5j). In contrast, after LPJ treatment, a large number of spherical carbides dissolve and capture Cr atoms, which are uniformly distributed, thereby forming a passivation layer (Fig. 5k). This effectively reduces the susceptibility to intergranular corrosion and thus enhances the overall corrosion resistance.

Innovative design strategies, such as surface engineering (coating, texturing, curing, and structuring) and matrix reinforcement (composition, microstructure, and reinforcement), can improve the wear resistance of materials. Surface engineering techniques, including coating, surface texturing, and surface hardening, achieve wear resistance improvement through the structural modifications. In contrast, the matrix reinforcement involves altering the material's composition and microstructure, as well as introducing reinforcing phases into the material matrix, to enhance both mechanical properties and wear resistance. The enhancement of basic mechanical properties, such as hardness, stiffness, strength, and plasticity, plays a critical role in determining a material's wear resistance. Wear-resistant materials are used in the various industries, including aerospace, automotive, wind turbines, the micro-/nano-electromechanical systems, atomic force microscopy, biomedical devices. Surface hardening techniques such as the plasma surface diffusion, dual-phase quenching, laser surface alloying, microwave heating, friction stir processing, and high-current pulsed electron beam processing are regarded as emerging and promising techniques. These methods offer significant potential for the precise microstructural control and the incorporation of additional surface alloying elements.

#### 2.1.4 Architecture

The heterogeneous and hierarchical nature of wear arises from the concurrent operation of multiple wear mechanisms across different scales and hierarchical levels. This subsection focuses on the design of hierarchical and heterogeneous surface structures for reducing friction and wear.

The interlaminar shear and abrasive wear properties of composites fabricated using polytetrafluoroethylene (PTFE) coated glass fibre fabrics^136–139^ can be enhanced through hybridisation. Hybrid composites were manufactured using the same fabrics, with a steel metal mesh (MM) placed on both the top and bottom surfaces of the laminates. The incorporation of MM was observed to affect the physical properties of the composites, including thickness, density, and void content. The addition of MM into hybridised homogeneous composites resulted in an increase in interlaminar shear strength (ILSS) by 56%, 10%, and 18% for 2D, 3D, and SW composites, respectively. In terms of wear performance, hybrid composites exhibited superior performance with respect to the coefficient of friction (COF) and specific wear rate (*K*_0_). The SW and SW-MM composites demonstrated superior wear performances, when compared to the 2D, 2D-MM, 3D, and 3D-MM composites. Scanning electron microscopy (SEM) analysis revealed that the interfacial bonding between the fibre and matrix was enhanced in the 2D and 2D-MM composites.^140^

**Fig. 6:**
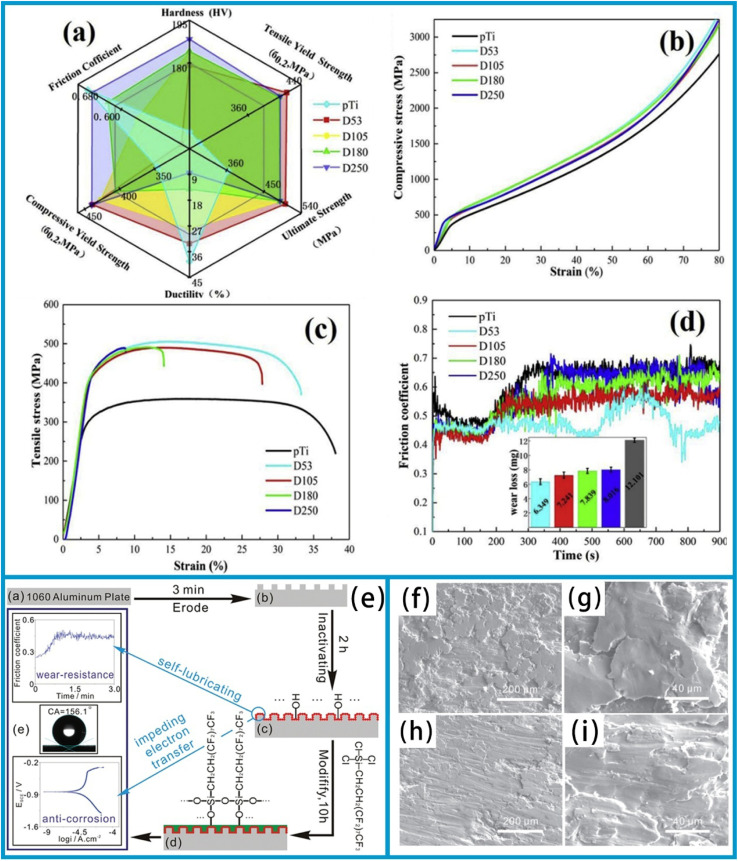
The mechanical and wear behaviour of D53-250 Ti/NDs nanocomposites and pure Ti. (a) The hardness, tensile yield strength, ultimate strength, ductility, compressive yield strength, and coefficient of friction of the composite material are displayed in the form of a radar chart. (b) Tensile stress–strain curves and (c) compressive stress–strain curves. (d) The coefficient of friction curves and insertion wear weight loss of samples with different network sizes. Reproduced from ref. 141 with permission from Elsevier, copyright 2020. Schematic process of preparing micron- and nano-scale binary hierarchical structures (e) Scanning electron microscope images of wear traces. (f) For the treated surface and (h) for the untreated surface. (g) and (i) The magnified scanning electron microscope images of the wear traces in f and h, respectively. Reproduced from ref. 142 with permission from American Scientific Publishers, copyright 2016.

The incorporation of metallic meshes enhances the material's wear resistance, and performance differences arising from varying mesh sizes are a significant issue that warrants further investigation. Previous studies have ensured minimal differences among various mesh sizes. However, it has been observed that the material's ductility increases as the network size decreases (from 8% to 33%), and its wear resistance also improves with decreasing mesh size.^141^ This phenomenon can be attributed to a significant increase in the microhardness of composites, microhardness, which stems from the incorporation of metallic mesh, the presence of residual ND reinforcement, and solid solution strengthening by C atoms. As shown in Fig. 6a, the microhardness of D250 reaches 188 HV, a notable increase of 20.5%. The compressive yield strength showed minimal variation with increasing mesh size, with no clear or consistent trend observed (Fig. 6b). In contrast, the yield strength and ultimate tensile strength of the composites were enhanced, reaching 286 MPa and 364 MPa, respectively. However, this enhancement was accompanied by a reduction in elongation strain. Furthermore, the nanocomposites' tensile strength showed the minimal variation as the mesh sizes decreased, while ductility increased notably. In contrast, the ductility of composites with large mesh sizes decreased, as shown in Fig. 6c. This reduction is primarily due to an increase in the proportion of local reinforcing phases.

In the case of nanocomposites, reinforcing particles surrounding the matrix will gradually form a “barrier wall”. This phenomenon may deteriorate the matrix's connectivity and reduce the composite's overall plasticity. Consequently, a uniform dispersion of the reinforcement facilitates superior connectivity between the matrix and the reinforcement, as well as stronger interfacial bonding. Furthermore, a smaller grain size can act as a fine-grain reinforcement, thereby enhancing the composites' wear resistance (Fig. 6d). The combined effect of the quasi-continuous mesh structure, reinforcing particles, and the reinforcement phase is the primary factor contributing to the composites' exceptional wear resistance.

In recent years, a number of novel hierarchical structures have been fabricated to achieve the significant improvements in adhesion and tribology. Wang *et al.*^143^ employed first-principles calculations to analyze the nanoscale tribological behavior of fluorographene/fluorographene, MoS_2_/MoS_2_, and the fluorographene/MoS_2_ heterostructures. An interlayer shear strength of fluorographene/MoS_2_ heterostructure was measured at 2.9 MPa, approximately two orders of magnitude lower than that of the fluorographene/fluorographene (136 MPa) and MoS_2_/MoS_2_ (470 MPa) bilayers. This discrepancy can be attributed to intrinsic lattice mismatch and the formation of periodic Moiré patterns, which together result in an extremely low energy barrier during sliding. Li *et al.*^142^ fabricated a micro-nano binary hierarchical structure comprising a stepped microstructure formed through etching of the aluminum surface, along with a layer of nanoparticles deposited thereon; the preparation process is illustrated in Fig. 6e. Wear test results revealed that the wear rate of the untreated aluminum surface was 1.49 × 10^−5^ mm^2^ N^−1^, whereas that of the treated hierarchical structure surface was reduced to 0.89 × 10^−5^ mm^2^ N^−1^, thus indicating a substantial enhancement in wear resistance. This enhancement in performance can be attributed to the several contributing factors: firstly, the uniformly distributed nano-particles function as the self-lubricating agents, effectively reducing friction. Secondly, the modified surface, which features a hierarchical microstructure, and tends to undergo flaking during the wear process. This flaking promotes the formation of a protective friction layer on the surface, thereby minimizing a direct wear of the underlying substrate. Additionally, the contact area between the treated aluminum surface and the steel ball is reduced, if compared to that of the untreated surface, which further contributes to its improved tribological performance. Examination of the wear marks, as shown in Fig. 6f, reveals that those on the treated surfaces are distinct and discontinuous, whereas those on the untreated surface are continuous. Fig. 6g and i present the scanning electron microscope (SEM) magnified views of the wear marks shown in Fig. 6f and h, respectively. As illustrated in Fig. 6g, the wear marks on the treated surface are localized and limited in extent. In summary, the fabrication of a hierarchical stepped microstructure on the aluminum alloy surfaces can markedly improve their wear resistance.

Similarly, in the field of dentistry, the friction and wear performances of materials are crucial. Ceramic-polymer composite materials, modeled after biological processes and possessing a pearl-layered structure and physical composition similar to that of human teeth, have been the subject of quantitative investigation. Their specific wear mechanisms result in varying levels of wear on opposing enamel surfaces, which are influenced by the material's structural configuration and orientation. Notably, when compared to layered structures, the physical structures of these composites exhibit the reduced wear. Moreover, unlike smooth ceramics, these materials do not accelerate enamel wear, as their high ceramic content keeps a relatively smooth enamel contact interface. Coupled with mechanical properties such as stiffness and hardness that closely match those of human enamel, along with excellent fracture toughness and machinability; these composites exhibit considerable potential for dental applications.

**Fig. 7:**
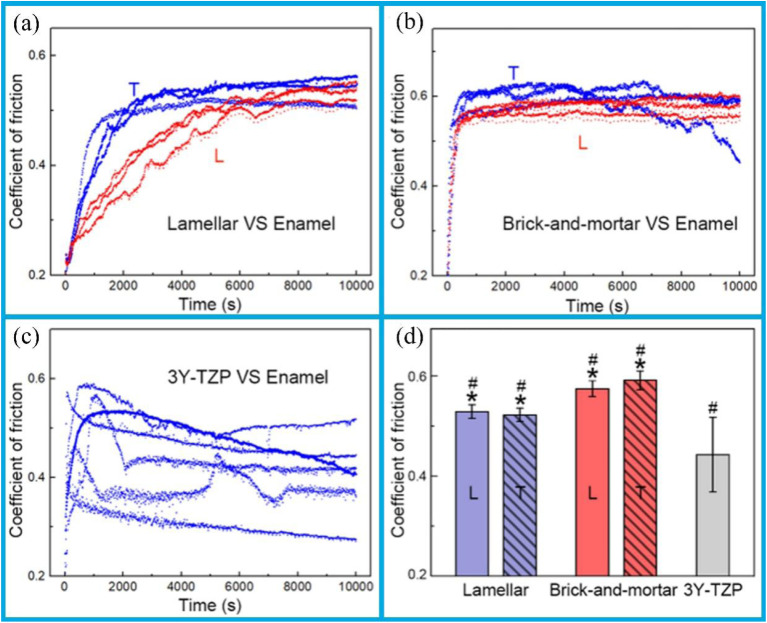
The following plots illustrate the instantaneous coefficient of friction (COF) of ceramic-polymer composites when worn by human enamel along different orientations: (a) pearly laminates; (b) brickwork structure; (c) comparison of corresponding data for 3Y-TZP; (d) comparison of the average COFs of the groups, excluding the initial growth phase. In the case of the laminated and brickwork structures, a statistically significant difference is indicated by an asterisk, while a hashtag indicates a statistically significant difference between bio-inspired composites and 3Y-TZP. Reproduced from ref. 144 with permission from Elsevier, copyright 2022.

Bionic ceramic-polymer composites feature pearly lamellar structures and solid structures morphologies, with mechanical properties such as stiffness and hardness similar to those of human teeth. These materials exhibit distinct wear mechanisms that result in varying levels of enamel wear, depending on the specific structural type and orientation. The polymer phase is particularly susceptible to wear, which leads to fragmentation of the ceramic lamellae and the exfoliation of debris from the lamellar structure. This process contributes to an increase of surface roughness of the contact interface during frictional interaction.^144^ Hence, lamellar-structured composites undergo a pronounced wear phase in which the coefficients of friction (COFs) increase over time before reaching a stable plateau (Fig. 7a). In contrast, the COFs of solid structured composites quickly reach a stable plateau phase after an initial sharp increase (Fig. 7b). During friction, changes in contact surface conditions caused by the wear of the enamel against the opposing materials, and resulted in COFs that are highly variable in both magnitude and trends across samples, with no clear stable plateau phase observed (Fig. 7c). In sliding contact with human enamel, the bionic composites with pearly lamellae and solid structures exhibit higher COFs than those of 3Y-TZP. However, due to the low polymer phase content in solid structures, the brickwork structure consistently exhibits higher COFs than the lamellar structure (Fig. 7d). This result can be attributed to the polymer phase acting as a lubricant during friction: insufficient polymer phase content fails to provide an adequate lubrication during the frictional wear process.

Hierarchical and heterogeneous structures hold great potential for enhancing the wear resistance of materials. Hybrid composite materials, such as metal mesh-reinforced PTFE-coated glass fiber fabrics, exhibit significantly improved interlayer shear strength and wear resistance, thanks to the enhanced interface bonding and reinforcement effects. At the nanoscale, the modifications such as these additions of nanodiamonds to the titanium matrix and the control of metal dimensions lead to considerable improvements in microhardness and mechanical strength. Although ductility is slightly reduced, the wear resistance is substantially enhanced. In addition, novel layered structures inspired by biological systems, such as fluorographene/MoS_2_ heterostructures and aluminum surfaces with micro-nano scale structures, exhibit the improved adhesion and tribological properties. Finally, biomimetic ceramic-polymer composites with pearl-like and solid structures show promise in dental applications, as they possess low friction and wear characteristics and favorable mechanical properties similar to those of human teeth.

### 2.2 Matrix strengthening

Significant efforts have been devoted to the development of bulk materials exhibiting enhanced wear resistance and improved anti-friction properties. To date, several strategies have been established, primarily focusing on compositional control, microstructure design, and the incorporation of reinforcing phases. This subsection reviews recent studies on matrix strengthening, with particular attention to its effect on tribological performance.

#### 2.2.1 Compositions

**Fig. 8:**
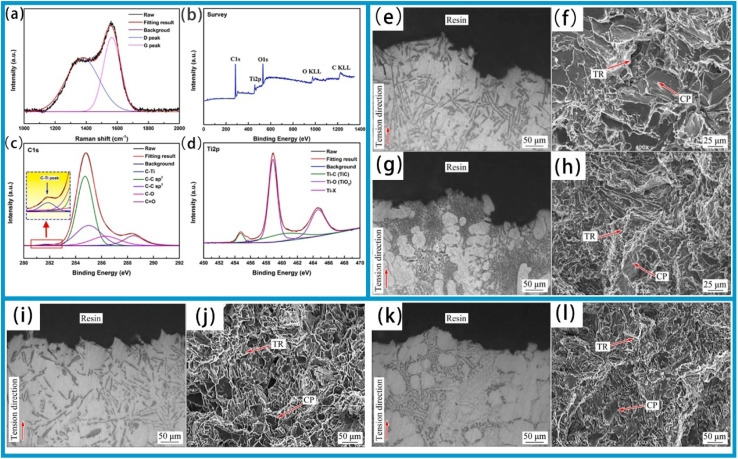
Raman and XPS results of the multilayer Ti-DLC coating surface: (a) Raman spectrum; (b) survey; (c) C1s; and (d) Ti_2_p. Reproduced from ref. 145 with permission from Elsevier, copyright 2021. Tensile fracture surface images of as-cast alloys: (e and f) Al–12Si–3Cu–0.5Mg; (g and h) Al–12Si–3Cu–2Mg. Tensile fracture surfaces of T6-treated alloys: (i and j) Al–12Si–3Cu–0.5Mg; (k and l) Al–12Si–3Cu–2Mg (CP: cleavage plane; TR: tear ridge). Reproduced from ref. 146 with permission from Elsevier, copyright 2021.

Advancements in the science and technology, together with the expansion of the manufacturing industry, have driven the development of mechanical components towards more lightweight, miniaturized, and intelligent designs. An evolution imposes greater demands on the reliability and stability of mechanical components. Among these requirements, the anti-wear and friction reduction properties of materials play a direct role in determining the operational stability and service life of mechanical components. Consequently, enhancing these properties have become a focal point in materials research. Relevant studies indicate that the multilayer structure, along with interface and grain boundary effects generated by metal element doping, enables the metal coatings and metal-based self-lubricating materials, thus achieving excellent comprehensive performance. Cao *et al.*^145^ enhanced the hardness of DLC coating by introducing the Ti element. Raman spectroscopy was used to analyze the bonding structure of carbon atoms on the coating surface, which was categorized into the G peak (“graphite”) and D peak (“disordered”). Fig. 8a shows the Raman spectrum of the carbon atom bonding structure on the coating surface. The D peak, corresponding to a disordered structure, primarily originates from the symmetric breathing modes of sp^2^ atoms in aromatic rings. In contrast, the G peak represents the lamellar cluster structure, attributes to the stretching vibrations of sp^2^ bonds in both aromatic rings and chains. Furthermore, XPS measurement of the coating surface (Fig. 8b) reveals the presence of C1s, Ti2p, and O1s peaks in the samples. During the coating preparation process, the O1s peak may result from either impurities introduction or oxygen adsorption on the coating surface after exposure to air before analysis. As shown in Fig. 8c, a distinct C1s peak is observed at approximately 284.7 eV, which indicates a typical DLC structure of the coating. Doped Ti atoms interact with C atoms to form nanocrystalline TiC on the coating surface; however, the fitting results of the Ti2p spectrum using the Lorentz–Gaussian function (Fig. 8d) indicate that only a limited number of C atoms are involved in the formation of TiC crystals. This suggests that only a small fraction of Ti atoms in the coating exists in the form of nanocrystalline TiC. A incorporation of metal atom into amorphous carbon to generate nanocrystalline carbides can enhance the mechanical properties of composite coatings. Additionally, such nanocrystalline carbides contribute to enhanced high-temperature tribological properties, thus improving a wear resistance of coating under elevated temperatures.

In a study by M. Beder *et al.*,^146^ the effects of magnesium addition and T6 heat treatment on the microstructures, mechanical properties, and tribological behaviors of Al–Si–Cu–Mg alloys were systematically investigated. Fig. 8e–l shows the tensile fracture characteristics of as-cast and T6-treated alloys. During the loading process, stress concentration occurs at the interface between hard particles (containing Si, Mg, and Cu phases) and the α(Al) matrix due to the differences in their mechanical properties. When the applied stress exceeds either the interfacial bonding strength or the intrinsic strength of the particles, particle fracture or debonding from the matrix may occur, thereby acting as initiation sites for microcracks. As illustrated in Fig. 8e, g, i and k, cracks propagate along hard particle-rich regions surrounding the α(Al) phase, ultimately resulting in tensile fracture. Fig. 8f, h, j and l reveal that the fracture surface comprises cleavage planes (CP) and tear ridges (TR). The cleavage planes are associated with the fracture or peeling of hard particles, whereas the tear ridges represent the fracture surfaces of the α(Al) phase along the tensile direction. This microstructural observation directly supports the conclusion that cracks originate from the fracture or detachment of hard particles. T6 treatment leads to an increase in the number of tear ridges and a reduction in the width of cleavage planes on the alloy fracture surface (Fig. 8j and l). This phenomenon can be attributed to the heat treatment's ability to spheroidize Si particles, refine β-Mg2Si and *θ* phases, and then distribute them more uniformly, thereby mitigating the stress concentration effect. However, the π and Q phases, which remain morphologically unchanged during the process, continue to act as the potential crack initiation sites, thereby limiting the enhancement of the alloy's mechanical properties after T6 treatment. With increasing Mg content, Si particles are further refined, the α(Al) phase grain size increases, the cleavage plane area diminishes, and the number of tear ridges rises. Nevertheless, excessive Mg addition (up to 2.5 wt%) may compromise the alloy's strength and toughness due to the inherent brittleness of β, π, and Q phases. Overall, T6 treatment significantly improves both mechanical properties and wear resistance by promoting the spheroidization of eutectic Si particles, eliminating the Chinese-character-shaped morphology of β-Mg2Si, and facilitating the formation of finer *θ*-CuAl2 and β-Mg2Si precipitates. Tang *et al.* analyzed the characteristics of ceramic wear-resistant phases from the perspectives of bonding properties and electronic structure. For the bonding properties, their findings indicate that the mechanical properties of ceramic materials are governed by the synergistic effects of multiple bonding forces, including metallic, covalent and ionic bonding.^147^ For example, through *in situ* atomic force microscopy (AFM) analysis combined with first-principles calculations, they revealed that the strength of M7C3 carbides is predominantly governed by their metallic bonding components. By partially substituting metallic elements within the carbides with elements exhibiting a higher electron work function (EWF), these metallic bonding components can be modulated, thus leading to an improvement in the mechanical properties of the carbides. From the perspective of electronic structure analysis, they demonstrate that the mechanical properties of M7C3 carbides are strongly correlated with their electronic behavior. This relationship arises from the fact that the Young's modulus of these carbides is predominantly determined by their metallic bonding components, which are closely linked to the electron work function (EWF). Consequently, it can be reasonably inferred that the incorporation of metals with higher electron transfer efficiency—such as titanium, which exhibits a higher EWF—into these carbides can reinforce their metallic bonds, thereby enhancing their mechanical properties and ultimately improving the wear resistance of the coatings.^148,149^ Additionally, they identified an electronic competition effect among of the elements. For example, when carbon (C) and silicon (Si) coexist, carbon—which possesses a higher electronegativity (2.55) compared to silicon (1.90)—exhibits a greater tendency to attract electrons from iron (Fe). This interaction weakens the Fe–Si covalent bonds and consequently reduces the wear resistance of Fe_1_Mn_0.3_Si alloys containing carbon. Furthermore, their findings reveal that elemental bond strength is negatively correlated with the wear rate. For instance, silicon, which has a high bond order, effectively reduces the wear rate, whereas manganese—when adds individually and exhibiting a lower bond order relative to the overall metallic bonding—may lead to an increase in the wear rate.^150^

In addition to the aforementioned doping strategy, extensive research has been conducted on the effects of modifying the proportion of constituent elements on the matrix wear resistance. Adding an appropriate amount of Si to Zn–Al based alloys has been shown to enhance their hardness, strength, and thermal stability.^151^ However, due to the limited solubility of Si in the Zn–Al matrix, the introduction of Si into the alloy leads to the formation of irregularly shaped Si particles in the microstructure. MDF exerts a significant influence on the as-cast microstructures. The large shear strains applied during this process lead to the breakdown of the initial dendritic network in the samples. This refinement improves the distribution of Si in the matrix and substantially reduces the presence of existing micropores. Similarly, it has been demonstrated that the simultaneous alloying of TiCN coatings with Nb and Ag can effectively address the primary limitations of TiCN, namely its relatively high friction coefficient and poor oxidation resistance at elevated temperatures.^152^ To enhance tribological properties over a wide temperature range (25–700 °C), a relatively high Ag content (15%) is necessary. Furthermore, the prepared silver-doped coatings provide the active oxidation protection and self-healing capabilities, attribute to the migration of silver particles to damage areas, such as cracks, pinholes, or oxidation spots.

Multilayer structures and metal element doping have been proven effective in the enhancing material properties. A integration of different materials within a multilayer architecture enables the synergistic utilization of their characteristics, thus improving overall performance. The incorporation of metallic elements into amorphous carbon coatings, such as TiCN, can significantly enhance their mechanical and tribological properties. Additionally, modifications to alloy compositions can influence the wear resistance. For example, the addition of Si to Zn–Al alloys and the doping of Nb/Ag into TiCN coatings can improve material hardness, strength, and oxidation resistance. The processing techniques, such as MDF and heat treatment, play a crucial role in microstructural refinement and further enhancement of material properties.

#### 2.2.2 Microstructures

Similar to the hierarchical or heterogeneous structure designs discussed in Subsection 2.1.4, the matrix microstructure plays a crucial role in determining the material's tribological properties. These properties are strongly influenced by the factors such as grain size, precipitates (or second phases), grain boundary structure, dislocation density, and the presence of twins.

**Fig. 9:**
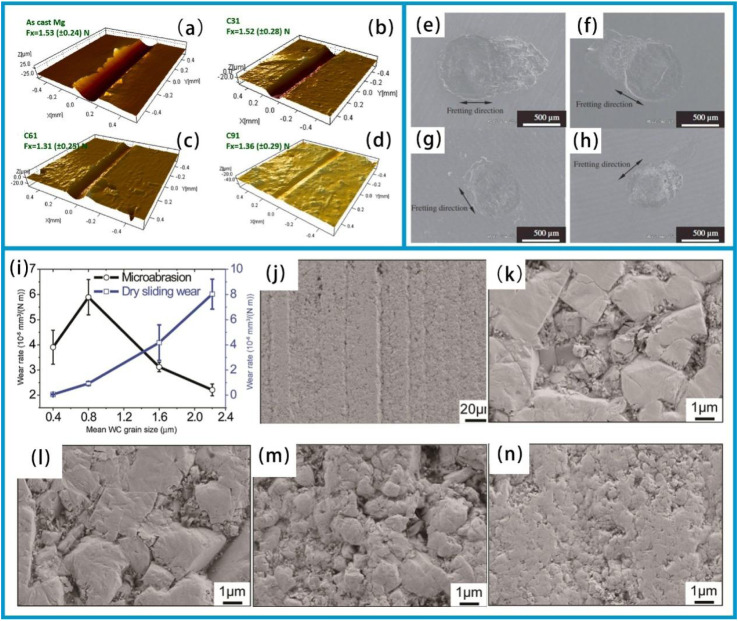
Three-dimensional micrograph of scratched surfaces: (a) As-cast Mg; (b) C31; (c) C61; (d) C91 MMC. Reproduced from ref. 153 with permission from Elsevier, copyright 2020. Scanning electron microscope (SEM) images of wear marks resulting from friction wear experiments on untreated (e) CP-Ti, (f) Ti6Al4V alloys, (g) UNSM-treated CP-Ti, and (h) Ti6Al4V alloys. Reproduced from ref. 154 with permission from Elsevier, copyright 2021. (i) The wear rates of WC-Co with different grain sizes are presented. SEM images show the surface morphologies of WC-Co with different grain sizes: (j and k) 2.2 μm; (l) 1.6 μm; (m) 0.8 μm, and (n) 0.4 μm after micro-wear tests under a contact load of 0.2 N. Reproduced from ref. 155 with permission from Elsevier, copyright 2019.

The *in situ* generation of ceramic reinforcements during the fabrication of metal matrix composites (MMCs) provides the multiple advantages, including excellent dispersion, the formation of nanoceramics, and favorable interfacial compatibility with the matrix. Relevant studies have demonstrated that Mg-based hybrid metal matrix composites (hMMC) can be synthesized through the *in situ* formation of the ceramic reinforcements within the Mg matrix.^153^ As shown in Fig. 9a–d, composite solids with different ceramic reinforcement contents (C31 = 3 wt%, C61 = 6 wt%, and C91 = 9 wt%) exhibit distinct hardness and wear resistance properties. Compared with the extensive and deep surface abrasion marks observed in cast Mg (Fig. 9a), the surface abrasion marks of the C91 MMC are much shallower and narrower (Fig. 9d). This indicates that the C91 MMC possesses higher hardness but lower ductility. This phenomenon arises because the *in situ* chemical reaction between ceric ammonium nitrate (CAN) and the Mg melt generates nano-to micrometer-sized CeO_2_ and MgO particles, which are uniformly distributed throughout a microstructure. Additionally, the good presence of cauliflower-like structures and nanopores in the microstructure contributes to this effect. These microstructure features collectively enhance the wear resistance of the composites. Furthermore, it has been reported that a nanosurface layer can be formed on a material surface using ultrasonic nanocrystalline surface modification (UNSM) technology.^154^ These nanostructures, which consist of ultrafine lamellar particles and are characterized by the combined effects of precipitated phases and high dislocation densities, exhibit the excellent mechanical properties. From a chemical standpoint, during UNSM processing, titanium elements on the material surface react with oxygen to form a denser oxide layer. This layer not only enhances the material's chemical stability but also reduces chemical wear during wear. Additionally, the formation of a nanocrystalline structure significantly increases the surface hardness, further reducing material adhesion during friction, thereby markedly lowering the friction coefficient.

In the context of frictional wear, the micromotion wear tracks of UNSM-treated samples (Fig. 9g and h) exhibited a shallower depth and smaller diameter (520 μm, 480 μm) compared to those of the untreated samples (Fig. 9e and f) (680 μm, 630 μm). The nano-microstructured surface layer significantly enhanced the material's resistance to micromotion damage, and then improved its tribological properties.

In addition to grain refinement achieved through surface modification, grains can also be refined to ultra-fine grains (UFG) with nanoscale second phases and high-angle grain boundaries through severe plastic deformation processes such as equal channel angular pressing (ECAP) and high-pressure torsion (HPT). It has been observed that as the indentation size decreases, the fracture mode transitions from tensile to shear dominance, accompanied by an increase in tensile strength.^156^ Surface modification and the application of external forces can enhance the mechanical properties and wear resistance of composites. However, it is important to note that temperature also plays a crucial role in affecting materials' wear resistance. Research findings indicate that the friction coefficient consistently decreases with increasing temperature and silicon content. This demonstrates that silicon-doped composites exhibit excellent tribological properties.^157^ Specifically, at 800 °C, wear resistance is enhanced by approximately 17 times, attributed to the improved mechanical properties and the *in situ* formation of a nano-oxide glaze layer on the worn surface.

Hot rolling has been observed to modify the microstructure of TiNi alloys, resulting in a notable improvement in their tribological performance. This phenomenon is attributed to their self-adaptive behavior. Wang *et al.*^158^ demonstrated that the superior pseudoelasticity and transformation capacity of TiNi shape memory alloys (SMAs) contribute to their wear resistance. Specifically, alloys with fine grain sizes are less prone to adhesion on their wear surfaces compared to those with coarse grains. In contrast, in dry sliding wear and micro-wear tests, the effect of grain size on the wear resistance of cemented carbides is completely opposite, as shown in Fig. 9i.^155^ In dry slide wear, reducing the WC grain size increases hardness, which significantly decreases the wear rate. Concurrently, the occurrence of plastic deformation, fracture, fragmentation, and oxidation also decreases. However, in micro-wear, the primary wear mechanism involves the pull-out of WC grains after the removal of the cobalt phase. Thus, the hardness of cemented carbides is not a key factor, as shown in Fig. 9j–n. Therefore, although the wear resistance of cemented carbides generally decreases with decreasing grain size, a slight improvement is observed in ultrafine-grained cemented carbides.

Strain hardening is critical to wear resistance, as precipitates or second phases impede dislocations motion. The underlying mechanism is discussed in the following subsection.

#### 2.2.3 Reinforcements

The intentional incorporation of reinforcements, including precipitates and second-phase particles, has garnered significant attention in the development of wear-resistant and anti-friction bulk materials strengthened through precipitation and dispersion hardening. The tribological properties of these materials are contingent upon the dimensions, morphology, quantity, and type of reinforcements present. The carbon nanomaterials, mainly including fullerenes, carbon nanotubes (CNTs), graphene, and nanodiamonds, have been identified as having significant potential as solid additives in bulk materials for achieving high wear resistance and anti-friction performance.^159^ Emerging two-dimensional materials, including graphene, MXenes, hexagonal boron nitride (h-BN), and transition metal dichalcogenides (TMDs), show significant potential for performance enhancement due to their high strength, extensive surface area, minimal thickness, and exceptional thermal and chemical stability.^160^

Structured carbon films dominated predominantly by sp^2^-hybridized carbon exhibit macroscopic superlubricity. However, given the lack of a direct correlation between graphene (or carbon onions) and superlubricity enhancement, it is plausible that friction-induced graphene and carbon onion structures are localized within the central region of the wear track, and presenting lamellar and nanoparticulate morphologies, respectively.^161^ Furthermore, interfacial carbon–carbon interactions are suppressed, and the contact area is reduced, thereby contributing to the realization of macroscopic superlubricity.

A combination of reinforcement and ultrafine-grained microstructure enhances the tribological properties of metallic materials through precipitation hardening and grain boundary strengthening. Severe plastic deformation combined with heat treatments is a highly effective method for modifying the structures of metallic materials, allowing the precise control over critical features including grain size, grain boundary characteristics, dislocation density, precipitate distribution, and solute segregation. Recently, additive manufacturing (AM), which offers a high degree of design and manufacturing flexibility, has shown considerable potential for the *in situ* formation of precipitates. Among all tested samples, the particle-rich zone of the A333/B4C/SiC functionally graded metal matrix composite (FGMMC) exhibited the highest microhardness (198.9 HV) and tensile strength (267.9 MPa). Elemental mapping confirmed the uniform distribution of the ceramic particles and enabled detection of the elemental composition of both composites. The particle-rich layer of the A333/B_4_C/SiC hybrid FGMMC demonstrated enhanced wear resistance.^159^ In silicon nitride composites, oxide sintering aids, including Al_2_O_3_, Y_2_O_3_, MgO, CaO, and others-are crucial for achieving liquid-phase sintering and promoting the α-to-β Si_3_N_4_ transformation. During the cooling process of oxide phases, intergranular glass films (IGFs) are formed at grain boundaries, along with the development of triple point pockets. Hampshire and Pomeroy elucidated the formation mechanism of the glassy phase, specifically identifying its presence as triple point pockets (TPs) and intergranular glass film (IGF) in silicon nitride composites.^162^

It is noteworthy that the addition of 4 wt% Cu promoted the formation of Cr-enriched precipitates in the matrix, which exhibited the partial coherence with Cu nanoparticles. The corrosion resistance of the CCM alloy was enhanced with the addition of 2 and 3 wt% Cu, whereas a decline in performance was observed when the Cu content reached 4 wt%.^163^ For CCM-2Cu and CCM-3Cu, the formation of Cr precipitates was identified as a key factor in improving wear resistance. In the case of CCM-4Cu, the synergistic effect of Cu-based lubrication and Cr precipitates was determined to be the dominant mechanism. The objective of this study was to enhance the tribological and corrosion properties of CCM alloys produced by selective laser melting (SLM) by modifying their microstructure through Cu addition. The findings indicate that the sample with 1 at% B exhibits optimal tribological properties under elevated temperature conditions. Additionally, a morphological transition of borides from blocky to acicular structures was observed with increasing boron content.^164^ The wear rates at both ambient and elevated temperatures were observed to decrease with increasing B contents. This reduction is attributed to the combined effects of enhanced hardness, solution strengthening, and the grain boundary pinning effect induced by the formation of the secondary phase. Interestingly, residual austenite and fine-grained martensite can be functioned as effective reinforcing phases in wear-resistant materials. Laser-induced phase transformation technology, which involves the rapid heating of the metal surface to austenitization temperatures followed by self-quenching, has been shown to significantly improve surface hardness through the formation of martensitic microstructures. Microstructural analyses indicate that this process transforms the near-surface regions of various metallic materials, including carbon steel, alloy steel, stainless steel, cast iron, and aluminum, into the fine-grained martensite, often accompanied by residual austenite or carbide precipitates.^165^ The transformation of residual austenite into martensite can substantially enhance surface hardness, thereby resisting the cutting effect of abrasive particles and the propagation of cracks, ultimately leading to the improved wear resistance. Fine-grained martensite demonstrates the superior wear resistance, due to its high hardness and refined microstructure.^166^

The addition of reinforcing agents, including precipitated phases and secondary phase particles, plays a crucial role in improving the wear resistance of bulk materials. Precipitates and second-phase particles contribute to both precipitation and dispersion hardening, and their impact on wear resistance is strongly dependent on their size, morphology, quantity, and type. Structured carbon films primarily composed of sp^2^-hybridized carbon exhibit macroscopic superlubricity. Although no direct correlation has been established between graphene and enhanced superlubricity, friction-induced graphene and carbon onion structures may contribute to the reduction of contact areas and interfacial interactions, thereby facilitating low-friction behavior. The synergistic effect of reinforcement and ultrafine grains contributes to the enhancement of wear resistance in metallic materials through mechanisms such as precipitation hardening and grain boundary strengthening. Additionally, when the reinforcing materials are combined with ultrafine grain structures obtained through severe plastic deformation, heat treatment, or additive manufacturing, wear-resistant and anti-friction properties of metallic materials are significantly improved.

## 3 Properties of tribological materials

The governing factors that influence a material's tribological characteristics can be classified into two principal domains: extrinsic variables associated with a tribological testing environment and intrinsic properties inherent to the material's microstructure. The impact of experimental variables on the tribological behavior of a diverse array of materials across multiple length scales, from macroscopic to nanometric dimensions, has been rigorously examined through both empirical studies and the tribological simulation analysis.^156^ This section scrutinizes the effects of material's intrinsic properties, such as indentation hardness, elastic modulus, ultimate tensile strength, and resistance to the cyclic plasticity, on its anti-wear and anti-friction capabilities. Additionally, it investigates the correlative interplay between a material's tribological efficacy and these fundamental material characteristics.

### 3.1 Hardness

**Fig. 10:**
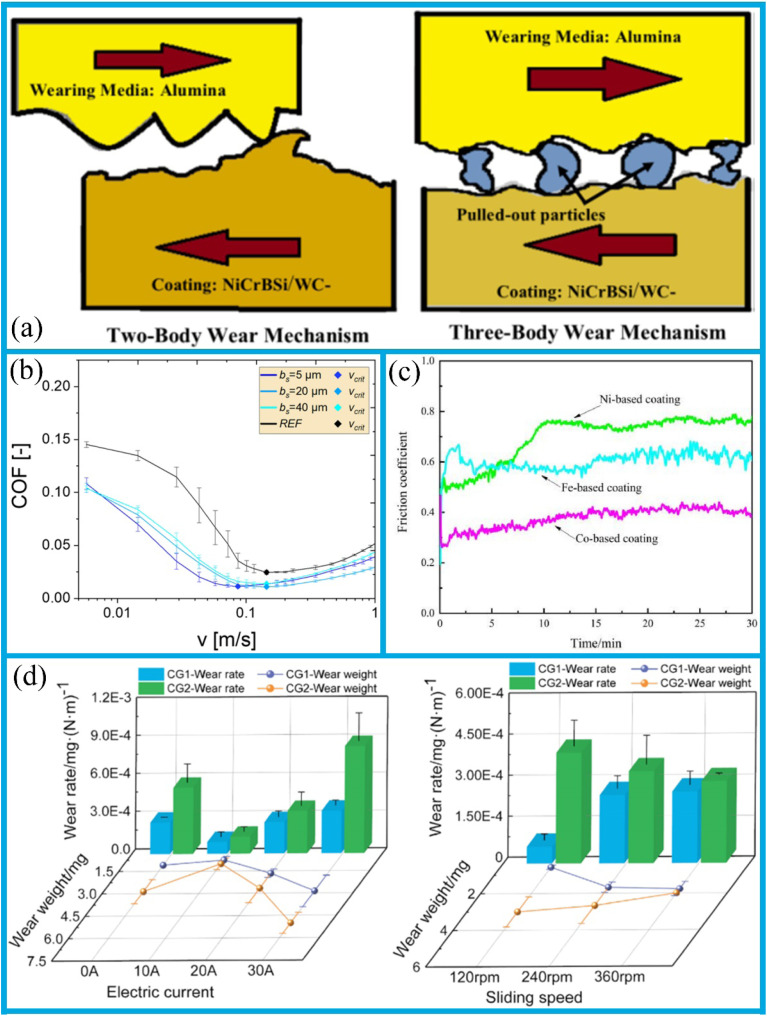
(a) Schematic of wear mechanisms: two-body and three-body. Reproduced from ref. 167 with permission from Elsevier, copyright 2023. (b) Stribeck curves of chevron-shaped microstructures: foot width Bs = 5–40 μm. Reproduced from ref. 168 with permission from Elsevier, copyright 2023. (c) Friction coefficient curves of Ni-, Fe-, and Co-based coatings. Reproduced from ref. 169 with permission from Elsevier, copyright 2023. (d) Wear rates and wear weights of CG1 and CG2 at different currents and different sliding speeds. Reproduced from ref. 170 with permission from Elsevier, copyright 2023.

The excellent impact, fatigue, and tribological properties of coatings in corrosive media at low and medium temperatures are attributed to multiple factors. Furthermore, these coatings are cost-effective and exhibit good hardness and toughness. Despite their demonstrated performance in tribological applications, increased manufacturing productivity has been accompanied by a corresponding increase in the wear rate. Consequently, it is imperative to further enhance the wear resistance and anti-friction properties of these coatings. A particularly effective method involves the addition of hard reinforcement particles to the coating matrix. Relevant studies have ensured that the hardness of the coating increases markedly, when the WC-CoCr mixtures are incorporated.^167^ The enhancement of coating hardness can be attributed to the presence of hardening phases, particularly WC. The efficacy of carbide phases in increasing hardness is influenced by the size of the interstitial spaces between them, which are filled by the soft metal alloys. “Continuity” is defined as the proportion of the surface area where one carbide grain is in direct contact with a grain of different composition. To achieve higher continuity, the distribution of the WC-CoCr mixture should be optimized, as increased continuity leads to the enhanced hardness. As illustrated in Fig. 10a, during the process of friction and wear, the coating generates a series of fine debris particles that loosely adhere to the surface. Fine carbide phase particles are observed to be pulled by alumina balls, resulting in the shallow scratches along the sliding direction, indicating a two-body wear mechanism. When the WC particles are dislodged by the alumina balls, three-body abrasive particles are formed, causing a transition in the wear mechanism to three-body wear. The incorporation of the WC-CoCr reinforced phase significantly enhances the coating's hardness and wear resistance. However, oxidation occurs at elevated temperatures; if the oxide particles fail to form a physically homogeneous layer on a coating surface, they may negatively affect its tribological properties. Additionally, the loose bonding between WC-CoCr flakes and the insertion of alumina particles into the coating structure also impair its frictional properties.

A method provides a better understanding and accurate prediction of the lubrication regime (Stribeck curve) compared to the conventional approach that relies on initial surface roughness. The proposed methodology enables the prediction of variations in lubrication regime parameters, that are characterized by the Stribeck curve, by means of controlled adjustment of the hardness ratio.^171^ As shown in Fig. 10b, the friction coefficients of the linear samples with a spacing of 5–40 μm are consistently lower than those of the unstructured reference samples. Small-scale wedges (bs = 5–40 μm) have been shown to reduce boundary friction by 29%, mixed friction by 85%, and the friction at the transition point from the mixed to fully lubricated state by up to 56%.^168^Fig. 10c illustrates the variation in the friction coefficient during wear testes of Fe-, Ni-, and Co-based alloy-coated samples subjected to the laser-based localized additive repair. The friction coefficient curves of specimens can be divided into two distinct stages: an initial wear stage and a stable wear stage. The friction coefficient of the Fe-based alloy-coated specimen first increases, then decreases, and finally stabilizes. After a break-in period of approximately 10 minutes, the friction coefficient of the Ni-based repaired specimen reaches a stable state. In contrast, the Co-based alloy-coated specimen exhibits a friction coefficients that initially decreases and then increases, primarily due to its significant initial surface roughness. As the coating material undergoes gradual oxidation, the friction coefficients increases rapidly and then decreases.^169^ The hardness was increased to the range of 730–820 HV_0.5_, and the residual stresses were transformed into a compressive state. Rolling contact fatigue (RCF) cracks were observed to initiate and propagate preferentially along the boundary between the dendritic and eutectic phases.^172^ The ZrNb7, when subjected to oxidation under low oxygen partial pressure, exhibits the lowest wear on both the ceramic surface and its UHMWPE counterpart. Additionally, in Calo wear tests, the coating demonstrates superior behavior compared to a martensitic hardened steel (100Cr6) reference material.^173^

The difference in hardness exerts distinct influence on the sliding contact resistance and interfacial temperature rise of the material. As the electric current was varied, the low-hardness materials demonstrated the notable adaptability. The adhesive wear was observed in all materials regardless of the hardness, while the abrasive wear initiated concurrently with the increase in electrical current. As illustrated in Fig. 10d, the wear rates of CG1 and CG2 were reduced to 9.4 × 10^−5^ mg (Nm^−1^) and 1.35 × 10^−4^ mg (Nm^−1^), respectively. With increasing sliding speed, the wear rate of low-hardness material decreases, whereas that of high-hardness material increases, as also shown in Fig. 10d. Notably, the elevated sliding velocity does not cause significant damage to the contact surface of the high-hardness materials, indicating their commendable resilience to the mechanical shocks.^170^

Hardness plays an important role in improving the tribological properties of the coatings, especially wear resistance. The addition of hard reinforcing particle, such as the WC-CoCr mixture, in the coating matrix is an effective strategy for improving both hardness and wear resistance. The presence of hardened phase, with WC playing a predominant role, and the optimization of spatial distribution can enhance structural continuity, thereby increasing the overall hardness of coating. The wear mechanisms of the coating can be divided into two-body and three-body wear. During the wear process, the presence of fine carbide phase particles and their interaction with the abrasive particles contribute to the transition between these wear mechanisms. The incorporation of WC-CoCr significantly enhances the coating's hardness and wear resistance. Additionally, materials with low and high hardness exhibit opposite trends at different sliding speeds: high-hardness materials display superior resistance to the mechanical impacts under high sliding velocities, whereas the low-hardness materials exhibit a decreased wear rate with increasing sliding speeds.

### 3.2 Stiffness

The discovery of new materials with distinctive properties, particularly tribological behavior, constitutes a key research to focus with significant practical and economic implications. Friction and wear represent major contributors to material degradation. In friction and wear processes, the surface layer of a material plays a significant role, and its properties can be effectively modified through surface modification techniques and coating applications. Furthermore, the material core is important for load transfer during the component operation. It can be reasonably inferred that an ideal material should shows high wear resistance and excellent mechanical property. The mechanical properties of the material core can be improved by the incorporation of appropriate additives. For example, an orowan strengthening effect, that arises from the dispersion of nanoparticles within the metal matrix, and the Hall–Petch strengthening mechanism, induced by grain refinement, offer superior improvements in mechanical properties, if compared to micron-scale particle reinforcement. The incorporation of the additional alloying elements into the copper matrix has been shown to effectively increase its hardness, stiffness, axial deformation capacity, wear resistance.^174^ An enhanced mechanism lies in a fact that the high plastic deformation resistance of the top DLC film acts as the primary barrier against the indenter penetration. In contrast, the bottom cermet coating, which combines the advantageous properties of both ceramic and metallic materials, serves as a support layer for the top film, thereby enhancing its protective capability in a manner analogous to armor. Moreover, as the carbide content increased, the hardness improvement of the duplex coating, compared to the single cermet coating, become progressively more significant. This indicates that the aforementioned synergistic effect is primarily dependent on coating behavior, which is determined by material composition, including hardness and stiffness.^175^

**Fig. 11:**
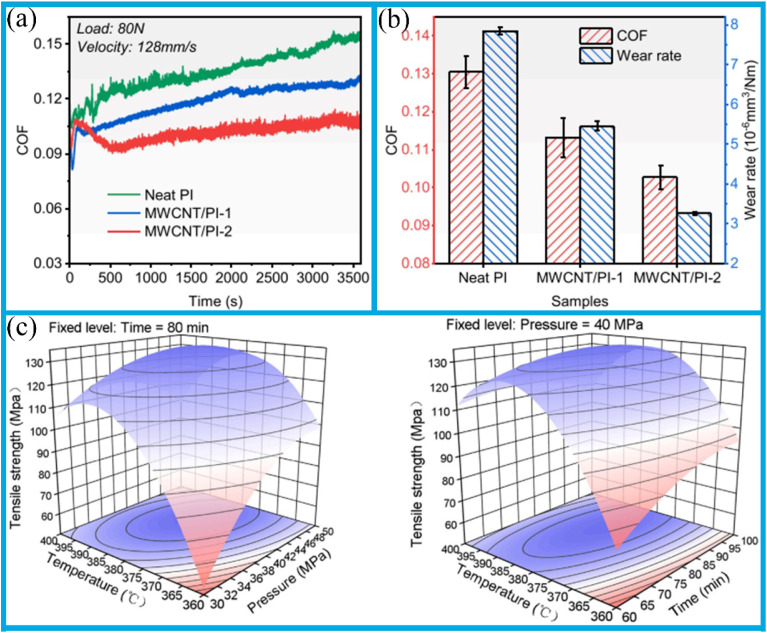
(a) Friction coefficients of the PI and MWCNT/PI composites *versus* test time with different processing parameters. (b) Mean friction coefficient and wear rate. (c) Response surface plots of the effect of processing temperature, pressure, and time on tensile strength. Reproduced from ref. 176 with permission from Elsevier, copyright 2023.

In addition to the structural hardness, matrix hardness has been demonstrated to have a significant influence on wear behavior. Specifically, specimens supported by elastic substrates exhibit higher wear resistance than those supported by rigid substrates. At comparable hardness levels, wear resistance is further influenced by the sliding time. Fig. 11a illustrates the typical friction coefficient curves for PI and its composites. Under reciprocating friction and water lubrication conditions, the COF curves of pure PI and MWCNT/PI-1 show a fluctuating trend over time, that are characterized by an initial increase followed by stabilization. In contrast, the COF curves of MWCNT/PI-2 remain relatively stable between 500 and 3600 seconds. The average COF values and wear rates are presented in Fig. 11b. When compared with the pure PI (COF = 0.1305) and MWCNT/PI-1 (COF = 0.1133), the MWCNT/PI-2 (COF = 0.1028) exhibits a most effective reduction in friction and improved wear resistance, with a corresponding 44% reduction in wear rate.

The stress–strain curve enables the evaluation of three fundamental mechanical properties: stress at rupture, strain at rupture and modulus of elasticity. The properties are intrinsic characteristics of polymers, and reflect the key mechanical behaviors, including stiffness, strength, and toughness.^176^ The objective is to characterize the relationship between the response and the level of each variable, as well as to identify a nature of the interaction between the two experimental variables. Predictive models can be developed, and the visualization of the 3D response surface facilitates the interpretation of the regression model. As illustrated in Fig. 11c, when the holding time is fixed at 80 minutes, moulding temperature exerts a quadratic effect on tensile strength. The elliptical contour plots in the *X*–*Y* plane show a significant interaction between moulding temperature and pressure. At the low pressure, the tensile strength initially increases rapidly and subsequently decreases with rising temperature. When the moulding pressure is maintained at 40 MPa, the moulding temperature exerts a quadratic effect on the tensile strength response (Fig. 11c).

An observed increase in the glass transition temperature of the MWCNT/PI indicates that molding temperature has a significant influence on the tribological properties of MWCNT/PI under water lubrication. Furthermore, the MWCNTs act as molecular bearings, promote both rolling and sliding motions between friction pairs, thereby enhancing lubrication performance.

### 3.3 Strength

The hardness of material serves as a key indicator of its mechanical strength. Under the dry wear condition, the wear resistance of metallic material is largely determined by their hardness. It has been observed that the materials exhibiting higher hardness generally demonstrate the improved wear resistance and a reduced friction. The incorporation of additives can markedly improve the friction and wear performance of composite material. Specifically, the suitable additives can effectively reduce surface wear and fatigue damage, thereby preserving the material's structural integrity and stability. Additionally, the internal micro- and nanostructure of material (*e.g.*, lattice structure, grid structure) exerts a significant role in determining mechanical strength, as well as its wear resistance and friction-reducing properties.

The incorporation of nanofillers into the PI matrix has been widely studied as an effective strategy to enhance its tribological performance. However, due to limitations inherent in conventional manufacturing processes, the polyimide materials with the fully gradient structure have been rarely documented in the literature. Fortunately, the unique capabilities of 3D printing provide a promising approach for the fabrication of such materials. The development of high-performance and durable 3D-printed PI for tribological applications is of critical importance, accompanying by the considerable technical challenges. The 3D-printed PI exhibits the remarkable tensile strength (∼126 MPa) and the excellent Young's modulus (∼3 GPa). Furthermore, the incorporation of MoS_2_ as the lubricant enables the development of PI/MoS_2_ composite inks, facilitating a fabrication of diverse PI-based structure configurations by additive manufacturing. In comparison to 3D-printed pure PI, the incorporation of MoS_2_ into PI gradient structures results in a more stable and lower COF, as well as minimal wear, with a 68% reduction in wear rate.^177^ The incorporation of additive has been observed to reduce material strength; however, their tribological properties are enhanced. This study aims to improve tribological property of Cu-based self-lubricating composites (CMSCs) through the additions of WS_2_ nanosheets. Although the inclusion of WS_2_ nanosheets reduces the mechanical strength of Cu-WS_2_ composite, it significantly improves its tribological behavior, as indicated by a lower friction coefficient and the smaller wear rate. Furthermore, Cu-WS_2_ composites incorporating with the nanoscale WS_2_ flakes exhibit the enhanced mechanical and tribological properties, with a 61.2% reduction in wear rate. The incorporation of WS_2_ nanosheet promotes the refinement of WS_2_ agglomerates and the formation of well-dispersed WS_2_ particles within the Cu-WS_2_ composite matrix. Notably, the enhanced strength of the Cu-WS_2_ composites facilitates the formation of continuous and smooth lubrication film, which effectively inhibits the initiation and propagation of subsurface wear cracks.^178^

In contrast to the metallic materials, the strength of polymer has a multifaceted influence on the wear resistance. Ratner–Lancaster plots demonstrate that the wear resistance of polymer is directly proportional to their tensile strength and elongation at break. An increase in yield strength, without a concomitant change in molecular weight or entanglement density, has a negligible effect on the wear resistance of polymer, that is governed by interchain bonding, chain interactions, and a physical network structure. The abrasion resistance of UHMWPE is rarely dominated by a single tensile property. Moreover, the ratio of maximum contact stress to yield strength (*σ*/*σy*) exhibits a strong correlation with the wear rate, thus suggesting its potential as a key parameter for assessing the wear resistance of the polymeric materials.

### 3.4 Plasticity

The ratio of hardness (*H*) to elastic modulus (*E*), commonly referred to as the “plasticity index”, is a widely accepted parameter for determining the limitation of elastic surface contact, which constitutes a critical factor in wear resistance. In the fields of metals, cermets, and ceramics, the wear rate generally decreases with an increase in the *H*/*E* ratio. A negative correlation between the wear rate and the plasticity index is typically observed, primarily due to the high ductility of metals in comparison to that of cermets and ceramics. Nevertheless, strong correlation is not necessarily applicable to amorphous materials, where an increase in the wear rate is well observed with a higher *H*/*E* ratio. This phenomenon arises from the significant elastic discrepancy between the surface and its substrate is commonly observed in amorphous materials. Such mismatch can generate tensile stress at the surface/subsurface interface near the contact perimeter, that may cause a surface layer to detach from the substrate; consequently, a modification occurs in the wear resistance. Experimental studies suggest that amorphous materials exhibit a wear resistance within the *H*/*E* range of 0.06–0.1. Therefore, it is promising to control the surface elastic modulus at a level of the subsurface, without the significantly compromising hardness, and then enables the fabrication of highly wear-resistant amorphous materials.

The *H*/*E* ratio is regarded as a significant parameter for evaluating the abrasion wear resistance of coatings. Coating thickness, preparation process, microstructure characteristics, and the interfacial adhesion between coatings and substrates are the critical factors that should be considered in further studies to evaluate the relationship between the *H*/*E* ratio and wear resistance.

Ceramics exhibit high *H*/*E* ratios but are inherently brittle due to the presence of covalent and ionic bonds. Their wear mechanisms are primarily governed by contact stress. Under specific operating conditions, the wear resistance of ceramics can be improved by restraining the crack initiation and propagation, which is achievable through grain size reduction.

Plastic deformation can accumulate beneath rubbing surfaces under high cyclic stresses. Ratcheting, a phenomenon characterised by directional accumulation of plastic deformation, may result in a formation of surface crack. This mechanism plays a significant role in the assessment of wear and fatigue behaviors. The crack initiation occurs when the ratcheting strain reaches the ductility limit of the steel. Hence, the critical factors affecting the crack initiation lifetime are the ratcheting strain rate and the ductility limit. The steel with a low carbon content (0.85 wt%) exhibits the consistent crack initiation lifetime across a wide range of the loading conditions, and ensures superior resistance to ratcheting damage compared to the 1 wt% carbon steel. Consequently, it displayed the enhanced wear and fatigue resistance. The model predictions are in agreement with the behaviors observed in an in-service rail steel.

This type of plasticity occurs at the micron scale beneath the surface, specifically at the level of surface convexities or roughness features. It leads to plastic deformation that is confined to a very thin near-surface layer. For instance, the accumulation of plastic deformation in the metal matrix at the coating-matrix interface can induce the fracture, leading to coating detachment and subsequent exposure of the underlying matrix. The COF increases as the tribological coating is gradually removed.^179^ The plastic deformation processes are accompanied by the generation of internal stresses and localized material hardening, referred to as “tribological hardening.”

In contrast to “global” plasticity, “tribological” plasticity is a continuous process. At the roughness scale, the material is unable to achieve an elastically stable state due to the continuous wear process. This persistent “tribological” plasticity determines the overall distribution of shear stress within the contact areas. Assuming that the two metallic contact surfaces remain in full adhesion (no slipping). The rough (uneven) surface asperities come into contact under normal loads and form metallic contact zones. The transverse relative motion, induced by creep, generates shear stress. Ideal plastic deformation occurs when the shear stress reaches the material's yield stress, which is equivalent to the product of the normal stress and the COF. This ideal plastic deformation is governed by the ideal plastic material law and Coulomb's friction law, which may involve a constant or variable COF. But, the idealized plastic deformation mechanism is not fully compatible with the phenomenon of continuous “tribological” plasticity and the accompanying strain hardening effects. This is because “tribological” plasticity affects the overall shear stress distribution in the contact zone, indicating that “global” plasticity and “tribological” plasticity are not as independent from one another as they may initially seem. In the OCD model, the ECF model directly calculates the contact shear stress distribution, which serves as input for the AWD model, thereby integrating tribological effect into an analysis.^180^ Hence, according to Coulomb's law of friction, the COF is no longer an uncertain parameter in the AWD model.

## 4 Applications of tribological materials

The good combination of advanced technologies and materials enhances the tensile strength and surface anti-wear behavior of mechanical components, hence enhancing their operational stability and precision while significantly extending their service life. This leads to superior performance at both the micro- and macro-levels. This section focuses on the application and significant advancements in wear-resistant and anti-friction materials, which are extensively utilized in these aerospace components, automotive systems, wind turbines, micro-electromechanical systems (MEMS) and nanoelectromechanical systems (NEMS), atomic force microscopy (AFM), and the biomedical devices.

### 4.1 Aerospace components

The materials of aviation applications are selected based on their high behaviors, owing to the critical role in the aerospace industry. The ongoing advancement and introduction of the novel materials in aerospace engineering are motivated by the objectives of weight reduction, enhanced fuel efficiency, improved performance, and the cost minimization.^181^

Despite the increasing use of composite and other lightweight materials, aluminium alloys continue to be a preferred choice in the aerospace industry, primarily due to the well-established manufacturing processes, excellent fatigue crack growth resistance, and superior inherent damage tolerance.^182^

The medium carbon microbead-silicon carbide (MCMB-SiC) composites fabricated *via* a liquid silicon infiltration (LSI) method demonstrate the highest flexural strength (210 MPa) and ablation resistance (9.1%) in comparison with G-SiC and the C/C–SiC composites. This phenomenon can be attributed to the *in situ* formation of a silicon carbide network reinforcement resulting from the reaction between liquid silicon and carbon. It is expected that the high flexural strength of MCMB-SiC composites, combined with the cost-effective processing of asphalt-based materials, will facilitate their application in aerospace fields.^186^ With the accelerated advancement of the aerospace industries, the researchers globally are directing their efforts toward the development of reinforced ceramics, particularly ceramic matrix composites (CMCs). These materials theoretically possess a wide range of advantageous properties, such as high-temperature resistance, low density, high specific strength and modulus, as well as excellent oxidation and ablation resistance. It is hypothesised that CMCs may serve as the potential substitutes for metallic materials in structural applications under high-temperature conditions.^187^

**Fig. 12:**
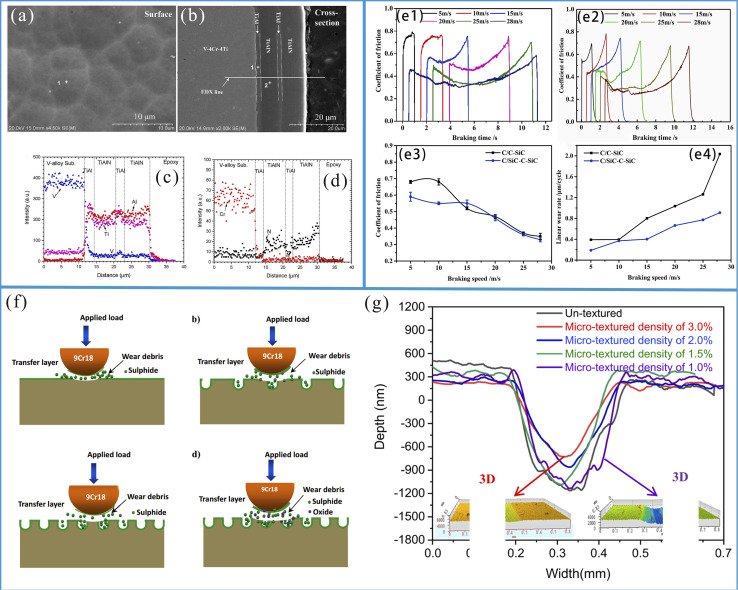
(a) Plain-view SEM image of the surface morphology of the composite coating. (b) Cross-sectional view of the coating. (c and d) EDSX intensity depth profile for major elements of the line marked in (b). Reproduced from ref. 183 with permission from Elsevier, copyright 2023. (e1 and e2) Typical brake curves of C/C/C–SiC and C/SiC–C–SiC brake rings; (e3 and e4) average COF curve and linear wear rate curve of brake pair. Reproduced from ref. 184 with permission from Elsevier, copyright 2021. (f) Schematic diagram of the wear mechanism of the sample after 3.6 × 105 cycles. Reproduced from ref. 185 with permission from Elsevier, copyright 2022.

Vanadium alloys represent a promising candidate for structural components in both Generation IV and fusion reactors. Due to their high reactivity with impurity elements such as hydrogen and oxygen, these alloys necessitate the implementation of surface protection measures. Previous coating strategies for vanadium alloys have primarily relied on the monolithic coatings, which may exhibit insufficient adhesion strength between the coating and its substrate. Although TiAlN-based composite coatings have shown significant potential in providing effective surface protection, their application to vanadium alloy substrates has not yet been investigated. A TiAl/TiAlN composite coating, incorporating an intermediate TiAl layer, is deposited onto a V–4Cr–4Ti alloy substrate using the filtered cathodic vacuum arc deposition (FCVAD). The resulting coating is found to be highly dense, and exhibits no voids or inclusions.^183^ The composite coating displays a homogeneous surface morphology, that is characterized by uniformly distributed fine particles and micro-pits, is free from macroscopic pores or impurities (Fig. 12a). Quantitative EDX analysis indicates a surface composition of Ti_23.50_Al_28.19_N_48.32_ (atomic percentage), which is in the good agreement with the stoichiometric ratio of (Ti_0.5_Al_0.5_) N. This chemical uniformity is further supported by EDX results obtained from the inner TiAlN layer (point 2 in Fig. 12b), and reveals consistent elemental distribution across the entire coating thickness. Cross-sectional analysis (Fig. 12b) confirms the structural integrity of the coating, revealing a defect-free microstructure with the no observable porosity or inclusions. The EDX scanning results obtained from the substrate and composite coating are illustrated in Fig. 12c and d. The elemental distribution aligns with the expected coating composition, with Ti and Al showing higher concentrations in the TiAl layer compared to the TiAlN layer. As shown in Fig. 12b, the coating structure comprises alternating TiAl and TiAlN layers. The TiAl layer is specifically incorporated to reduce the pronounced property mismatch between the ceramic TiAlN layer and the metallic V-alloy substrate, thereby improving the coating's adhesion strength and ductility. In comparison to the substrate, the coating demonstrates significant enhancements in surface hardness and surface roughness.

In a study is conducted by Ma *et al.*,^184^ the incorporation of SiC ceramics into carbon fibre bundles of C/C–SiC composites was examined using the PIP technology. Fig. 12e1 and e2 present a comparison of the braking curves of C/SiC–C–SiC and C/C–SiC braking rings under varying braking speeds. The braking characteristics of the two materials exhibit a high degree of similarity. Fig. 12e3 depicts the variation in the average coefficient of friction (COF) for both materials. The COF behavior of the C/SiC–C–SiC brake ring is comparable to that of the C/C-sic, with the COF gradually decreasing as the initial braking speed increases. Fig. 12e4 illustrates the variation in wear rate. Compared to conventional C/C–SiC, the developed C/SiC–C–SiC composite demonstrates a consistently lower wear rate. It can be inferred that the incorporation of SiC into the C/C region enhances the wear resistance of the C/C–SiC brake ring while exerting minimal influence on the COF behavior. The results indicate that PIP-SiC C/C–SiC composites exhibit notable flexural strength. Furthermore, the strength difference between horizontal and vertical surfaces is significantly reduced. The incorporation of SiC expands the toughening mechanisms of composites, markedly decreases the wear rate, and increases the volume of the localized C/C region.

In the current era of lightweight design, aluminum alloys are widely utilized in the automotive and aircraft industries. But, their application is limited in environments where tribological performance poses a critical challenge. Therefore, the deposition of ceramic coatings onto the metal substrates, such as aluminum alloys, represents an effective strategy for enhancing component durability and extending service life. The incorporation of boron, silicon, and tungsten carbide powders into AA 6061 aluminum alloys through friction stir processing modifies the surface morphology. The resulting surface metal matrix composites (SMMCs) exhibit the improved wear resistance, if compared to the base metal. Among them, the SMMC reinforced with B_4_C (127VHN) demonstrates superior wear resistance relative to SMMCs reinforced with tungsten carbide (WC) and silicon carbide (SiC). The limited wear resistance of the AA 6061 aluminium alloy is primarily attributed to its low surface hardness and the inferior tribological properties. In contrast, the dominant wear mechanism observed in all other surface composites is adhesive wear, which constrains their wear performance. Surface modification of AA 6061 aluminium alloy using B_4_C as the reinforcing phase has been proven to be more effective in enhancing the wear resistance of the resulting composites, when compared to other reinforcing materials such as WC and SiC.^188^

The deposition of a MoS_2_–Ti thin film onto the low-density micro-textured surface of 9Cr18 steel has been demonstrated to improve the long-term tribological properties of precision moving parts in the aviation industry. An Nd: YAG femtosecond pulsed laser system is employed to create dimple densities of 1.0%, 1.5%, 2.0%, and 3.0% on microtexturised surfaces. Feng *et al.*^185^ examined the influence of MoS_2_–Ti thin films with the different micro-texture densities on their tribological behavior and wear mechanisms. A wear mechanism of the MoS_2_–Ti film deposited on both non-textured and textured surfaces is presented in Fig. 12f. After the friction and wear tests, a significant amount of wear debris accumulates within the wear scars and tracks. This phenomenon not only intensifies plowing wear and elevates the friction coefficient, but also impedes the formation of an effective transfer film within wear scars. For low-density microtextured surface, although the microtextures are capable of retaining a certain quantity of wear debris, a significant portion remains on the 9Cr18 balls, leading to inadequate lubrication and elevated friction coefficients for microtextured surfaces with microtexture densities of 1.0% and 1.5%, in comparison to those with densities of 2% and 3%. For the microtextured surface with a density of 3.0%, the microtexture effectively captures the majority of wear debris, facilitating a formation of a transfer layer of optimal thickness. It results in a substantial reduction in both the friction coefficient and wear rate, as demonstrated in Fig. 12g. However, as the number of sliding cycles increases, the amount of wear debris rises. The microtexture becomes incapable of retaining an adequate amount of debris. Moreover, oxidative wear that occurs during prolonged friction further contributes to an increased wear rate. The surface microtexturing not only captures and retains wear debris within the lubricating film, but also promotes the formation of a stable transfer layer, thereby extending the wear life of the composite.

In the aerospace sector, the performance and durability of components can be enhanced through the application of cutting-edge materials and surface modification techniques. Ceramic matrix composites (CMCs) and aluminum alloys serve as two representative material systems: CMCs have been extensively implemented, whereas aluminum alloys exhibit the well-established manufacturing processes and favorable mechanical performance. The performance of these materials can be further enhanced through surface engineering and coating deposition. For example, carbide powders can be utilized to improve the wear resistance of aluminum alloys. The MoS_2_–Ti coating can be applied on micro-textured surface to enhance its tribological properties. However, the optimal texture density is significantly affected by the applied load and sliding speed. A high texture density may lead to an uneven coating thickness. During prolonged sliding, the accumulation of debris can evolve into abrasive wear, thereby accelerating the wear process. Currently, there is no universally accepted standard for this parameter. Furthermore, the incorporation of additives improves the tribological properties of materials. For instance, the addition of SiC to C/C composites enhances both their mechanical strength and tribological performance. But, the incorporation of SiC may influence the thermal conductivity of the C/C matrix, a factor that remains under debate with respect to its impact on high-temperature heat dissipation behavior. Furthermore, the optimal doping level must be determined with consideration of the operational parameters such as braking speed. Excessive doping may compromise the braking stability, indicating the necessity for additional experimental investigations to support a comprehensive understanding.

### 4.2 Automobile parts

**Fig. 13:**
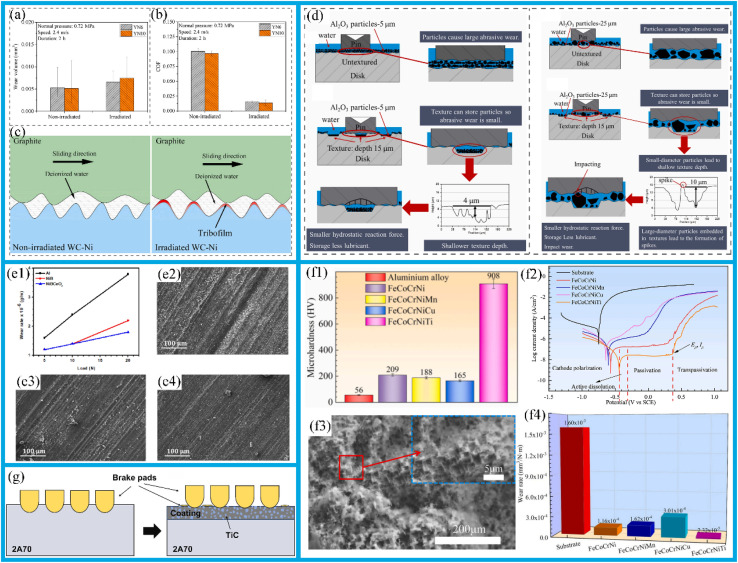
(a) Wear volume of non-irradiated/irradiated WC-Ni surfaces slid against graphite. (b) COF between graphite and non-irradiated/irradiated WC-Ni surface under water lubrication. (c) Friction statures schematics of non-irradiated WC-Ni and irradiated WC-Ni against under deionized water. (d) Schematic of friction and wear mechanisms of untextured and textured samples under the 4 μm particle and 25 μm particle. Reproduced from ref. 189 with permission from Elsevier, copyright 2023. (e1) Wear rate as a function of load applied. (e2) SEM micrograph of the worn surface of A356. (e3) Ni–B and (e4) Ni–B–CeO_2_ coating after pin-on-disk wear testing. Reproduced from ref. 193 with permission from Elsevier, copyright 2018. (f1) The microhardness of the substrate and LC-HEA coatings. (f2) Polarization curves of the substrate and LC-HEA coatings. (f3) Corrosion morphologies of the substrate. (f4) Wear rate of the substrate and LC-HEA coatings. Reproduced from ref. 194 with permission from Elsevier, copyright 2023. (g) Wear mechanism 2A70 and 40%TiC coating. Reproduced from ref. 195 with permission from Elsevier, copyright 2023.

Cemented carbide possesses a series of favorable properties, such as high hardness and excellent wear resistance, which make it a preferred material for its applications requiring resistance to wear and friction, including machining and mining operations. However, further improvements of the tribological performance of cemented carbide components are necessary to expand their applicability to extremely severe operating environments, as well as high-load and high-power conditions. Surface modification technology represents an effective approach to address the wear-related challenges in modern mechanical components.^189^ However, in certain cases, the applications of surface enhancement techniques may exacerbate wear in the cemented carbides. For instance, increased wear rates were observed for HIPIB-irradiated WC-6Ni and WC-10Ni and WC-10Ni during the friction tests conducted with graphite under water-lubricated conditions, as illustrated in Fig. 13a and b. This phenomenon can be attributed to the formation of a tribological layer consisting of W and a minor quantity of Ni. This layer significantly reduces the coefficient of friction (COF) between WC-Ni and graphite, but simultaneously increases the wear rates, as illustrated in Fig. 13c.The improved tribological properties of surface texture can be attributed to the retention of abrasive particles, the retention and storage of lubricants, and the enhancement of the load-bearing capacity through the dynamic pressure effect. The surface texture can effectively entrap the abrasive particles and mitigate abrasive wear; however, its effectiveness in this regard is contingent upon the texture dimensions and the particle size, as shown in Fig. 13d. When the particle sizes are smaller than the texture dimension, the texture is capable of capturing particles, thereby reducing the abrasive wear. Conversely, when the particle size exceeds a texture dimension, larger particles may embed into the texture from the asperities, leading to an impact-induced wear.

The alloys are extensively employed in industrial applications due to their superior tribological performance. Among of these, aluminium-based alloys are widely utilized across a diverse range of fields, including the automotive industry, defence sector, aircraft manufacturing, electrical module packaging, electronics, the automotive body structures, and renewable energy systems such as wind and solar power, owing to their favorable characteristics, such as high specific strength, low weight, excellent erosion resistance, high electrical conductivity, environmental compatibility, and the recyclability.^190^ As one of the most commonly used material types in mechanical applications—particularly structural applications—aluminium alloys continue to offer substantial potential for further research development, with expanding applications in the automotive, energy, and various other industrial sectors. Although numerous aluminium alloys have been investigated by the earlier researchers, AA6262 T6 has received limited attention. Future studies in this area will focus on examining the influence of machining parameters on the turning process and identifying effective optimization strategies.^191^

The automotive industry is experiencing a growing demand for lightweight alloys aimed at reducing vehicle mass. Hence, there is a marked interest in substituting cast iron with aluminium alloys to improve the energy efficiency and comply with increasingly stringent environmental regulations. Aluminium-silicon alloys possess a highly favourable combination of properties, such as high specific strength, excellent formability, superior corrosion resistance, low melt viscosity, the enhanced castability, recyclability, low coefficient of a thermal expansion, the high thermal conductivity, elevated-temperature strength, good resistance to hot tearing, and the advantageous tribological characteristics. The distinctive and beneficial properties have contributed to their extensive applications in the automotive industry.^192^

The alloys are extensively employed in industrial applications due to their superior tribological performance. In particular, aluminium-based alloys are widely utilized in key sectors such as the automotive industry, defence, and other fields.^190^ Globally, the approximately 1.6 billion vehicles, including road vehicles, trucks, trains, ships, and aircraft, are used for transportation. It is estimated that road vehicles consume 83 exajoules (EJ) of energy worldwide, with approximately 32% of this energy being utilised to overcome friction. Furthermore, energy loss attributed to wear constitutes approximately 10% of the energy expended in overcoming friction.^196^

The objective is to improve the wear and corrosion resistance of nickel aluminium bronze (NAB) in marine environments. Laser surface cladding (LSC) offers a viable method for depositing TaC/Stellite X-40 Co composite coatings onto NAB substrates. Homogeneous distribution of carbides and intermetallic reinforcements throughout the matrix contributes to enhanced wear resistance of the materials. Moreover, the presence of fine isometric crystals in the surface region and refined columnar crystals in the vicinity of the substrate establishes a metallurgical bond between the LSC coating and the NAB substrate, thereby improving both wear and corrosion resistance.^197^

Laser additive manufacturing represents a promising method for fabricating near-*net*-shape components from Al-based nanocomposites, which demonstrate superior mechanical and tribological properties. Owing to their inherent lubricity, boron nitride nanosheets (BNNSs) are incorporated into the AlSi10Mg alloy *via* high-speed ball milling and laser metal deposition (LMD) to produce the self-lubricating Al alloy nanocomposites exhibiting exceptional wear resistance and fatigue performance. The study reveals that the number of cycles to failure under the tensile fatigue loading increased from 10^3^ for the pure AlSi10Mg to 10^6^ with the addition of merely 0.1 wt% BNNSs. At a BNNS concentration of 0.2 wt%, a reduction of 58% in the coefficient of friction and 57% in the wear volume is observed for the AlSi10Mg alloy. Scanning electron microscopy (SEM) micrographs ensure that the surface of pure AlSi10Mg displays a worn appearance, is characterised by the presence of grooves, wide ridges, debris, and pronounced protrusions of worn material along the groove edges. The main wear mechanisms in pure AlSi10Mg include plastic deformation, delamination, and adhesion wear. In contrast, the LMD-built AlSi10Mg/BNNS composites exhibit a relatively smooth surface with discernible wear patterns, which can be attributable to the formation of a thin lubricant layer by the extruded BNNSs during the test. An extended finite element model is used to predict crack propagation during fatigue testing, thus showing a good agreement with the experimental results. This study demonstrates that additive manufacturing technology serves as a feasible and effective approach for producing Al matrix composites with engineered properties suitable for various design applications.^198^

Aluminium-based nickel coatings with tailored surface properties are of significant interest for numerous applications, including corrosion protection, wear resistance, and self-lubrication. The nanocomposite coating system exhibits a lower wear rate, if compared to the conventional nickel coating, and this wear rate remains lower than that of the uncoated aluminium alloy (Fig. 13e1–e4). An increase in applied load is associated with a proportional increase in the wear rate. At elevated loads, the wear rate of the nanocomposite is significantly reduced. This phenomenon can be attributed to the formation of a mechanically mixed or intermediate layer, which prevents direct contact between the alloy and the coating during sliding, thereby minimizing material removal in the steady-state wear state. Furthermore, the interfacial bond strength between Ni–B and embedded particles plays a crucial role in the wear behavior. It can be concluded that the reduced wear rate under high loads results from the refined grain size of substrate, the reinforcement-hardening effect, and the enhanced integrity of the cerium particles within the matrix, as compared to those present in the alloy.^193^

The applications of nanocomposite coatings can enhance the wear resistance of aluminum alloys. However, the phenomenon of aluminum dilution, which occurs during the coating process, may compromise the coating quality. To enhance the comprehensive surface properties of aluminum alloys, and mitigate the impact of the aluminum dilution on coating performance, studies have been carried out to fabricate FeCoCrNi-M (M = Mn, Cu, Ti, –) high-entropy alloy coatings using a two-layer laser cladding method.^194^ These coatings can effectively enhance the wear resistance of aluminum alloys while demonstrating superior hardness and corrosion resistance. Hardness testing of the coating (Fig. 13f1) reveals that the hardness of the FeCoCrNi coating without additional alloying elements is 209 HV, which is substantially higher than that of the aluminum alloy substrate (56 HV). The hardness of coating decreases to 188 HV and 165 HV, respectively, after the addition of Mn and Cu. However, the incorporation of Ti leads to a substantial increase in coating hardness, reaching 908 HV. This enhancement is attributed to lattice distortion induced by the large atomic radius of Ti, as well as precipitation and dispersion strengthening effects arising from the formation of the BCC phase and Laves phase (Fe_2_Ti), and the contribution of fine-grain strengthening. Electrochemical tests are conducted in a 3.5 wt% NaCl solution indicate that the corrosion resistance of FeCoCrNi-M coating is significantly higher than that of the aluminum alloy substrate. As illustrated in Fig. 13f2, the aluminum alloy substrate exhibits no passivation zone, with a corrosion current density as high as 1.55 × 10^−5^ A cm^−2^. In contrast, all four coatings display the distinct passivation zones; among of these, the FeCoCrNiTi coating exhibits the lowest corrosion current density (1.15 × 10^−8^ A cm^−2^), which is approximately 1/1300th that of the substrate, thereby demonstrating the best corrosion resistance behavior. From the corrosion morphology (Fig. 13f3), the substrate exhibits a loose and porous structure after the corrosion, accompanied by the formation of a significant amount of corrosion products. In terms of wear performance (Fig. 13f4), the wear rate of the FeCoCrNi coating is 1.16 × 10^−4^ mm^3^ (N^−1^ m^−1^), which is significantly lower than that of the substrate (1.60 × 10^−3^ mm^3^ (N^−1^ m^−1^)). Upon the addition of Mn or Cu, the wear rates increase to 1.62 × 10^−4^ mm^3^ (N^−1^ m^−1^) and 3.01 × 10^−4^ mm^3^ (N^−1^ m^−1^), respectively. However, upon the addition of Ti, the wear rate decreases to 2.32 × 10^−5^ mm^3^ (N^−1^ m^−1^), which is only 1.45% of that of the substrate. This reduction is attributed to the anti-plastic deformation capability of the high-hardness phase and the wear-inhibiting effect of composite structure, resulting in an improvement in wear resistance by approximately 69 times. Furthermore, Jin *et al.*^199^ examined the influence of TiC content on the microstructure evolution, phase transformation, and mechanical properties of 2219 Al–Cu alloy at the deposition boundary. Their findings revealed that the incorporation of TiC particles induced a transformation from columnar crystals into equiaxed crystals. The presence of the dot phase and dispersed TiC particles within the grains promotes dislocation generation, thereby enhancing both the strength and toughness of the 2219 Al–Cu alloys. The resulting tensile strength reached 384 MPa, with an elongation of 18.3%. The process has been demonstrated to enhance the corrosion and wear resistance of the fabricated components. The preparation of wear-resistant coatings on the surface of aluminium alloys to replace cast iron for lightweighting urban railways brake discs represents a promising and cost-effective approach. TiC particles are incorporated into aluminium-based composite coatings at varying mass percentages (20%, 40% and 60%), and deposited on the surface of 2A70 high-strength aluminium alloy *via* the laser direct deposition. The addition of TiC particle results in a reduction in the number of pore defects in the molten pool, a phenomenon is attributed to the stirring effect induced by the particles. The 40% TiC coating exhibits the highest shear strength. The principal constituents of the coatings are α-Al and TiC. The uniformly distributed TiC particles effectively prevent a penetration of foreign matter in the coatings, thereby enhancing wear resistance.^195^ As illustrated in Fig. 13g, in the absence of coating protection, hard asperities on the material surface exert a ploughing effect on the relatively softer 2A70 surface. The resulting abrasive debris becomes entrapped between the friction pairs and cannot be effectively removed in time. This leads to a transition in the wear mechanism from two-body abrasive wear to the three-body abrasive wear. When the coating contains a high content of TiC particles, the surface remains notably smoother, with atoms on the two contacting surfaces nearby and demonstrating strong adhesive interactions. Additionally, a uniform distribution of TiC particle effectively suppresses surface extrusion and ploughing, thereby enhancing the wear resistance of the coating.

Surface modification technology plays an important role in improving the tribological properties of cemented carbide and aluminum alloys. Methods such as surface texturing and laser deposition welding have proven to be effective in improving the wear resistance and anti-friction performance of these materials. In the case of aluminum alloys, advanced strategies including laser additive manufacturing, the applications of nanocomposite coatings, the usage of high-entropy alloys, and the incorporation of TiC particles have significantly enhanced wear resistance and other favorable properties, particularly for automotive components. But, the current surface modification technologies face a trade-off between enhancing behavior and mitigating associated side effects. For example, partial modification of cemented carbides may increase the wear rate. In the case of aluminum alloy coatings, a key challenge arises from the contradictory effect of aluminum dilution on overall performance. While the addition of the TiC to aluminum alloys can enhance hardness, it often compromises material toughness. Under extreme operating conditions, such as high-speed impact loads, the reduction in toughness may even result in a significant increase in the wear rate. Therefore, it is essential to determine the optimal particle content in conjunction with the specific type of load. Furthermore, there is ongoing debate concerning the trade-off between laser technology, which offers the lower cost and higher efficiency. Accordingly, the selection of most suitable technology should be based on the specific application requirements.

### 4.3 Wind turbines

**Fig. 14:**
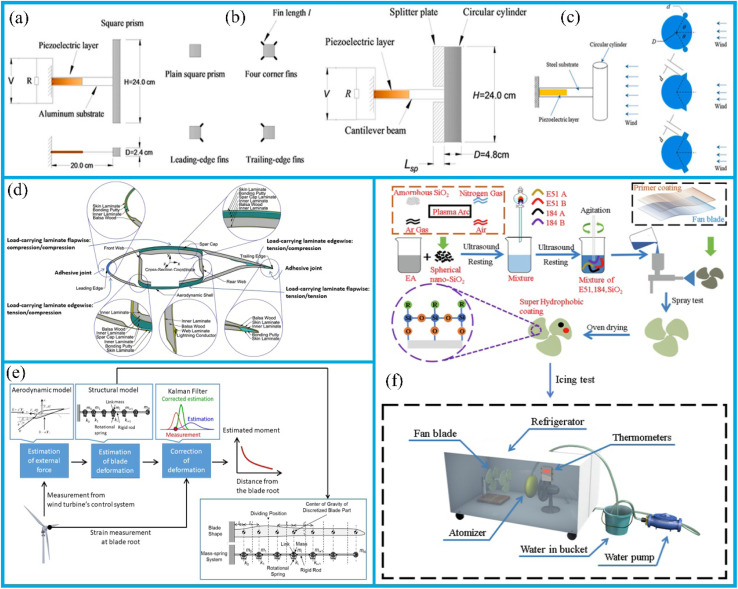
Schematic of wind energy harvester with different types of attachments attached to the bluff body: (a) with fins to the corners of the square prism; (b) with splitter plate to the leeward sides of the circular cylinder; (c) with rods to the circular cylinder. Reproduced from ref. 200 with permission from Elsevier, copyright 2023. (d) Schematic diagram of a wind turbine blade section; a cross-section of the Clipper C96 wind turbine blade is referenced. (e) Overview of blade load estimation using digital twin technology, adapted from. Reproduced from ref. 202 with permission from Elsevier, copyright 2023. (f) Preparation of the superhydrophobic coating and icing test. Reproduced from ref. 203 with permission from Elsevier, copyright 2023.

Owing to rapid population and economic growth in the past few decades, there has been great concern about the potential use of renewable resources. Wind energy, in particular, has garnered significant attention from urban builders and researchers as a promising avenue for promoting the sustainable development of urban environments.^200^ Recent studies have demonstrated that the aerodynamic optimization of blunt bodies, as illustrated in Fig. 14a–c, is an effective strategy for improving wind energy performance. As shown in Fig. 14a, the efficiency of the square prism wind energy collector is markedly improved after the installation of fins. When compared to the conventional square prism configuration, maximum power collection enhancement ratio can reach 150%. Wind energy collectors based on vortex-induced vibration (VIV) are constrained by the upper limit of the resonance range. To overcome this limitation, Song *et al.*^201^ studied a piezoelectric wind energy collector incorporating a splitter plate positioned in the wake of a cylinder within a wind tunnel, as shown in Fig. 14b. Experimental results demonstrated that this configuration effectively eliminates the upper threshold of the wind speed range for wind energy collection. When a splitter plate with a length of 0.65D was installed on the leeward side of the cylinder, the performance of the VIV-based wind energy collector reached its peak. Hu *et al.* developed a cylinder-based piezoelectric wind energy collector, which incorporates two small rod-shaped attachments on the main cylinder. The study found that when the rods of three different cross-sectional shapes (circular, triangular, and square) were arranged at angles of 45° or 60° around the main cylinder, the wind energy collector was capable of continuously harvesting wind energy even beyond the critical wind speed, as shown in Fig. 14c. Among the various rod configurations and installation angles, the arrangement involving two triangular rods placed at a 60° angle relative to the main cylinder produced the highest lateral force, thereby markedly enhancing the efficiency of wind energy collection.

Wind energy is extensively utilized in contemporary applications, and there is an increasing emphasis on enhancing the efficiency of its utilization. Consequently, the research has been initiated on wind turbine blade materials and their corresponding coatings.^204^ Wind turbine composite blades are susceptible to various forms of the damage and defects, including friction-induced wear, delamination, debonding, and cracking, as a result of exposure to multiple structural loads and severe operational conditions. These failure mechanisms represent the primary modes of the degradation observed in wind turbine composite blades.^202^ Turbine blades are manufactured with thin walls and pre-twisted to address deformations from bending and twisting during operation. Pressure loads cause both torsional deformation and bending along the blade edges and flaps. As shown in Fig. 14d, a conventional wind turbine blade includes a load-bearing beam made of a laminate and a web. This beam supports the shell and delivers the required global and local strength and stiffness. The main beam's flap-direction laminates resist compressive, tensile, and torsion loads, while its edge-direction performance helps to prevent damage from leading- and trailing-edge bending. To accurately assess the fracture mechanics of a blade load, the dynamic response of the blade can be predicted by combining static load assessment based on a Supervisory Control and Data Acquisition (SCADA) system with Kalman filtering. This method estimates blade loads using strain measurements taken at the blade root (Fig. 14e).

Wind turbine blades often encounter operational challenges such as icing and wear when they are exposed to harsh environmental conditions. Studies have shown that SiO_2_ spherical nanoparticles, obtained *via* RF plasma spheroidal spraying, and mixed with E51, PDMS, and ethyl acetate, can be applied to the surface of aluminium plates and polyurethane-primed wind turbine blades, offering an effective solution to these issues.^203^ The coating preparation process is illustrated in Fig. 14f. The small interfacial area between water and the coating, which arises from the surface micro- and nanostructures, together with the coating's high hydrophobicity, endows it with strong resistance to icing. Furthermore, the coating displays the notable self-cleaning capabilities against contaminants and exhibits excellent abrasion resistance.

As a renewable resource, the wind energy has great development potential. An aerodynamic optimization techniques can effectively improve efficiency. For example, attaching fins, separating plates, or rods to bluff bodies has been demonstrated to improve wind energy harvesting. Improving rotor blade materials and coatings can address the issues such as amination, delamination, and cracking in composite wind turbine blades, thereby improving their long-term sustainability. In addition, spherical silica nanoparticle coatings provide anti-icing, self-cleaning, and excellent tribological properties, effectively addressing the operational challenges faced by wind turbine blades in harsh environmental conditions.

### 4.4 Micro-/nano-electromechanical systems

MEMS and NEMS devices are highly sensitive to the surface forces and adsorbed species due to their high surface-to-volume ratios. Consequently, they are increasingly used as sensitive probes in basic surface science research, a field that employs device technologies based on silicon, metals, diamond, graphene, and carbon nanotubes.^82^ Micro-nano electromechanical systems (MEMS/NEMS) are designed in detail to operate across the time scales ranging from milliseconds to picoseconds, imposing the significant demands on the material adhibition. In the following context of the BioMEMS/BioNEMS, the friction and wear behavior of the biological layer becomes particularly critical. It is well established that the mechanical properties of such systems are inherently size-dependent.

The usage of an effective lubricant can improve tribological properties, minimize wear, promote the rapid bonding of micro/nano device surfaces, and enhance overall durability. The performance requirements for the micro-electro-mechanical systems (MEMS) and nano-electromechanical systems (NEMS) are particularly important. Nanoscale changes in adhesion, friction, and wear may have a significant impact on product precision. To ensure optimal protection, use of a lubricant is recommended, with perfluoropolyether-based lubricants demonstrating the high effectiveness for this purpose. Manuel Palacio *et al.*^205^ conducted an investigation into wear patterns on silicon surfaces using atomic force microscopy. The experiment results indicate that the partially bonded mobile lubricant assemblies, when combined with heat-treated coatings, provide the superior wear protection for silicon surfaces compared to fully bonded and untreated coatings. It is advisable to supplement the good system with lubricating oil to further improve the protective performance.

**Fig. 15:**
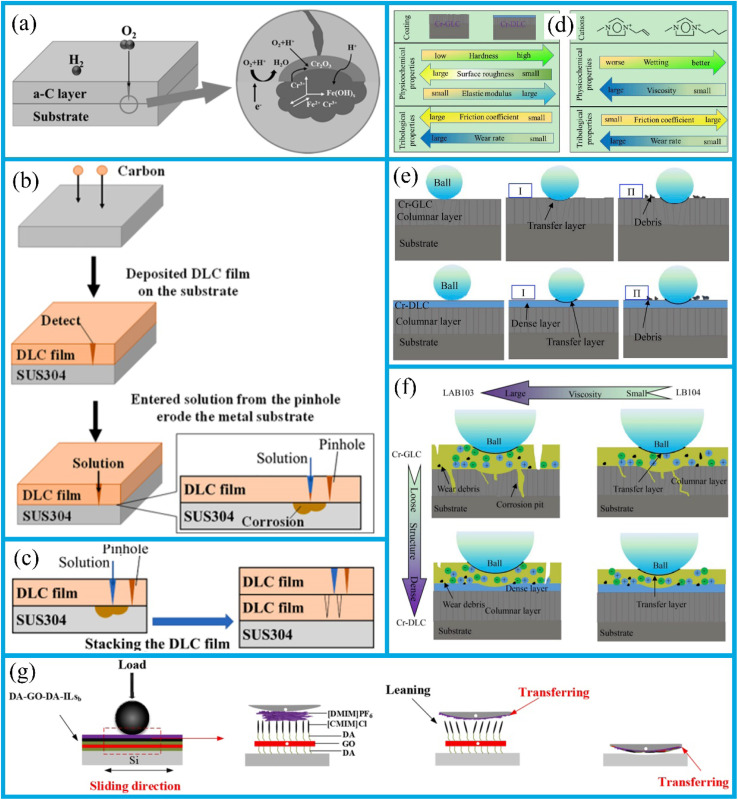
(a) Schematic of possible interface-induced degradation mechanism. (b) Corrosion process for DLC-coated samples. (c) Schematic diagram of corrosion protection mechanism by laminating the DLC film. Reproduced from ref. 206 with permission from Elsevier, copyright 2021. (d) The relations between the structures and lubricant properties: solid coatings and ILs. (e) Schematic diagram of the wear mechanism of the two coatings under dry conditions. (f) Schematic diagram of the wear mechanism of the two coatings with ILs. Reproduced from ref. 207 with permission from Elsevier, copyright 2020. (g) Schematic diagram of the tribological mechanisms of (DA-GO-DA)-ILsb. Reproduced from ref. 208 with permission from Elsevier, copyright 2022.

Diamond-like carbon (DLC) coatings exhibit the excellent properties for industrial applications, such as low friction, wear resistance, and superior chemical stability.^206^ The incorporation of silicon (Si) can further enhance the tribological behavior of DLC coatings. Specifically, the DLC coating containing 0.8% Si shows the highest level of friction-induced hardening. Based on microfriction test results obtained from the wear track after the macro-test, the average microfriction coefficient is related to the thickness of secondary structure, the silicon content of coating, and the specific volume wear of coating, with the DLC + 5% Si coating exhibiting the highest specific volume wear. Additionally, DLC coatings have exhibited the stable film formation in acidic environments (Fig. 15a). The enhanced corrosion resistance can be attributed to the reduction in pinhole defects generated during the deposition of the DLC coating, which results from the lamination of the DLC coating. The reduction in defect area is illustrated in Fig. 15b. DLC coatings demonstrably enhance the corrosion resistance of metallic materials when exposed to hydrochloric, nitric, and sulphuric acids. Furthermore, stacked DLC coatings exhibit enhanced corrosion resistance in metallic materials. A schematic representation of the corrosion protection mechanism of the DLC coating is presented in Fig. 15c. Yan *et al.*^207^ prepared Cr-doped graphite-carbon (Cr-GLC) coatings and Cr-doped diamond-like carbon (Cr-DLC) coatings *via* PVD and PECVD, respectively. The lubrication behavior of solid–liquid composite lubrication system is studied using two ionic liquids as lubricants. The results show that, under the lubricated conditions, the friction coefficients of the composite systems decreased by approximately 40% compared to the dry condition, exhibiting a good synergistic lubrication effect. Cr-DLC coatings show the better tribological properties than Cr-GLC coatings, which can be attributed to their better physicochemical film formation and higher microstructure density under friction conditions. The synergistic effect of the composite system is mainly influenced by the microstructure characteristics of the film layer, as well as the viscosity and corrosiveness of the ionic liquids (ILs). The performance of solid–liquid composite lubrication system depends on the physicochemical properties of both liquid and the coating. The interrelationship between the microstructure and tribological behavior is illustrated in Fig. 15d. The friction and wear process leads to the generation of wear debris from the grinding balls, along with chemical reactions occurring between the friction counterpart and oxygen under the influence of mechanical contact. The wear mechanisms associated with both coatings are schematically illustrated in Fig. 15e.

Neither coating exhibits the significant plastic deformation, indicating a favourable synergy effects between boundary lubrication and solid lubrication in the solid–liquid composite lubrication system. A schematic diagram of Cr-DLC/IL system is presented in Fig. 15f. Although Cr-DLC coatings generate a greater quantity of the wear debris, they exhibit minimal large-scale spalling. This phenomenon can be attributed to the lower hardness of Cr-GLC coating, which is associated with the presence of a higher concentration of sp^2^ C bonds, thereby reducing their shear resistance. Furthermore, ILs penetrate more readily through the columnar structure of Cr-GLC coating, thereby weakening the interfacial adhesion between coating and its substrate. The inherent corrosive nature of ILs compromises the bonding strength, consequently reducing the wear resistance of the coating. In contrast, a dense microstructure of Cr-DLC coating effectively resists the corrosive impact of ILs, thereby ensuring superior tribological properties. Liu *et al.*^208^ prepared composite lubricating films comprising interlayer graphene oxide (GO) and binary ionic liquids (ILs) on the silicon (Si) surfaces. The tribological properties of these composite lubricating films are significantly enhanced compared to those of bare silicon surface and GO-only surface. Notably, a composite lubrication film exhibits the reduced adhesion and friction at the nanoscale, with the decreases of approximately 65% and 60%, respectively, compared to bare Si surfaces. Furthermore, a 43% decrease in the friction coefficient of GO film is observed at the microscale. As the ball slides, [DMIM]PF_6_ molecules containing long alkyl chains are transferred to a ball surface, resulting in a formation of lubrication layer. Additionally, the presence of long alkyl chains in [DMIM]PF_6_ molecules facilitates the complete coverage of the ball surface by the transferred layer (Fig. 15g). Consequently, (DA-GO-DA)-ILs exhibit exceptional tribological characteristics, particularly in terms of wear resistance under high loads and an exceptionally long wear life. The interlayer GO/binary ILs composite lubrication films are anticipated to be widely used in the nano/MEMS applications, due to their low adhesion, high load-bearing capacity, and excellent anti-wear properties at both the nanoscale and microscale.

These lubricants and coatings mainly contribute to the enhanced performances of micro/nanoelectromechanical systems (MEMS/NEMS). Specifically, the preparation of diamond-like carbon (DLC) coatings, especially when combined with silicon or chromium, can improve the tribological properties of MEMS/NEMS, reduce the wear, and enhance the corrosion resistance. However, this remains a debate regarding the optimal proportion of doping elements. Although silicon (Si) doping can enhance the extent of friction-induced hardening, an excessive Si content (*e.g.*, 5%) may decrease the coating density and increase the micro-friction coefficient. In the case of Cr-doped coatings such as Cr-DLC and Cr-GLC, the incorporation of Cr results in varying the proportions of sp^2^ and sp^3^ hybridized carbon bonds. Cr-DLC coatings are more prone to form a dense tribolayer during the friction process, and exhibit the superior wear resistance. Nevertheless, the performance differences and applicable ranges of these two coating types under varying friction conditions have not yet been fully elucidated. When they are used as the lubricants, ionic liquids (ILs) can also significantly reduce friction, when they are utilized in combination with the DLC coatings. Furthermore, composite lubricating films fabricated from graphene oxide (GO) and ionic liquids (ILs) exhibit the improved nanoscale tribological properties, indicating their potential for applications in the future MEMS/NEMS devices.

### 4.5 Atomic force microscopes

Nanomanufacturing technology is extensively utilised for the fields of science and engineering. Among of the various nanomachining technologies, vibration-assisted nanomachining based on atomic force microscopy (AFM) offers a cost-effective and straightforward approach for fabricating the nanoscale structures. The resolution and quality of the machined features are contingent upon the radius and sharpness of the tip; thus, it is imperative to investigate the wear behaviour of the tip and determine its expected lifespan during nanomachining. In experimental studies, the evolution of tip wear is characterized and modeled to enable the prediction of tip wear and service life in nanomachining processes.^209^ In addition to direct measurement of the tip radius using the scanning electron microscope (SEM), it is found that the attractive force between the AFM tip and the sample surface is strongly correlated with the tip radius. This correlation allows for the direct measurement of tip wear without removing the tip from the AFM. Tip wear can affect the accuracy and resolution of AFM imaging. Linear micropatterns, specifically microgrooves, are prepared using femtosecond laser technology. Lateral Force Microscopy (LFM) studies of laser-ablated microgrooves have shown that when a worn AFM tip is used for imaging, the friction within and surrounding the microgrooves is significantly reduced, when compared to the original surface. This reduction is attributed to the increased tip radius, which results in lower frictional resistance, under the influence of capillary forces. However, when a wear-resistant diamond coating is applied to the tip, the friction contrast is inverted, with elevated friction observed in the laser-modified region.^210^

The accuracy and reliability of AFM measurements depend on the wear resistance of the probe. Gou *et al.*^211^ investigated the deposition of composite diamond and diamond-like carbon (DLC) coatings on silicon probes to reduce the artefacts caused by tip wear. Diamond-DLC composite films are deposited onto standard AFM silicon probes using plasma-enhanced chemical vapor deposition (PECVD). The morphology and composition of the coatings are well characterized through the scanning electron microscopy (SEM), atomic force microscopy (AFM), and the Raman spectroscopy. Experimental results show that the deposition of diamond-DLC composites on silicon probes effectively reduces the probe adhesion and wear, thereby prolonging the operational lifespan of the probes.

Vibration-assisted nanomachining based on the AFM provides a cost-effective and straightforward method for producing nanoscale structures. The resolution and quality of the machined features rely on the tip's radius and sharpness. Experimental findings indicate that the attractive force between the tip and sample surface is correlated with the tip radius, thereby enabling direct measurement of tip wear without removing the tip from the AFM. The accuracy and reliability of AFM measurements depend on the probe's wear resistance. A deposition of diamond-DLC composite coating on silicon probes effectively reduces adhesion and wear, extends the operational lifespan of the probes, and thus enhances the reliability and accuracy of AFM measurements. But, the presence of such coatings may reduce tip sharpness or cause delamination due to potential interfacial bonding deficiencies. Additionally, in tip wear measurement, the *in situ* measurement method based on the relationship between tensile force and radius, although eliminating the need for unloading and offering operational simplicity, is susceptible to the measurement inaccuracies influenced by sample characteristics, environmental humidity, and other external factors.

### 4.6 Biomedical devices

Human tooth enamel must be capable of withstanding the cyclic contact forces, the wear, and corrosion processes involved in normal oral functions. Furthermore, unlike other human tissues, dental enamel does not possess a significant capacity for healing or self-repair. Consequently, the durability of natural teeth in the oral environment is largely contingent upon the fatigue and tribological properties of an enamel.^212^ A variety of implant materials have been employed in the dental applications, selected depending on their efficacy and availability. A successful dental implant must exhibit essential characteristics, including biocompatibility, corrosion resistance, favorable tribological properties, adequate mechanical properties, and osseointegration to ensure its safe and optimal utilization.^213^

**Fig. 16:**
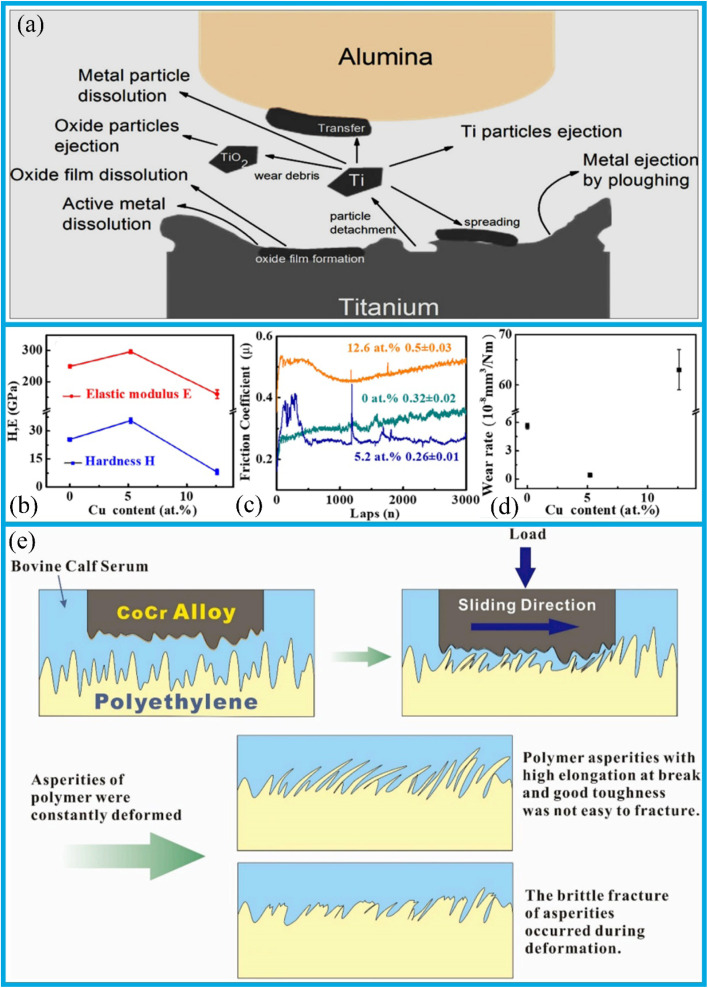
(a) Tribocorrosion processes of Ti implants. Reproduced from ref. 213 with permission from Elsevier, copyright 2022. (b) Hardness *H* and elastic modulus *E*. (c) Friction coefficient curves as a function of sliding cycle. (d) Wear rate on the worn surface for the samples with 0 at% Cu, 5.2 at% Cu, and 12.6 at% Cu. Reproduced from ref. 218 with permission from Elsevier, copyright 2018. (e) Polymer surface wear model. Reproduced from ref. 219 with permission from Elsevier, copyright 2020.

A variety of metal materials, mainly including the stainless steel, cobalt-chromium alloy, precious metal alloys, titanium, and titanium alloys, exhibit superior mechanical properties and favorable processability. It is reasonably deduced that the materials are well-suitable for application as biomaterials in the replacement of load-bearing hard tissues, such as bones and teeth. The titanium alloys have been extensively utilized in various applications due to their lightweight, high strength, corrosion resistance, and biocompatibility. Titanium is the material of choice for the majority of dental implants, owing to its excellent osseointegration ability, favourable biocompatibility, and well-documented clinical success. Additionally, it can offer significant economic benefits.^214^ The erosive effects of chewing on the TiO_2_ coating of titanium implants can result in the material loss; in severe cases, the mechanical failure of dental implants and the restorations occur. A variety of tribocorrosion processes can occur when the ductile metal Ti comes into contact with a highly inert counterpart, such as aluminum oxide, as shown in Fig. 16a.^213^ The application of surface modification techniques has been demonstrated to enhance corrosion resistance, mitigate wear, reduce metal ion release, and promote osseointegration of the biomaterials.^215,216^

The usage of various artificial material has become an essential component of the modern medical practice. Currently, the materials used for hip and knee joint implants mainly include polymers, metals, and ceramics; the materials are largely overlooked for over three decades. Among these, Ni–Ti, Cu–Al–Ni, Cu–Zn–Al, UHMWPE, PTFE, and PEEK, play a vital role in the knee and hip replacement procedures, thus offering the enhanced mechanical property and superior biocompatibility, if compared to other materials.^217^ To extend the service life of artificial joints, it is essential to enhance the mechanical and tribological properties of prosthetic materials. This objective can be achieved by applying a surface protective layer. The present study focuses on the TiCuN solid-solution coating, which demonstrates the exceptional suitability for this specific application. The TiCuN solid-solution coating containing 5.2 at% Cu dopant exhibits the superior mechanical properties, including higher hardness and a lower friction coefficient, as well as enhanced wear resistance, compared to both pure TiN and TiN/Cu nano-composite coatings (Fig. 16b–d).^218^ Furthermore, the live/dead staining results and morphological characteristics of the cells cultured on the TiCuN solid-solution coating indicate that this coating shows the excellent biocompatibility, thereby positioning it as a promising candidate for usage for the protective coating in artificial joint implants within the human body.

Ultra Low Wear Polyethylene (ULWPE) is a novel metallocene-catalyzed high-density polyethylene (HDPE) material. Previous studies have shown that ULWPE exhibits a good biocompatibility and enhances wear resistance, thereby indicating its considerable potential for application in artificial joint systems. However, as a newly developed material, its tribological behavior and underlying mechanisms governing its wear resistance remain insufficiently understood. Compared to the most commonly used materials for the artificial joints, ULWPE shows the most favorable tribological properties, with the lowest level of surface wear.^219^ As illustrated in Fig. 16e, the surface of low-hardness polyethylene undergoes deformation under applied load and relative sliding due to its low hardness, leading to an increase in surface roughness. The uneven protrusions of polymer materials are continuously elongated under cyclic compressive and shear stresses until they exceed the material's elongation limit, at which point fracture occurs. High hardness, strength, ductility, and good wettability of the ULWPE materials contribute to minimizing the damage caused by abrasive wear, thereby endowing an excellent wear resistance.

Wu *et al.*^220^ employed a one-step hydrothermal method to *in situ* synthesize the ZnO nanocomposites on the surface of PEEK powders, thus enhancing the interfacial bonding between the ZnO nanocomposites and the PEEK matrix. Fig. 16a–d shows the synthesis process of PEEK-based nanocomposites. To improve the dispersion and bonding of the reinforcing materials, the synthesis method, as shown in Fig. 16a, is adopted. To avoid agglomeration, nano-ZnO and PEEK are combined *via* hydrogen bonds, as shown in Fig. 16b. Fig. 16c presents the Fourier transform infrared (FTIR) spectra of nano-ZnO, nano-ZnO-PEEK, and pure PEEK, confirming the formation of hydrogen bonds between nano-ZnO and PEEK. Fig. 16d characterizes the effect of nano-ZnO on the crystal structure of PEEK using X-ray diffraction (XRD). The results show that the *in situ* synthesis of nano-ZnO on the PEEK surface do not alter the crystal structure of either the matrix or the reinforcing materials, and then the nano-ZnO exhibits a relatively high degree of purity. The findings demonstrate that the compressive strength of PEEK-based nanocomposites could achieve a maximum of 319.2 ± 2.4 MPa. Both PEEK and PEEK-based nanocomposites are found to show the no cytotoxic effects. Additionally, the PEEK-based nanocomposites indicate the significant antibacterial efficacy against *Escherichia coli* and *Staphylococcus aureus*, with the antibacterial activity increasing proportionally with the nano-ZnO increase.

**Fig. 17:**
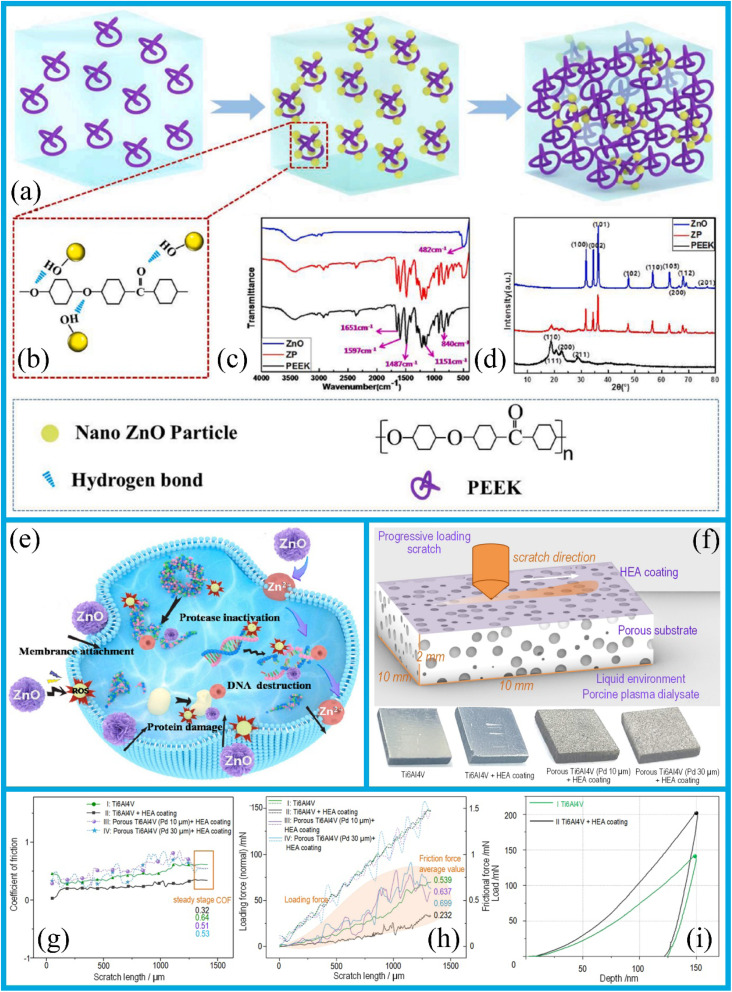
Synthesis and fundamental characteristics of composite materials: (a) synthesis diagram of composite materials; (b) synthesis mechanism diagram; (c) FT-IR spectrum; (d) X-ray diffraction pattern; (e) antibacterial mechanism of a new type of PEEK-based nanocomposites. Reproduced from ref. 220 with permission from Elsevier, copyright 2022. (f) Schematic diagram of the progressive scratch on the surface of specimens. Scratch responses of the artificial joint material consisted of porous Ti substrate and FeCoNiTiAl HEA coating, (g) illustrated the change trends of coefficient of friction (COF) along with the scratch length; (h) shows the corresponding change trends of normal load response and frictional force; (i) nanoindentation load/depth curves for Ti6Al4V and Ti6Al4V + HEA coatings. Reproduced from ref. 221 with permission from Elsevier, copyright 2022.

As illustrated in Fig. 17e, the primary mechanisms through which PEEK-based nanocomposites inhibit the bacterial growth are contact-mediated and photocatalytic reactions. In the contact antibacterial mechanism, the ZnO disrupts the bacterial cell membrane and compromises cellular integrity *via* electrostatic interactions, leading to the leakage of cellular contents. In aqueous environments, Zn^2+^ ions are gradually released from ZnO and subsequently binds to proteases. This interaction leads to the inactivation of proteases and interferes with the physiological functions of bacterial cells. Additionally, the reactive oxygen species (ROS) generated by the ZnO under illumination, particularly ultraviolet (UV) radiation, exhibit a high chemical reactivity, and are capable of reacting with various microorganisms, finally causing microbial death. Furthermore, when the ZnO content reaches 5%, the wear rate of PEEK-based nanocomposite is reduced by 68%, if compared to that of pure PEEK. Consequently, the PEEK-based nanocomposites exhibit dual functionality, combining excellent wear resistance with the effective antimicrobial properties.

Osteolysis refers to the resorption or degradation of bone tissue, process commonly associated with the accumulation of polyethylene particles and the misalignment of the hip implant components. The application of coatings to hip replacement devices has been shown to significantly enhance their mechanical properties and interfacial adhesion, thereby improving wear resistance and extending the service life of the hip implant.^222^

The usage of soft metals, including silver, lead, tin, and gold, as coatings facilitates the formation of an anisotropic lattice structure that is prone to intergranular slippage under low shear stress conditions. Hence, this phenomenon leads to an exceptionally low friction coefficient (approximately 0.1). The addition of Ag and Cu nanoparticles at five distinct concentration ratios to a TiAlN coating has been shown to significantly reduce the friction coefficient of the coating. The primary wear mechanism of TiAlN coatings is abrasive wear. However, the lubricating effect exerts by the soft metal particles changes the main wear mechanism from abrasive to adhesive wear, which consequently contributes to the reduced friction coefficient observed in the composite coating.

In total hip arthroplasty, the femoral head is a critical source of two significant complications and serves as a primary contributor to early implant failure. Magnetron sputtering technology enables the fabrication of Ta multilayer coatings, which are capable of improving the corrosion resistance of CoCrMo alloys, and then reducing a wear rate of polyethylene (PE). Monolayer and multilayer Ta coatings are deposited *via* magnetron sputtering at room temperature, under a deposition pressure ranging from 0.3 to 1.9 Pa. The resulting coatings show hardness and Young's modulus values in the range of 8–21 GPa and 179–288 GPa, respectively. At the elevated deposition pressures, Ta coating shows excellent adhesion property. When compared to uncoated samples, the wear rate of PE is reduced by 4% *via* the application of Ta multilayer coatings. Additionally, the corrosion resistance of Ta multilayer coating is found to be 61% higher than that of the uncoated CoCrMo alloys. These findings indicate that Ta coatings hold the significant potential for applications in artificial joints.^223^

Developing an artificial joint material with mechanical properties that closely match those of bone, while ensuring good biocompatibility, friction resistance, and wear resistance, remains a significant challenge. Liu *et al.*^221^ enhanced the friction and wear resistance of a Ti6Al4V substrate by optimizing its porosity and depositing a high-entropy alloy (HEA) coating. Fig. 17f presents the schematics of the progressive surface scratches observed for the four specimens used in the experiment. As shown in Fig. 17g, the coefficient of friction (COF) of specimen II (with a HEA coating) reaches a stable phase after a brief increase. The friction fluctuating behavior showed by the coated porous specimens (Specimen III and IV) is more pronounced, as shown in Fig. 17h. The accumulation of wear debris during frictional wear leads to an increase in the COF. Conversely, the presence of small pore in material can effectively trap wear debris, thereby reducing the COF (Fig. 17i). Following the applications of the coatings, a 50% reduction in the friction coefficient is observed, indicating that the HEA coating exhibits a significant anti-friction effect. The porous design is an effective approach to reducing a stress-shielding effect and improving hydrodynamic lubrication performance. A combination of the pore structure-induced flexibility and HEA coating-induced surface hardening results in a composite structure with a lower COF, reduces the wear volume, and enhances scratch resistance. This design concept is expected to promote the development of new medical implant materials that fulfill both anti-friction and anti-wear functional requirements.

Titanium alloys are widely used in dental implants due to their biocompatibility and mechanical properties. But, surface modifications such as the application of coatings are essential to enhance the wear resistance and reduce metal ion release. Polymers, metals, and ceramics are commonly used as the materials for artificial joints. Surface modifications, such as TiCuN coatings, can improve the mechanical and tribological properties, thereby extending the service life. Ultra-low-wear polyethylene (ULWPE) shows potential for application in artificial joints due to its favorable biocompatibility and excellent wear resistance. Additionally, composite materials can improve both the mechanical strength and antibacterial properties of the substrate. For instance, ZnO nanocomposites that are fabricated on PEEK substrates effectively achieve this dual enhancement. Application of a high-entropy alloy (HEA) coating on a porous matrix can significantly improve the frictional and wear properties of artificial joint materials. Future research directions will emphasize the development of materials exhibiting the bone-mimicking mechanical characteristics, as well as the continued optimization of surface modification techniques and composite systems to improve the behaviors of the biomedical devices.

## 5 Conclusion and outlooks

### 5.1 Concluding remarks

This review analyzes the advancements in wear reduction, covering innovative surface and substrate design strategies, the intrinsic material properties, and their diverse applications. It reveals the mechanisms by which surface engineering and substrate strengthening inhibit wear, and clarifies the correlation between intrinsic material properties at different scales and wear resistance. Surface engineering technologies include four aspects: coating design, surface texturing, surface hardening, and surface structure construction. These technologies mainly enhance surface wear resistance by introducing a surface layer harder than the substrate or forming a lubricating structure. In coating technology, nanocomposite coatings, amorphous coatings, 2D material coatings, amorphous/nanocrystalline coatings, and the gradient multilayer coatings have been ensured to show exceptional wear resistance under specific working conditions (*e.g.*, corrosive environments, water lubrication, and electrical contact). The optimal design of surface coatings with the specific textures achieves remarkable anti-wear properties under high contact stresses, due to multiple mechanisms: storing lubricating oil, trapping wear debris, and enhancing hydrodynamic pressure effects. These mechanisms can reduce the friction coefficients by up to 75–80%. Surface hardening can effectively restrain plastic deformation and abrasive wear *via* the fore-said mechanisms such as grain refinement and the formation of a hardened layer. Surfaces with the hierarchical structures exhibit a significant wear reduction through multi-scale synergy, with a wear rate three orders of magnitude lower than that of the unprocessed surfaces. Two-dimensional nanomaterials, that are mainly characterized by strong in-plane covalent bonds, ultra-thin thickness, high mechanical strength, and flexibility, are promising components for hierarchical anti-wear structures.

Studies confirm that several strategies for enhancing the wear resistance of bulk materials while preserving their mechanical or electrical properties are viable. These strategies primarily involve microstructural and phase composition modifications of the bulk materials in question. Specific attention will be given to the anti-wear effect of carbon nanomaterial reinforcements, that is dependent on their dimensions, morphology, and content. MD simulation results indicate that adding graphene to polymers produces a strengthening effect, as van der Waals interactions between graphene and the polymer matrix restrict the movement of surrounding polymer chains.

The inherent properties of materials exhibit multidimensional characteristics in regulating their wear resistance. Among of these properties, the hardness is widely recognized as a key parameter in determining a material's wear resistance. Generally, materials with high hardness exhibit a lower wear rate and better wear resistance. But, insufficient data exists for a comprehensive analysis of synergistic effects from other intrinsic properties, such as the stiffness, strength, and cyclic plasticity, on wear resistance. Enhancing wear resistance in bulk materials requires controlling hardness, fracture behavior, plasticity, and strength. These properties are influenced by factors including lattice distortion, bonding strength, grain sizes, precipitation, grain boundaries, dislocations, and twins. For metallic materials with average grain sizes above 10 nm, wear resistance follows classical Archard theory. In contrast, a significant deviation from the Archard theory is observed, when the average grain size is below 10 nm. A discrepancy is mainly attributed to localized hardening of the worn surface, that is caused by grain growth and grain boundary relaxation during repetitive sliding. The role of substrate stiffness in surface wear resistance varies within the observation scales (macroscale or nanoscale). The preliminary correlation between polymer wear and the ratio of maximum contact stress to yield strength has been established, and this correlation will be used to evaluate the polymer wear resistance. Studies have shown that the wear resistance of tribological systems such as ceramic–ceramic, ceramic-metal, and metal–metal depends on the *H*/*E* ratio (hardness-to-Young's modulus ratio). This conclusion is supported by these data from sliding systems including Al_2_O_3_–CrN, Al_2_O_3_–TiC, and steel–steel.

### 5.2 Current challenges and future perspectives

The significant progress of numerous innovative wear-resistant materials in both fundamental research and practical applications has generated the expected challenges, which are particularly critical for the future energy-saving research. Existing high-performance coatings often rely on the complex microstructures to synergistically achieve high hardness and low friction. However, such complex microstructures are susceptible to degradation under cyclic friction or high-temperature conditions. Non-equilibrium coatings preparation mainly depends on extreme conditions, compromising precision. Hence, future efforts should focus on balancing structural simplification and behavior synergy *via* the approaches such as simplified collaborative design of low-dimensional structures, dynamic regulation of self-organization effects, simulation-based design of environment-adaptive coatings. Hierarchical architectures significantly enhance the tribological performance but face persistent challenges: the contradiction between structural design and performance coordination, limitations in preparation processes, and insufficient dynamic adaptability to the environments. Hence, the modular design, intelligent preparation technologies, and dynamically responsive structures are breakthrough directions to translate laboratory achievements into the engineering applications, and meet the demands of complex working conditions. Surface texture technology enhances the material's wear resistance. However, there is no unified theory for anti-friction and anti-wear mechanisms. Owing to the numerous variables in texture design and the diversity of working conditions, its effects vary significantly. As a result, a selection of texture morphology and parameter design still relies heavily on extensive experimental trial-and-error, with only a few generalized rules summarized. Moreover, the theoretical models that effectively predict test results are insufficient. Although the texture processing technologies have achieved breakthroughs at micro/nano-scale, combining different processing methods to develop the superior technologies remains an important direction. Additionally, optimizing surface textures *via* computer algorithms and integrating texturing technology with other surface technologies to enhance wear resistance and anti-friction performance, that are key areas for future exploration. Current research predominantly focuses on the positive effects of surface textures, largely ignoring the problems and challenges in determining research directions. There is also a lack of systematic analysis of potential limitations, which leads to the hidden challenges in its development.

The design of nanostructures or phase compositions *via* optimizing preparation and post-treatment parameters is an effective strategy to further enhance material wear resistance. This strategy focuses on forming nano-twins, heterogeneous or gradient nanostructures, and homogeneously distributed *in situ* precipitates, as well as enabling cryogenic deformation and the well-proportioned dispersion of 2D nanomaterials in matrices with high interfacial compatibility. Computational modelling, typically by *ab initio* calculations and MD simulations, theoretically describes nanostructural features and underlying mechanisms of structural design with phase transformation. Structural reproducibility and stability are critical in large-scale wear-resistant material design. Achieving spatial uniformity, thermal stability of nanostructures, and inhibiting the coarsening of nanocrystals, are two directions that can be explored in the future. Gradient nanostructured coatings and heterojunction nanocomposites can enhance their wear resistance and stability by leveraging interface strengthening effects. Thus, they are potential materials for the future research.

Over the past decade, the research in additive manufacturing (AM) has grown considerably, particularly in the successful fabrication of lightweight and high-performance polymer, ceramic, and metal structures. However, data analysis of the wear behavior of AM-fabricated material remains scarce. Hence, substantial research is required to assess the anti-wear performance of these materials. Of particular interest are AM-fabricated metal materials with nanocrystalline or nano-twinned structures, amorphous materials, and the high-entropy alloys. These materials have demonstrated exceptional mechanical properties and considerable potential in anti-wear applications.

An importance of superlubricity in minimizing the friction and wear-induced damage has increased. However, stable fabrication of superlubric surfaces has currently been achieved only under specific surface conditions at micro/nanoscale, or at the macroscale for minutes. Recently, two-dimensional materials show great potentials to extend superlubricity into reliable, practical applications, depending on their structure and dimension. An effective utilization of 2D material-wrapped nanoparticles would facilitate the maintenance of superlubricious contact over an extended timescale. Further experimental identification, modelling simulations, and the theoretical descriptions of two-dimensional materials are required. Future research should focus on: manufacturing large-scale low-defect-density materials, developing heterostructures, and modifying functional-group-containing materials.

Under high-temperature conditions, materials often undergo severe wear due to accelerated oxidation, mechanical property degradation, and thermal damage of the friction interface. But, it is challenging to experimentally characterize the microstructure *in situ* during sliding, and establish a dynamic correlation between the “temperature-structure-wear rate” remains difficult. Therefore, developing an *in situ* high-temperature friction platform to track real-time evolution of the oxide and phase transformation layer has the theoretical and practical importance for revealing high-temperature wear mechanisms and optimizing material tribological properties. Molecular dynamics (MD) simulations are applied to the micron-sized systems—large enough to contain grains—but are inefficient for the micron-scale polycrystalline systems. Thus, constructing a cross-scale simulation framework to correlate atomic-level defects with the macroscopic wear rates is crucial. These simulations enable the systematic observation and good analysis of deformation mechanisms like dislocation slip, grain boundary migration, and twinning. The atomic-resolution simulations facilitate an in-depth analysis of dislocation-grain boundary interactions—a critical factor influencing material wear resistance. Further, such simulations can clarify the relationship between high-temperature microstructure changes and wear behaviors, significant aiding the development of high-temperature wear-resistant materials. In summary, notable achievements and potential challenges in this field are poised to stimulate the further research, and potentially impact the fundamental materials science and advancing highly wear-resistant materials for the advanced applications.

## Conflicts of interest

The authors declare no conflicts of interest.

## Data Availability

No new data were generated during this study. All analyzed datasets are publicly available and cited appropriately.

## References

[cit1] Liu X. (2003). *et al.*, Characterisation of engineered surfaces by a novel four-in-one tribological probe microscope. Wear.

[cit2] Brockett C. (2023). Biomechanics and Tribology of Total Ankle Replacement. Foot Ankle Clin..

[cit3] Lu T.-t. (2023). *et al.*, Improved anti-adhesive wear performance of rail/armature pair *via* interfacial energy modulation for electromagnetic launching applications. Scr. Mater..

[cit4] de Paula A. F. M. (2023). *et al.*, Synergism between tribological parameters – “micro-abrasive concentration level”, “micro-abrasive particle type”, and “liquid type” of a micro-abrasive slurry composition on the micro-abrasive wear behaviour of Fe-30Al-6Cr (at.%) iron aluminide alloy. Wear.

[cit5] Wang K. (2023). *et al.*, Surface treatment of rail to enhance rolling contact fatigue and wear resistance: Combined spot laminar plasma quenching and tempering method. Constr. Build. Mater..

[cit6] Wei A. (2023). *et al.*, Corrosion wear behavior of 30CrNi2MoVA steel in simulated seawater. Mater. Lett..

[cit7] Zou L. (2023). *et al.*, Experimental and numerical study on the fretting wear-fatigue interaction evolution in press-fitted axles. Int. J. Fatigue.

[cit8] Liu Y. (2023). *et al.*, Macroscopic ultra-low friction and wear enabled by carboxylated graphene with glycerol. Appl. Surf. Sci..

[cit9] Fischer A. (2023). *et al.*, Topography rules the ultra-mild wear regime under boundary lubricated gross-slip fretting corrosion. Wear.

[cit10] Giannakopoulos A. E. (2022). *et al.*, The cohesive zone crack analogue for fretting fatigue based on mild wear. Eng. Fract. Mech..

[cit11] Lin Z. (2023). *et al.*, Clarifying the importance of the running film to the ultra-low wear of the polymer composite by eliminating its individual effect. Tribol. Int..

[cit12] Wang Y.-b. (2021). *et al.*, Relation of normal load with test temperature at mild–severe wear transition state for Mg–Gd–Y–Zr alloy. Trans. Nonferrous Met. Soc. China.

[cit13] Barros L. Y. (2019). *et al.*, Effect of pressure in the transition between moderate and severe wear regimes in brake friction materials. Wear.

[cit14] Mariño F. (2023). *et al.*, Effect of the addition of coated SiO_2_ nanoparticles on the tribological behavior of a low-viscosity polyalphaolefin base oil. Wear.

[cit15] Niu Y. (2023). *et al.*, Tailoring tribological properties of Ti-Zr alloys *via* process design of laser surface texturing and thermal oxidation. Surf. Interfaces.

[cit16] Shang S. (2023). Constructed “sandwich” structure to obtain recyclability and high rejection rate high-flux CA@ HMSN@ h-BN/PDA membrane for efficient treatment of dye wastewater. Colloids Surf., A.

[cit17] Schubert D., Wolf M., Drummer D. (2023). Interaction of tool and process design on the mechanical and tribological behaviour of an injection-moulded polyamide-steel gear set. Polym. Test..

[cit18] Kamerling S., Schlarb A. K. (2019). Locally induced chemical conversion processes — A means to control tribological properties of polymer composites?. Compos. Sci. Technol..

[cit19] López X. A. (2023). *et al.*, Processing of Co-base/C-nanotubes compound coatings on D2 steel using plasma transferred by arc: Tribological and mechanical performance. Surf. Coat. Technol..

[cit20] Shen H., Wang L. (2020). Formation, tribological and corrosion properties of thicker Ti-N layer produced by plasma nitriding of titanium in a N2-NH3 mixture gas. Surf. Coat. Technol..

[cit21] Xie X. (2021). *et al.*, The microstructure and tribological properties of M50 steel surface after titanium ion implantation. Appl. Surf. Sci..

[cit22] Kong Y., Ma S., Zhou F. (2024). Bioinspired Interfacial Friction Control: From Chemistry to Structures to Mechanics. Biomimetics.

[cit23] Chinmay M. (2024). *et al.*, Microstructural Modification, Mechanical Properties, and Wear Behaviour of Aged Al-Si-Mg/Si3N4 Composites for Aerospace Applications. Int. J. Lightweight Mater. Manuf..

[cit24] Wang S. (2024). *et al.*, Recent advances in wear-resistant steel matrix composites: a review of reinforcement particle selection and preparation processes. J. Mater. Res. Technol..

[cit25] Kalsar R. (2024). *et al.*, Material flow behavior and microstructural refinement of AA6061 alloy during friction extrusion. Mater. Charact..

[cit26] Deng J., Dong J., Cohen P. H. (2018). Development and characterization of ultrasonic vibration assisted nanomachining process for three-dimensional nanofabrication. IEEE Trans. Nanotechnol..

[cit27] Khan S. A. (2024). *et al.*, A comparative study in the tribological behaviour of different DLC coatings sliding against titanium alloys. Wear.

[cit28] Deng J. (2016). *et al.*, AFM-based 3D nanofabrication using ultrasonic vibration assisted nanomachining. J. Manuf. Process..

[cit29] Campos Neto N. D. (2022). *et al.*, Influence of Al/(Al + Cr) ratio and doping effects on wear and molten aluminum attack resistance in AlCrN-based PVD coatings for lube-free aluminum die casting. J. Mater. Res. Technol..

[cit30] Ścigała A. (2021). *et al.*, From binary to multinary copper based nitrides – Unlocking the potential of new applications. Coord. Chem. Rev..

[cit31] Yoshimura S. (2022). *et al.*, Low-energy oxygen ion beam induced chemical vapor deposition using methylsilane or dimethylsilane for the formation of silicon dioxide films. Thin Solid Films.

[cit32] Brom J. E. (2016). *et al.*, Hybrid physical–chemical vapor deposition of Bi2Se3 films. J. Cryst. Growth.

[cit33] He R. (2018). *et al.*, Simple fabrication of rough halloysite nanotubes coatings by thermal spraying for high performance tumor cells capture. Mater. Sci. Eng., C.

[cit34] Bai H. (2021). *et al.*, A review on wear-resistant coating with high hardness and high toughness on the surface of titanium alloy. J. Alloys Compd..

[cit35] Wang Q. (2022). *et al.*, Microstructure and properties of Ni-WC gradient composite coating prepared by laser cladding. Ceram. Int..

[cit36] Kumar A. (2019). *et al.*, Optimization of mechanical and corrosion properties of plasma sprayed low-chromium containing Fe-based amorphous/nanocrystalline composite coating. Surf. Coat. Technol..

[cit37] Huang S. (2021). *et al.*, Achieving superlubricity with 2D transition metal carbides (MXenes) and MXene/graphene coatings. Mater. Today Adv..

[cit38] Dearnaley G. (1990). Ion beam modification of metals. Nucl. Instrum. Methods Phys. Res., Sect. B.

[cit39] Sun X. (2022). *et al.*, Research progress in surface strengthening technology of carbide-based coating. J. Alloys Compd..

[cit40] Wu S. (2021). *et al.*, Process parameter optimization and EBSD analysis of Ni60A-25% WC laser cladding. Int. J. Refract. Met. Hard Mater..

[cit41] Liu Y. (2022). *et al.*, Enhanced surface composite coating on Ti811 alloy by laser cladding towards improved nano-hardness. Ceram. Int..

[cit42] Zhang H. (2016). *et al.*, Preparation, mechanical and anti-friction performance of MXene/polymer composites. Mater. Des..

[cit43] Shu F. (2019). *et al.*, Effects of laser power on microstructure and properties of laser cladded CoCrBFeNiSi high-entropy alloy amorphous coatings. Surf. Coat. Technol..

[cit44] Liu N. (2021). *et al.*, Tribological behavior of plasma-sprayed metal based solid self-lubricating coatings under heavy load. Wear.

[cit45] Masuko M. (2015). *et al.*, Friction and wear characteristics of DLC coatings with different hydrogen content lubricated with several Mo-containing compounds and their related compounds. Tribol. Int..

[cit46] Mutyala K. C. (2015). *et al.*, Deposition, characterization, and performance of tribological coatings on spherical rolling elements. Surf. Coat. Technol..

[cit47] Simmonds M. C. (2000). *et al.*, Mechanical and tribological performance of MoS_2_ co-sputtered composites. Surf. Coat. Technol..

[cit48] Ye M. (2016). *et al.*, Microstructure and tribological properties of MoS_2_ + Zr composite coatings in high humidity environment. Appl. Surf. Sci..

[cit49] Zabinski J. S. (1995). *et al.*, The Effects of Dopants on the Chemistry and Tribology of Sputter-Deposited MoS2 Films. Tribol. Trans..

[cit50] Kong N. (2020). *et al.*, A study on the tribological property of MoS(2)/Ti-MoS(2)/Si multilayer nanocomposite coating deposited by magnetron sputtering. RSC Adv..

[cit51] Scharf T. W., Kotula P. G., Prasad S. V. (2010). Friction and wear mechanisms in MoS_2_/Sb_2_O_3_/Au nanocomposite coatings. Acta Mater..

[cit52] Singh H. (2015). *et al.*, An investigation of material and tribological properties of Sb_2_O_3_/Au-doped MoS_2_ solid lubricant films under sliding and rolling contact in different environments. Surf. Coat. Technol..

[cit53] Ren F., Bellon P., Averback R. S. (2016). Nanoscale self-organization reaction in Cu–Ag alloys subjected to dry sliding and its impact on wear resistance. Tribol. Int..

[cit54] Beake B. D. (2015). *et al.*, Wear performance of different PVD coatings during hard wet end milling of H13 tool steel. Surf. Coat. Technol..

[cit55] Abu-Thabit N. Y. and Makhlouf A. S. H., Chapter 24 – Recent Advances in Nanocomposite Coatings for Corrosion Protection Applications, in Handbook of Nanoceramic and Nanocomposite Coatings and Materials, ed. A. S. H. Makhlouf and D. Scharnweber, Butterworth-Heinemann, 2015. pp. 515–549

[cit56] VanEvery K., Krane M. J. M., Trice R. W. (2012). Parametric study of suspension plasma spray processing parameters on coating microstructures manufactured from nanoscale yttria-stabilized zirconia. Surf. Coat. Technol..

[cit57] Ren B. (2020). *et al.*, Tribological properties and anti-wear mechanism of ZnO@graphene core–shell nanoparticles as lubricant additives. Tribol. Int..

[cit58] Renz A. (2018). *et al.*, High-temperature sliding wear behaviour of Stellite®12 and Tribaloy®T400. Wear.

[cit59] Cakmak E., Tekin K. C., Malayoglu U. (2013). Tribocorrosion of Stellite 706 and Tribaloy 400 superalloys. Tribol.-Mater., Surf. Interfaces.

[cit60] Renz A., Kürten D., Lehmann O. (2017). Wear of hardfaced valve spindles in highly loaded stationary lean-burn large bore gas engines. Wear.

[cit61] Jiang K. (2013). *et al.*, Microstructure and tribological properties of solution-treated Tribaloy alloy. Wear.

[cit62] Ya W. (2018). *et al.*, Cladding of Tribaloy T400 on steel substrates using a high power Nd:YAG laser. Surf. Coat. Technol..

[cit63] Kathuria Y. P. (2000). Some aspects of laser surface cladding in the turbine industry. Surf. Coat. Technol..

[cit64] Manjunatha M., Kulkarni R. S., Krishna M. (2014). Investigation of HVOF Thermal Sprayed Cr3C2-NiCr Cermet Carbide Coatings on Erosive Performance of AISI 316 Molybdenum steel. Procedia Mater. Sci..

[cit65] Yaghtin A. H. (2015). *et al.*, Corrosive wear behavior of chromium carbide coatings deposited by air plasma spraying. Ceram. Int..

[cit66] Barbezat G., Nicol A. R., Sickinger A. (1993). Abrasion, erosion and scuffing resistance of carbide and oxide ceramic thermal sprayed coatings for different applications. Wear.

[cit67] Zheng Z. B. (2013). *et al.*, Erosion–corrosion of HVOF-sprayed Fe-based amorphous metallic coating under impingement by a sand-containing NaCl solution. Corros. Sci..

[cit68] Katakam S. (2014). *et al.*, Laser assisted Fe-based bulk amorphous coating: Thermal effects and corrosion. J. Alloys Compd..

[cit69] Zheng Z. B. (2015). *et al.*, Effect of heat treatment on the structure, cavitation erosion and erosion–corrosion behavior of Fe-based amorphous coatings. Tribol. Int..

[cit70] Yao H. , et al., Microstructure and Properties of FeCrB Alloy Coatings Prepared by Wire-Arc Spraying, 2017, 26, pp. 483–491

[cit71] Liu W. , et al., Hot Corrosion Behavior of a Centimeter Fe-Based Amorphous Composite Coating Prepared by Laser Cladding in Molten Na_2_SO_4_ + K_2_SO_4_ Salts, 2015, 270, pp. 33–38

[cit72] Botta W. J. (2014). *et al.*, Corrosion resistance of Fe-based amorphous alloys. J. Alloys Compd..

[cit73] Huang D. (2011). *et al.*, Fretting wear behavior of bulk amorphous steel. Intermetallics.

[cit74] Cheng J. B. (2013). *et al.*, Microstructure and Mechanical Properties of FeBSiNb Metallic Glass Coatings by Twin Wire Arc Spraying. J. Therm. Spray Technol..

[cit75] Poplavsky A. I. (2018). *et al.*, Effect of nitrogen ion irradiation parameters on properties of nitrogen-containing carbon coatings prepared by pulsed vacuum arc deposition method. Vacuum.

[cit76] Kolpakov A. Y. (2011). *et al.*, Nanometer-sized carbon coatings on a silicon wafer: the effect that nitrogen doping level has on specific conductivity and morphology. Nanotechnol. Russ..

[cit77] Zavidovskii I. A. (2019). *et al.*, The Effect of the Ion Assistance Energy on the Electrical Resistivity of Carbon Films Prepared by Pulsed Plasma Deposition in a Nitrogen Atmosphere. Phys. Solid State.

[cit78] Petukhov D. I. (2021). *et al.*, Preparation, chemical features, structure and applications of membrane materials based on graphene oxide. Mendeleev Commun..

[cit79] Yi S. (2021). *et al.*, *In situ* formation of tribofilm with Ti_3_C_2_T_*x*_ MXene nanoflakes triggers macroscale superlubricity. Tribol. Int..

[cit80] Guo L. (2021). *et al.*, MXene-Al_2_O_3_ synergize to reduce friction and wear on epoxy-steel contacts lubricated with ultra-low sulfur diesel. Tribol. Int..

[cit81] Yan H. (2020). *et al.*, Towards high-performance additive of Ti_3_C_2_/graphene hybrid with a novel wrapping structure in epoxy coating. Carbon.

[cit82] Steck J. G. (2019). *et al.*, Fabrication and tribological characterization of deformation-resistant nano-textured surfaces produced by two-photon lithography and atomic layer deposition. Tribol. Int..

[cit83] Samanta A. (2020). *et al.*, Roles of chemistry modification for laser textured metal alloys to achieve extreme surface wetting behaviors. Mater. Des..

[cit84] Hočevar M. (2020). *et al.*, The interaction between the osteosarcoma cell and stainless steel surface, modified by high-fluence, nanosecond laser pulses. Surf. Coat. Technol..

[cit85] Xu C. (2019). *et al.*, Enhancement of substrate-coating adherence of boron-doped diamond electrodes by nanosecond laser surface texturing pretreatment. Surf. Coat. Technol..

[cit86] Maldonado-Cortés D. (2019). *et al.*, Synergistic effect on the tribological properties of tool steel through the use of laser surface texturing channels and nanoparticles. Wear.

[cit87] Wang X. (2019). *et al.*, Fabrication of micro/nano-hierarchical structures for droplet manipulation *via* velocity-controlled picosecond laser surface texturing. Opt. Lasers Eng..

[cit88] Zhang J. (2019). *et al.*, Frictional properties of surface textures fabricated on hardened steel by elliptical vibration diamond cutting. Precis. Eng..

[cit89] Yusuf Y. (2020). *et al.*, Antibacterial properties of laser surface-textured TiO_2_/ZnO ceramic coatings. Ceram. Int..

[cit90] Vishnoi M., Kumar P., Murtaza Q. (2021). Surface texturing techniques to enhance tribological performance: a review. Surf. Interfaces.

[cit91] Zou H. (2022). *et al.*, Particle size effects on efficiency of surface texturing in reducing friction. Tribol. Int..

[cit92] Wos S., Koszela W., Pawlus P. (2020). The effect of graphite surface texturing on the friction reduction in dry contact. Tribol. Int..

[cit93] Huang J. (2022). *et al.*, Influence of surface structure/wettability on tribological properties of titanium. Tribol. Int..

[cit94] Qin L. (2018). *et al.*, Fabricating hierarchical micro and nano structures on implantable Co–Cr–Mo alloy for tissue engineering by one-step laser ablation. Colloids Surf., B.

[cit95] Tagawa N., Mori A. (2003). Thin film gas lubrication characteristics of flying head slider bearings over patterned media in hard disk drives. Microsyst. Technol..

[cit96] Hendricks T. J. (2010). *et al.*, Enhancement of pool-boiling heat transfer using nanostructured surfaces on aluminum and copper. Int. J. Heat Mass Transfer.

[cit97] Zhang D. (2016). *et al.*, Study on tribological properties of multi-layer surface texture on Babbitt alloys surface. Appl. Surf. Sci..

[cit98] Zhang H. (2020). *et al.*, Improving processing quality and tribological behavior of laser surface textures using oil layer method. Tribol. Int..

[cit99] Hsu S. M., Jing Y., Zhao F. (2015). Self-adaptive
surface texture design for friction reduction across the lubrication regimes. Surf. Topogr.:Metrol. Prop..

[cit100] Křupka I., Poliščuk R., Hartl M. (2009). Behavior of thin viscous boundary films in lubricated contacts between micro-textured surfaces. Tribol. Int..

[cit101] Arias P. (2020). Growth kinetics of two-dimensional hexagonal boron nitride layers on Pd (111). Nano Lett..

[cit102] Raayai-Ardakani S. (2022). A polynomial framework for design of drag reducing periodic two-dimensional textured surfaces. Int. J. Heat Fluid Flow.

[cit103] Erdemir A. (2005). Review of engineered tribological interfaces for improved boundary lubrication. Tribol. Int..

[cit104] Etsion I. (2005). State of the Art in Laser Surface Texturing. J. Tribol..

[cit105] Nilsson B. , *et al.*, Oil pockets and surface topography: mechanism of friction reduction, W: Proceedings of the XI International Colloquium on Surfaces*.* 2004

[cit106] Gachot C. (2017). *et al.*, A critical assessment of surface texturing for friction and wear improvement. Wear.

[cit107] Wang T. (2014). *et al.*, Experimental study of two-phase mechanical face Seals with laser surface texturing. Tribol. Int..

[cit108] Ma X. (2019). *et al.*, Suction effect of cavitation in the reverse-spiral-grooved mechanical face seals. Tribol. Int..

[cit109] Shinde A. (2018). *et al.*, Numerical Analysis of Deterministic Micro-Textures on the Performance of Hydrodynamic Journal Bearing. Mater. Today: Proc..

[cit110] Qiu M., Minson B. R., Raeymaekers B. (2013). The effect of texture shape on the friction coefficient and stiffness of gas-lubricated parallel slider bearings. Tribol. Int..

[cit111] Dan L. (2020). *et al.*, Tribological characteristics of a cemented carbide friction surface with chevron pattern micro-texture based on different texture density. Tribol. Int..

[cit112] Zou H. (2021). *et al.*, Efficiency of surface texturing in the reducing of wear for tests starting with initial point contact. Wear.

[cit113] Zhang K. (2022). *et al.*, Effects of composite textured surface on friction characteristics of 42CrMo steel under grease lubrication. Wear.

[cit114] Marian M. (2023). *et al.*, Combining multi-scale surface texturing and DLC coatings for improved tribological performance of 3D printed polymers. Surf. Coat. Technol..

[cit115] Shen C., Khonsari M. M. (2015). Numerical optimization of texture shape for parallel surfaces under unidirectional and bidirectional sliding. Tribol. Int..

[cit116] Tewelde F. B. (2023). *et al.*, Asymmetric surface texturing for directional friction control under dry sliding condition. Tribol. Int..

[cit117] Chen K., Tang Y. (2024). Research Progress on the Design of Surface Texture in Tribological Applications: A Mini-Review. Symmetry.

[cit118] Dan L. (2020). *et al.*, Tribological characteristics of a cemented carbide friction surface with chevron pattern micro-texture based on different texture density. Tribol. Int..

[cit119] Morris N. (2015). *et al.*, Combined Numerical and Experimental Investigation of the Micro-hydrodynamics of Chevron-based Textured Patterns Influencing Conjunctional Friction of Sliding Contacts. Proc. Inst. Mech. Eng., Part J.

[cit120] Zhang D. (2019). *et al.*, Effect of texture parameters on the tribological properties of spheroidal graphite cast iron groove-textured surface under sand-containing oil lubrication conditions. Wear.

[cit121] Mazur M. (2017). *et al.*, Numerical and experimental evaluation of a conformally cooled H13 steel injection mould manufactured with selective laser melting. Int. J. Adv. Des. Manuf. Technol..

[cit122] Ooi S., Bhadeshia H. K. D. H. (2012). Duplex Hardening of Steels for Aeroengine Bearings. ISIJ Int..

[cit123] Chi Y. (2018). *et al.*, Laser surface alloying on aluminum and its alloys: a review. Opt. Lasers Eng..

[cit124] Vasudev H. (2019). *et al.*, Microwave heating and its applications in surface engineering: a review. Mater. Res. Express.

[cit125] Sharma V., Prakash U., Kumar B. V. M. (2015). Surface composites by friction stir processing: a review. J. Mater. Process. Technol..

[cit126] Hao Y. (2011). *et al.*, Surface modification of Al–20Si alloy by high current pulsed electron beam. Appl. Surf. Sci..

[cit127] Guo D. (2020). *et al.*, Laminar plasma jet surface hardening of the U75V rail steel: insight into the hardening mechanism and control scheme. Surf. Coat. Technol..

[cit128] Guo D. (2021). *et al.*, Laminar plasma jet surface hardening of P20 mold steel: analysis on the wear and corrosion behaviors. Surf. Coat. Technol..

[cit129] Cao X. (2016). *et al.*, Design and Characteristics of a Laminar Plasma Torch for Materials Processing. Plasma Chem. Plasma Process..

[cit130] Miao J. (2015). *et al.*, Experimental Study on the Characteristics of a Miniature Laminar Plasma Torch with Different Gas Flow Patterns. Plasma Chem. Plasma Process..

[cit131] Xiang Y. (2017). *et al.*, Effects of thermal plasma surface hardening on wear and damage properties of rail steel. Proc. Inst. Mech. Eng., Part J.

[cit132] Xu J. (2020). *et al.*, An investigation into the microstructure and tribological properties of rail materials with plasma selective quenching. Tribol. Int..

[cit133] Pan W. X. (2005). *et al.*, Feasibility of laminar plasma-jet hardening of cast iron surface. Surf. Coat. Technol..

[cit134] Petrov S. V., Saakov A. G. (2002). Technology and equipment for plasma surface hardening of heavy-duty parts. Mater. Manuf. Processes.

[cit135] Kanaev A. T., Bogomolov A. V., Sarsembaeva T. E. (2012). Improving the wear resistance of wheel-pair rims by plasma quenching. Steel in Trans..

[cit136] Su F.-H., Zhang Z.-Z., Liu W.-M. (2008). Tribological behavior of hybrid glass/PTFE fabric composites with phenolic resin binder and nano-TiO_2_ filler. Wear.

[cit137] Li J., Ran Y. (2010). Evaluation of the friction and wear properties of PTFE composites filled with glass and carbon fiber. Materialwiss. Werkstofftech..

[cit138] Qian-qian S., Xian-hua C. (2007). Friction and wear of rare earths modified carbon fibers filled PTFE composite under dry sliding condition. Appl. Surf. Sci..

[cit139] Liu P. (2012). *et al.*, Tensile and tribological properties of polytetrafluroethylene homocomposites. Wear.

[cit140] Bandaru A. K. (2020). *et al.*, Mechanical and abrasive wear response of PTFE coated glass fabric composites. Wear.

[cit141] Shang C. (2020). *et al.*, Effect of network size on mechanical properties and wear resistance of titanium/nanodiamonds nanocomposites with network architecture. Compos. Commun..

[cit142] Li X. (2016). *et al.*, Fabrication of micro- and nano-scale hierarchical structures on Al surface with enhanced wettability, anti-corrosion and wear resistance. Mater. Express.

[cit143] Wang L.-F. (2014). *et al.*, Superlubricity of two-dimensional fluorographene/MoS_2_ heterostructure: a first-principles study. Nanotechnology.

[cit144] Gao K. (2022). *et al.*, Friction and wear behavior of bioinspired composites with nacre-like lamellar and brick-and-mortar architectures against human enamel. J. Mater. Sci. Technol..

[cit145] Cao H. (2021). *et al.*, High temperature tribological performance and thermal conductivity of thick Ti/Ti-DLC multilayer coatings with the application potential for Al alloy pistons. Diamond Relat. Mater..

[cit146] Beder M., Alemdag Y. (2021). Influence of Mg addition and T6 heat treatment on microstructure, mechanical and tribological properties of Al–12Si–3Cu based alloy. Trans. Nonferrous Met. Soc. China.

[cit147] Tang Y. (2021). *et al.*, Designing high-entropy ceramics *via* incorporation of the bond-mechanical behavior correlation with the machine-learning methodology. Cell Rep. Phys. Sci..

[cit148] Guo L. (2021). *et al.*, Tailoring M7C3 carbide *via* electron work function-guided modification. Scr. Mater..

[cit149] Tang Y., Li D. Y. (2021). Nano-tribological behavior of high-entropy alloys CrMnFeCoNi and CrFeCoNi under different conditions: A molecular dynamics study. Wear.

[cit150] Tang Y., Li D. Y. (2023). Influences of C, Si and Mn on the wear resistance of coiled tubing steel. Wear.

[cit151] Yousefi D., Taghiabadi R., Shaeri M. H. (2021). Effect of multi-pass multi-directional forging on tribological properties of Si-rich eutectoid ZA alloys. Trans. Nonferrous Met. Soc. China.

[cit152] Bondarev A. V. (2017). *et al.*, Tribological behavior and self-healing functionality of TiNbCN-Ag coatings in wide temperature range. Appl. Surf. Sci..

[cit153] Singh H., Kumar S., Kumar D. (2020). The role of in-situ ceramic reinforcements on microstructure evolution and mechanical properties on developed hybrid Mg-MMCs. Mater. Sci. Eng. A.

[cit154] Liu R. (2021). *et al.*, Application of ultrasonic nanocrystal surface modification (UNSM) technique for surface strengthening of titanium and titanium alloys: a mini review. J. Mater. Res. Technol..

[cit155] Wang H. (2019). *et al.*, Grain size effect on wear resistance of WC-Co cemented carbides under different tribological conditions. J. Mater. Sci. Technol..

[cit156] El Aal M. I. A. (2020). The influence of ECAP and HPT processing on the microstructure evolution, mechanical properties and tribology characteristics of an Al6061 alloy. J. Mater. Res. Technol..

[cit157] Xin B. (2021). *et al.*, Improving mechanical properties and tribological performance of Al_0.2_Co_1.5_CrFeNi_1.5_Ti_0.5_ high entropy alloys *via* doping Si. J. Alloys Compd..

[cit158] Yang R. (2021). *et al.*, Effect of hot rolling on microstructure and tribology behaviors of Ti–50.8Ni alloy. Trans. Nonferrous Met. Soc. China.

[cit159] Sam M., Radhika N. (2022). Influence of carbide ceramic reinforcements in improving tribological properties of A333 graded hybrid composites. Def. Technol..

[cit160] Tuan L. H., Sang L. V. (2023). Annealing coatings of graphene on silicon and application to tribology. Tribol. Int..

[cit161] Yang X., Wang Y., Zhang J. (2023). Scaling up to macroscale superlubricity of sp2-dominated structural carbon films: graphene and carbon onion. Appl. Surf. Sci..

[cit162] Zhang Z. (2021). *et al.*, Effect of diamond grain size on the tribological properties of WS2-based multilayer coatings. Diamond Relat. Mater..

[cit163] Gan Y. (2023). *et al.*, Tailoring the tribology property and corrosion resistance of selective laser melted CoCrMo alloys by varying copper content. Mater. Des..

[cit164] Wan Q. (2023). *et al.*, Revealing the B addition on tribology performance in TiZrHfTa0.5 refractory high-entropy alloy at ambient and elevated temperature. J. Alloys Compd..

[cit165] Karamimoghadam M. (2024). *et al.*, Laser Surface Transformation Hardening for Automotive Metals: Recent Progress. Metals.

[cit166] Niu G. (2024). *et al.*, Effect of retained austenite on impact-abrasion wear performance of high-strength wear-resistant steel prepared by dynamic partitioning process. Wear.

[cit167] Tabatabaei F. S. K. (2023). *et al.*, The effect of WC-CoCr content on hardness and tribological properties of NiCrBSi coatings fabricated by the HVOF process. Surf. Coat. Technol..

[cit168] Schnell G., Müller T., Seitz H. (2023). Tribological effects of different scaled chevron-shaped microstructures on the Stribeck curve of parallel contacts under unidirectional friction. Tribol. Int..

[cit169] Xiao Q. (2023). *et al.*, High-temperature tribological properties of coatings repaired by laser additive manufacturing on railway wheel tread damage. Wear.

[cit170] Hu M.-j. (2023). *et al.*, Comparative study of the current-carrying tribological properties of carbon graphite composites with different hardnesses. Int. J. Mech. Sci..

[cit171] Maytorena-Sánchez A. (2021). *et al.*, Analysis of the hardness and tribological properties of grade 2 titanium using the thermal oxidation process at different temperatures. Mater. Lett..

[cit172] Ding H. (2022). *et al.*, Effect of laser claddings of Fe-based alloy powder with different concentrations of WS2 on the mechanical and tribological properties of railway wheel. Wear.

[cit173] Mosbacher M. (2022). *et al.*, Oxygen diffusion hardened zirconium alloy ZrNb7 – Tribological properties derived from Calo wear and wheel on flat experiments. Tribol. Int..

[cit174] Piasecki A. (2023). *et al.*, Microstructure, mechanical properties and tribological behavior of Cu-nano TiO_2_-MWCNTs composite sintered materials. Wear.

[cit175] He D. (2023). *et al.*, Role of carbide content in governing the mechanical and tribological properties of DLC/Cr3C2–NiCr duplex coatings. Tribol. Int..

[cit176] Wan C. (2023). *et al.*, Mechanical and tribological behaviors of MWCNT/polyimide nanocomposites under drift of glass transition temperature. Tribol. Int..

[cit177] Zhang J. (2023). *et al.*, Integrated printing of high-strength, high-shape-retaining polyimide and its composite gradient structures for enhanced tribological properties. Addit. Manuf..

[cit178] Tu Y. (2023). *et al.*, Improving the mechanical and tribological behavior of Cu-WS2 self-lubricating composite with the addition of WS2 nanosheet. Wear.

[cit179] Jungk J., Michael J., Prasad S. (2008). The role of substrate plasticity on the tribological behavior of diamond-like nanocomposite coatings. Acta Mater..

[cit180] Six K. (2016). *et al.*, Classification and consideration of plasticity phenomena in wheel-rail contact modelling. Int. J. Railw. Technol..

[cit181] Jayakrishna K. , *et al.*, 1 – Materials selection for aerospace components, in Sustainable Composites for Aerospace Applications, ed. M. Jawaid and M. Thariq, Woodhead Publishing, 2018, pp. 1–18

[cit182] Aamir M. (2020). *et al.*, A review: drilling performance and hole quality of aluminium alloys for aerospace applications. J. Mater. Res. Technol..

[cit183] Xu C. (2023). *et al.*, Surface protection of a V–4Cr–4Ti alloy through a multilayered TiAl/TiAlN composite coating. Vacuum.

[cit184] Ma X. (2021). *et al.*, Flexural strength and wear resistance of C/C–SiC brake materials improved by introducing SiC ceramics into carbon fiber bundles. Ceram. Int..

[cit185] Feng X. (2022). *et al.*, Effect of a micro-textured surface with deposited MoS_2_-Ti film on long-term wear performance in vacuum. Surf. Coat. Technol..

[cit186] Safi S., Kazemzadeh A. (2013). MCMB–SiC composites; new class high-temperature structural materials for aerospace applications. Ceram. Int..

[cit187] Mufti T. A. (2023). *et al.*, Development, mechanical characterization and high temperature tribological evaluation of magnetron sputtered novel MoS_2_-CaF2-Ag coating for aerospace applications. Tribol. Int..

[cit188] Shaik D. (2021). *et al.*, Tribological behavior of friction stir processed AA6061 aluminium alloy. Mater. Today: Proc..

[cit189] Ren X. (2023). *et al.*, Surface modification technologies for enhancing the tribological properties of cemented carbides: a review. Tribol. Int..

[cit190] Seikh Z. (2021). *et al.*, Density, hardness and wear responses of aluminium-copper-magnesium alloys. Mater. Today: Proc..

[cit191] Sharma R., Sharma K., Kumar Saraswat B. (2023). A review of the mechanical and chemical properties of aluminium alloys AA6262 T6 and its composites for turning process in the CNC. Mater. Today: Proc..

[cit192] Ashiri R., Niroumand B., Karimzadeh F. (2014). Physical, mechanical and dry sliding wear properties of an Al–Si–Mg–Ni–Cu alloy under different processing conditions. J. Alloys Compd..

[cit193] Pancrecious J. K. (2018). *et al.*, Nanoceria induced grain refinement in electroless Ni-B-CeO_2_ composite coating for enhanced wear and corrosion resistance of Aluminium alloy. Surf. Coat. Technol..

[cit194] Ma Z. (2023). *et al.*, Microstructure, mechanical property and corrosion resistance of FeCoCrNi-M high-entropy alloy coatings on 6061 aluminum alloy prepared by laser cladding. Surf. Coat. Technol..

[cit195] Zhang Y. (2023). *et al.*, Microstructure and wear resistance of direct laser-deposited TiC-enhanced aluminum-based composite coating for brake discs. Surf. Coat. Technol..

[cit196] Holmberg K., Erdemir A. (2017). Influence of tribology on global energy consumption, costs and emissions. Friction.

[cit197] Li Z. (2021). *et al.*, Improving surface resistance to wear and corrosion of nickel-aluminum bronze by laser-clad TaC/Co-based alloy composite coatings. Surf. Coat. Technol..

[cit198] Chen C. (2022). *et al.*, Fatigue behavior and tribological properties of laser additive manufactured aluminum alloy/boron nitride nanosheet nanocomposites. J. Mater. Res. Technol..

[cit199] Jin P. (2021). *et al.*, Realization of synergistic enhancement for fracture strength and ductility by adding TiC particles in wire and arc additive manufacturing 2219 aluminium alloy. Composites, Part B.

[cit200] Kwok K. C. S., Hu G. (2023). Wind energy system for buildings in an urban environment. J. Wind. Eng. Ind. Aerodyn..

[cit201] Song J. (2017). *et al.*, Performance of a circular cylinder piezoelectric wind energy harvester fitted with a splitter plate. Appl. Phys. Lett..

[cit202] Kong K. (2023). *et al.*, Progress and Trends in Damage Detection Methods, Maintenance, and Data-driven Monitoring of Wind Turbine Blades – A Review. Renew. Energy Focus.

[cit203] Zhang L.-B. (2023). *et al.*, Nano-silica anti-icing coatings for protecting wind-power turbine fan blades. J. Colloid Interface Sci..

[cit204] Mathavan J. J., Patnaik A. (2020). Analysis of wear properties of granite dust filled polymer composite for wind turbine blade. Results Mater..

[cit205] Palacio M., Bhushan B. (2007). Wear detection of candidate MEMS/NEMS lubricant films using atomic force microscopy-based surface potential measurements. Scr. Mater..

[cit206] Nagai T. (2021). *et al.*, Anticorrosion of DLC coating in acid solutions. Appl. Surf. Sci..

[cit207] Yan M. (2020). *et al.*, Friction and wear properties of GLC and DLC coatings under ionic liquid lubrication. Tribol. Int..

[cit208] Liu S. (2022). *et al.*, Interlayer graphene oxide/binary ionic liquids composite lubricating films with improved load-carrying and anti-wear properties. Thin Solid Films.

[cit209] Kong X. (2020). *et al.*, Study of tip wear for AFM-based vibration-assisted nanomachining process. J. Manuf. Process..

[cit210] Zavedeev E. V. (2018). *et al.*, Effects of AFM tip wear on frictional images of laser-patterned diamond-like nanocomposite films. Wear.

[cit211] Gou L. Q. (2012). *et al.*, Composite diamond-DLC coated nanoprobe tips for wear resistance and adhesion reduction. Surf. Coat. Technol..

[cit212] Kruzic J. J., Hoffman M., Arsecularatne J. A. (2023). Fatigue and wear of human tooth enamel: a review. J. Mech. Behav. Biomed. Mater..

[cit213] Hoque M. E. (2022). *et al.*, Titanium and titanium alloys in dentistry: current trends, recent developments, and future prospects. Heliyon.

[cit214] Koizumi H. (2019). *et al.*, Application of titanium and titanium alloys to fixed dental prostheses. J. Prosthodont. Res..

[cit215] Shah A. , *et al.*, Surface Modification on Titanium Alloy for Biomedical Applications, in Encyclopedia of Smart Materials, ed. A.-G. Olabi, Elsevier, Oxford, 2018. pp. 436–444

[cit216] Bao Y. (2021). *et al.*, Corrosion resistance and antibacterial activity of Ti–N–O coatings deposited on dental titanium alloy. Surf. Coat. Technol..

[cit217] Abitha H. (2020). *et al.*, A recent investigation on shape memory alloys and polymers based materials on bio artificial implants-hip and knee joint. Mater. Today: Proc..

[cit218] Zhang Y. J. (2018). *et al.*, TiCuN solid solution coating: excellent wear-resistant biocompatible material to protect artificial joint. Mater. Lett..

[cit219] Cui W. (2020). *et al.*, Structural and tribological characteristics of ultra-low-wear polyethylene as artificial joint materials. J. Mech. Behav. Biomed. Mater..

[cit220] Wu T. (2022). *et al.*, The antibacterial and wear-resistant nano-ZnO/PEEK composites were constructed by a simple two-step method. J. Mech. Behav. Biomed. Mater..

[cit221] Liu D. (2022). *et al.*, Simultaneous enhancement of anti-friction and wear resistance performances *via* porous substrate and FeCoNiTiAl high entropy alloy coating of artificial joint materials. J. Mater. Res. Technol..

[cit222] Corona-Gomez J. (2021). *et al.*, Wear and corrosion characteristics of nano-crystalline tantalum nitride coatings deposited on CoCrMo alloy for hip joint applications. Mater. Charact..

[cit223] Corona-Gomez J., Yang Q. (2022). Wear and corrosion characterization of single and multilayered nanocrystalline tantalum coatings on biomedical grade CoCrMo alloy. Materialia.

